# Quality of ChatGPT-Generated Therapy Recommendations for Breast Cancer Treatment in Gynecology

**DOI:** 10.3390/curroncol31070284

**Published:** 2024-07-01

**Authors:** Jan Lennart Stalp, Agnieszka Denecke, Matthias Jentschke, Peter Hillemanns, Rüdiger Klapdor

**Affiliations:** 1 Department of Obstetrics and Gynecology, Hannover Medical School, 30625 Hannover, Germany; 2 Department of Obstetrics and Gynecology, Albertinen Hospital Hamburg, 22457 Hamburg, Germany

**Keywords:** ChatGPT, breast cancer, oncology, artificial intelligence, gynecological cancer

## Abstract

**Introduction:** Artificial intelligence (AI) is revolutionizing medical workflows, with self-learning systems like ChatGPT showing promise in therapy recommendations. Our study evaluated ChatGPT’s performance in suggesting treatments for 30 breast cancer cases. AI’s role in healthcare is expanding, particularly with tools like ChatGPT becoming accessible. However, understanding its limitations is vital for safe implementation. **Material and Methods:** We used 30 breast cancer cases from our medical board, assessing ChatGPT’s suggestions. The input was standardized, incorporating relevant patient details and treatment options. ChatGPT’s output was evaluated by oncologists based on a given questionnaire. **Results:** Treatment recommendations by ChatGPT were overall rated sufficient with minor limitations by the oncologists. The HER2 treatment category was the best-rated therapy option, with the most accurate recommendations. Primary cases received more accurate recommendations, especially regarding chemotherapy. **Conclusions:** While ChatGPT demonstrated potential, difficulties were shown in intricate cases and postoperative scenarios. Challenges arose in offering chronological treatment sequences and partially lacked precision. Refining inputs, addressing ethical intricacies, and ensuring chronological treatment suggestions are essential. Ongoing research is vital to improving AI’s accuracy, balancing AI-driven suggestions with expert insights and ensuring safe and reliable AI integration into patient care.

## 1. Introduction

The impact of artificial intelligence (AI) on modern medical workflows is steadily increasing [1]. Self-learning systems, such as image recognition in pathology or radiology, are already widely used. Additionally, AI-based systems have been implemented in clinical devices such as ultrasounds to improve examination quality, for instance, in gynecology and obstetrics [2]. Since this research is often lacking clinical applicability [2], these systems are not implemented in daily medical treatment routines, therapy recommendations or patient care in Germany. However, AI-based chatbots are already freely available to the general public and not limited to experienced or trained users. As such a system, ChatGPT developed by OpenAI Inc. (San Francisco, CA, USA) is of great interest in almost every scientific and non-scientific field. As a Large Language Model (LLM), ChatGPT is capable of processing data, generating language and reproducing knowledge. LLMs are based on deep-learning algorithms to even generate new output by combining the training input. In the case of ChatGPT, the system’s training data vary depending on the model in use and have been updated regularly in the last year. Since the training data contain medical knowledge as well, the system can, therefore, generate medical treatment recommendations based on oncological guidelines. The capability to combine the input (e.g., patient cases) with the training data could then lead to possible treatment advice. As modern healthcare faces multiple challenges in patient care due to staff shortages and a rapidly increasing number of trials and treatment options, AI systems could be a valuable tool to support medical decision-making [3,4]. With access to the latest medical research, ChatGPT could generate an interdisciplinary tumor board-like therapy recommendation to optimize workflow. In order to ensure patient safety and quality of care, these systems and their current capabilities need to be carefully evaluated. How LLMs can help to reduce workload burden and burnout has recently been shown [5].

In the field of gynecology, various cancer entities exist to evaluate the recommendation quality of ChatGPT for oncologic diseases. Of those malignancies, breast cancer is the second most common for both sexes and the deadliest for women [6]. Due to the broad spectrum of therapy options, various medical specialties are involved in determining accurate guideline-based therapy in interdisciplinary tumor boards. This offers the opportunity to test the capability of ChatGPT to recommend a therapy regimen for complex breast cancer cases with multiple treatment options to consider. Due to the multiple treatment strategies for breast cancer, including surgery, radiation and hormone therapy, all provided in one guideline, this creates an opportunity to test the quality of ChatGPT’s data processing and output abilities in a multi-option scenario.

**Figure 1:**
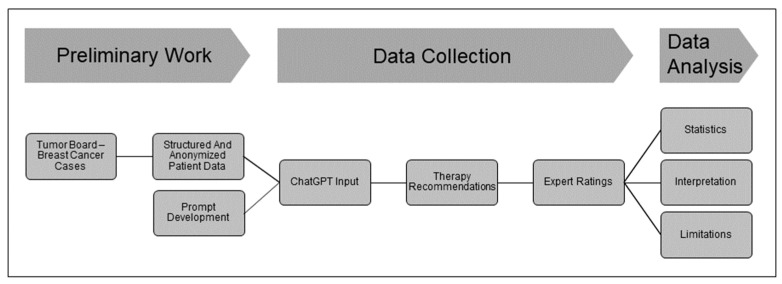
Study design.

Therefore, we asked ChatGPT to suggest therapy schemes for 30 consecutive breast cancer patients from our cancer board in 2022. To assess the accuracy of the recommendations, we asked four specialized gynecologic oncologists to rate the treatment suggestions based on a given evaluation form. Our study (Figure 1) will show the quality of ChatGPT’s given recommendations and identify sources of error that need to be addressed to improve performance.

## 2. Materials and Methods

### 2.1. Patient Cases

A total of 30 consecutive cases of breast cancer patients from the Interdisciplinary Gynecologic Tumor Board of the Hannover Medical School in 2022 were used for evaluation via ChatGPT (GPT 3.5). We included patients with primary or recurrent invasive breast cancer. Cases with already completed therapy were excluded. The cases were anonymized for personal details, such as date of birth, origin or treatment dates. Afterwards, data was structured in a predefined form to improve the overview for the experts without editing the patient’s medical history or treatment data.

### 2.2. Data Distribution

Of the 30 patients, 9 (30%) were initially diagnosed with breast cancer and had not received any prior treatment, 15 patients (50%) were pretreated (surgery or chemotherapy), 4 patients (13.3%) suffered from their first relapse and 2 patients (6.7%) were diagnosed with their second relapse. The ages of the patients ranged from 31 to 88 years (Table 1).

Out of the analyzed cases, 28 (93.3%) were histologically confirmed invasive breast cancer, 1 (3.3%) ductal carcinoma in situ (DCIS) and 1 (3.3%) phyllodes tumor. Within the invasive cancer group, 24 (80%) were estrogen receptor-positive, 19 (63%) progesterone receptor-positive and 3 (10.7%) HER-2 receptor-positive (Table 1).

### 2.3. ChatGPT Prompt

We used the GPT-3.5 model with training data up to September 2021 at the time of prompting, accessed via a web interface for all preliminary work and final analyses. A test case was used to optimize the *prompt* design and output quality until ChatGPT provided consistent answer content for repeated input. The output format was assessed in different styles, such as text, bullet point or table output. Due to a better overview and more detailed suggestions, the *prompt* was designed with a combinatory table and text output. As a source of information, we included the URL of the latest German breast cancer guideline [7] and, therefore, used German *prompts* to avoid translation errors. The integration of the URL functioned as an active lead for ChatGPT towards the guideline. An evaluation of the code itself did not take place. The patient’s history was structured in a standardized pattern to provide equal input for all cases. These included key facts such as age, histology, tumor stage, molecular markers, relevant medical history and already conducted tumor-specific treatments always implemented in the same order and wording. The output was requested in the form of a table with treatment options of surgery, chemotherapy, radiotherapy, HER-2 therapy and endocrine therapy. For each treatment category, ChatGPT should include a general recommendation (yes/no), medications and dosages (if applicable), alternative options, and therapy-related risks. Additionally, ChatGPT was asked to summarize the therapy recommendation in one short sentence, equivalent to a tumor board decision (translated medical histories, *prompts* and recommendations shown in File S1). No further *prompt* adjustments were made after the evaluation of the main cases began despite the errors identified. All prompting was performed on 10 July 2023.

### 2.4. Tumor Board Setting and ChatGPT Recommendations

The recommendations should mimic a tumor board-like decision for breast cancer cases. This would include an oncological surgeon, a gynecological oncologist, a radiotherapist, a pathologist and, if images are to be displayed, a radiologist to discuss the tumor biology, the treatment options and consent to an overall therapy sequence. Afterwards, treatment steps would be performed in the consented order with necessary adaptations due to the medical condition of the patient.

The possible therapy options were, therefore, surgery, chemotherapy, radiotherapy, HER-2 therapy and endocrine therapy. Every given recommendation included a specific procedure or medication to apply as well as an overview of the major risks for those interventions. Since various recommendations can be considered valid, the data classification is multi-label.

### 2.5. Gynecological Oncologists and Evaluation Form

For the evaluation of the ChatGPT output, four gynecological oncologists, all consultants and specialists for gynecologic oncology at the Department of Obstetrics and Gynecology of the Hannover Medical School, were selected to evaluate the recommendations. They received the patient data in a standardized structure with the recommendation from ChatGPT (File S1). The evaluation was performed by a structured questionnaire rating each treatment option individually, as well as the overall recommendation (File S2).

### 2.6. Data Analysis

The rating categories were converted to a numerical scale (*correct without limitations* = 1, *correct with minor limitations* = 2, *better alternative available* = 3, *partially wrong* = 4, and *completely wrong* = 5) for data analysis. A T-test was used to test for differences in accuracy between treatment options. *p*-values of <0.05 were judged to be significant (* *p* < 0.05, ** *p* < 0.005, *** *p* < 0.001).

## 3. Results

**Figure 2:**
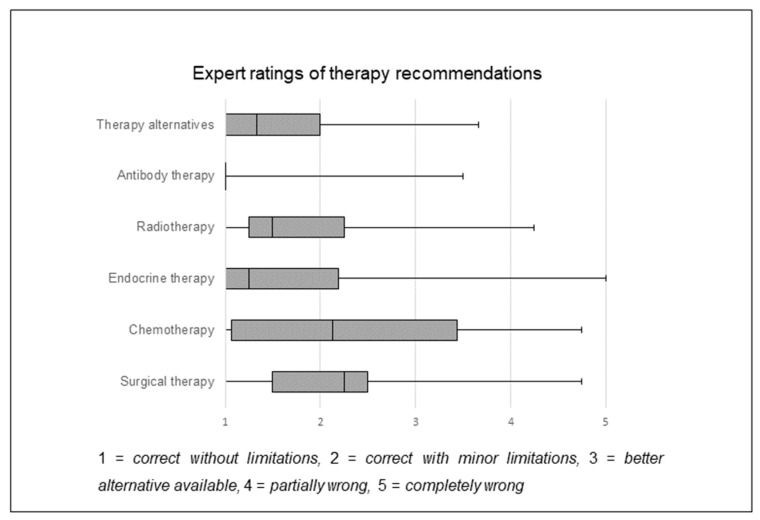
Therapy recommendations for breast cancer patients (n = 30) out of the interdisciplinary tumor board generated by ChatGPT were rated by specialized gynecologic oncologists. All therapy recommendations show an overall sufficient validity. Experts rated the suggestions with the following scale: *correct without limitations* = 1, *correct with minor limitations* = 2, *better alternative available* = 3, *partially wrong* = 4 and *completely wrong* = 5. Boxplots include marked median.

A total of 30 cases with breast cancer patients were transformed into ChatGPT *prompts* and sent to ChatGPT for treatment recommendation. Patient details and output frequencies are summarized in Table 1 and Table 2. ChatGPT suggested surgery in 27 cases (90%), chemotherapy in 21 cases (70%), radiotherapy in 27 cases (90%), HER-2 therapy in 3 cases (10%) and endocrine therapy in 20 cases (66.7%) (Table 2). Afterwards, all data and outputs were forwarded to the experts with the predefined questionnaire for evaluation. The rating scores were analyzed and plotted as means (Figure 2).

During the *prompt* preparation, we tested different types of wording and structures of the patient’s medical history to improve the output. Since the experts did not evaluate the preliminary work, no statistical analysis could be performed. The most accurate output was produced when the patient data were given in precise, short sentences. For diagnosis-related details such as TNM classification or HER-2 receptor status, correct recommendations were obtained when the wording was equivalent to the cancer guideline in use. For HER-2 status, the format positive or negative was required for valid output.

**Figure 3:**
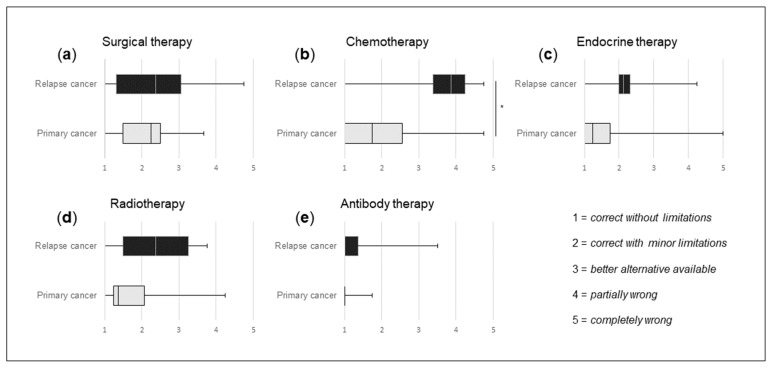
Analyses of quality differences in the recommendations for primary (24 patients) vs. relapse breast cancer (6 patients). (**a**) Ratings of surgical therapy recommendations for primary vs. relapse breast cancer. (**b**) Ratings of chemotherapy recommendations for primary vs. relapse breast cancer. (**c**) Ratings of endocrine therapy recommendations for primary vs. relapse breast cancer. (**d**) Ratings of radiotherapy recommendations for primary vs. relapse breast cancer. (**e**) Ratings of antibody therapy recommendations for primary vs. relapse breast cancer. Experts rated the suggestions with the following scale: *correct without limitations* = 1, *correct with minor limitations* = 2, *better alternative available* = 3, *partially wrong* = 4 and *completely wrong* = 5. Boxplots include marked median. * *p* < 0.05 (Student’s T-test).

As shown in Figure 2, the treatment options of surgery, chemotherapy, endocrine therapy and radiation were rated as *correct with minor limitations*, indicating an overall sufficient recommendation based on the guideline of interest without significant differences. The treatment recommendation for HER-2 antibody therapy was rated as *correct without limitations*, with a significantly better score than any of the evaluated treatment options. To ensure comparability of the expert ratings, we evaluated the ratings of any two observers individually for every category. Significant differences were seen twice in the category therapy alternatives (Figure S1). When comparing primary and relapse cases, there is a trend towards more accurate recommendations for all treatment categories in primary cases (Figure 3). ChatGPT recommendations regarding chemotherapy were significantly better for primary cases than for relapse cases (Figure 3b).

Focusing on the accuracy of ChatGPT’s recommendations in relation to the *prompt* used, it is noticeable that the output varies with identical input. This results in recommendations with duplicate sentences, missing medication details, or incorrect suggestions. In addition, ChatGPT suggested surgical treatment for postoperative cases. Of the 30 cases, 14 had already undergone surgery, and 5 had subsequent R1 resection. The AI recommended surgery in all 14 cases without identifying the condition after surgery. For the cases with incomplete resection, the recommendation was considered correct accordingly. However, the cases requiring a second surgery were not explicitly identified. In addition, all recommendations lack a chronological sequence of therapy options to integrate them into clinical routine. ChatGPT correctly names the relevant therapies but does not place them in a necessary therapy scheme.

## 4. Discussion

In our study, we conducted an evaluation of the potential and pitfalls of using ChatGPT to provide guideline-compliant therapy recommendations for breast cancer patients. We were able to demonstrate that ChatGPT is capable of generating therapy recommendations that were judged to be mostly accurate by experts in the field of gynecologic oncology. In addition, we showed that the quality of recommendations is higher for primary cases than for relapse cases.

AI-based systems have become a key factor in almost every scientific innovation. So far, these systems have been used to optimize workflow or support daily work routines in modern healthcare [5,8]. In terms of patient care, the current use of LLMs is rather limited. With ChatGPT as a publicly accessible LLM, the variety of users ranges from children to medical professionals in every field and setting as well as the potential use. In order to take advantage of these opportunities in the near future, the current capabilities and risks of using ChatGPT in a clinical context need to be evaluated. Initial studies in the field of gynecology have already shed light on some of the possibilities [9,10,11,12].

Focusing on the hormone and HER-2 receptor status of the analyzed cases, our test population shows a similar distribution to the reported frequencies of these characteristics for larger study groups [13,14]. Even though the number of cases is limited, it can be considered sufficient for this evaluation.

In contrast to the results of Lukac et al., 2023, who used primary breast cancer cases and compared it with their multidisciplinary tumor board recommendation, we found the HER-2 treatment recommendation to be the most reliable one [11]. In comparison to Lukac et al., 2023, we used a more complex *prompt* design and a broad spectrum of scenarios, which could explain the discrepancy in our findings. This discrepancy highlights the importance of *prompt* engineering before implementing AI tools in patient care. The quality and accuracy of the input determines whether the AI is able to generate a sufficient suggestion. A similar result was obtained by Griewing et al., 2023, who were able to improve the accuracy of the recommendations by using an extended input model [12]. Since we included the breast cancer guideline as a favored source of information in the *prompt*, it seemed to be mandatory to match the guideline’s wording (positive or negative) to achieve valid results. Still, it is crucial to keep in mind that limiting ChatGPT to the guideline is not possible, as the decision-making itself cannot be traced. Therefore, engineering the *prompt* equivalently could have led to correct processing, as shown for HER-2 therapy. Since *prompt* engineering is a relatively new field and new territory for medical professionals, sufficient training is mandatory to receive valid results. First tutorials for medical professionals already exist [15]. Nevertheless, owing to the outdated guideline from 2021, the recommendations lacked full accuracy concerning current scientific evidence. Notably, immune checkpoint inhibitors and CDK 4/6 inhibitors, absent from the guideline, were consequently omitted by ChatGPT. Despite these limitations, this study marks a demonstration of therapy recommendations related to a written guideline. Subsequent investigations are imperative to corroborate these findings using the GPT-4 model and align with the most recent therapy standards. Comparable statements have been published recently in various scenarios, indicating the need for follow-up work to improve reliability [15,16,17].

The study included both primary and relapse breast cancer cases sampled consecutively. Primary cases consistently showed better ratings in all treatments, especially regarding chemotherapy, where AI recommendations outperformed those for relapse cases significantly. The results raise questions about improving ChatGPT’s accuracy for intricate cases. Similar to a recent study [18], our findings highlight a decrease in reliability for complex medical decisions, emphasizing the need for further development. Due to the rapid changes in this field, additional studies with newer models of ChatGPT (GPT-4 and GPT-4o) and/or competing providers are mandatory.

In contrast, personalized recommendations were tailored based on the patient’s age and existing health conditions. For an elderly patient with metastatic disease (case 20, File S1), options such as radiotherapy and surgery were excluded from the recommendation due to the patient’s age. Addressing age-related denial of treatment involves complex ethical considerations that extend beyond medical records to ensure quality of care and patient safety. Despite this complexity, ChatGPT demonstrated the ability to customize suggestions according to individual patient profiles. However, expert opinions on this case varied significantly while remaining comparable across almost all categories during the study (Figure S1), highlighting its controversial nature. The increasing use of AI systems in medicine and the ethical challenges they pose are of utmost relevance today [19].

Despite this patient-specific adjustment, the recommendations have inaccuracies related to surgical therapies that have already been completed. Suggestions for postoperative cases sometimes advocated unsuitable procedures like mastectomy or breast-preserving surgery, even though patients had already undergone surgery during the initial diagnosis. Moreover, cases necessitating re-excision were not identified, indicating a limitation in the system’s understanding of surgical history. In line with these results, the LLM did not provide therapy recommendations in a treatment order; for instance: 1. surgery, 2. radiotherapy, 3. endocrine therapy. Instead, it focused on essential prerequisites, like prescribing radiation after breast-conserving surgery. Since the sequence in which certain therapy steps are applied is crucial in the treatment of breast cancer, this needs to be improved for clinical implication. The lack of a specific request for chronological order in the *prompt* could explain this inaccuracy, suggesting that it could be corrected with a revised input. Nevertheless, this circumstance needs to be further investigated, and comparable studies have already provided approaches here [12]. Likewise, since the engineered *prompt* and the guideline did not include therapy options like checkpoint inhibitors, ChatGPT refrained from making corresponding recommendations. This issue is likely to be resolved by refining the *prompt* design and using recent models, yet further efforts are necessary to ascertain the boundaries of AI-generated suggestions. Due to the rapid development in this field, reliable procedures do not yet exist, but initial reviews provide possible recommendations for clinical integration [20].

Focusing on the non-medical aspects of the output in terms of content and structure, it is noticeable that this varies even when the input is identical. For example, although the *prompt* requested a dosage suggestion for each recommended medication, ChatGPT only provided this in 4 of 21 chemotherapy recommendations. In addition, the recommendations were occasionally repeated despite the request not to generate text repetitions within an output. Because the output is based on a statistical process that results in a non-deterministic response pattern, these variations are well explained [15]. Although these are minor issues, they demonstrate the inconsistency of recommendation quality that can be fatal in a clinical context.

As AI systems become prominent in healthcare, understanding their limitations is crucial. The present study included 30 cases of breast cancer patients, which only gives a first insight into capabilities and pitfalls. To sufficiently prove the quality of the recommendations, a significantly higher number of scenarios and different tumor entities would be necessary. Nevertheless, combined with the existing literature, the expertise in this field is growing rapidly [9,10,11,12]. The GPT-3.5 model used is already outdated, but when comparing the output with the guideline applicable at the timepoint of the tumor board meeting in 2022, this is of minor relevance. The necessary knowledge to generate sufficient recommendations was part of the training data time span and is, therefore, no limitation for the output. In addition, as a free-of-charge version, GPT-3.5 may acquire a permanent user base, so further research is needed. Still, the most recent models could be more accurate due to improvements in data processing. Future research is needed to verify the results with updated models and to compare models from different providers. When working with LLMs, the production of output that cannot be explained by the training data, known as hallucinations, can occur and generate incorrect statements [21]. Due to the study design, it is unclear whether wrong therapy recommendations are based on hallucinations, incorrect processing or insufficient *prompt* design. Since we focused on sufficient recommendations, existing hallucinations with wrong output were considered incorrect accordingly. Furthermore, since ChatGPT is owned by a private company, modification of the model is possible, but it is limited at a certain point. This will allow sufficient implementation in daily clinical routines in the future, but the first approaches were promising [5].

## 5. Conclusions

This study illuminates ChatGPT’s potential in breast cancer therapy recommendations but reveals challenges in handling complex patient histories and stresses the importance of sufficient *prompt* engineering. Together with recent studies in the field of gynecology, insight is provided into the massive potential that LLM-supported decision-making has, and the ongoing need for research is highlighted [9,10,11,12]. Future research must focus on refining inputs, addressing surgical history, and chronological order, which would enhance AI’s precision. Ethical considerations, especially in age-related treatments, require a delicate balance between AI-driven suggestions and expert opinions, underscoring the necessity for nuanced decision-making. These efforts will ensure more reliable and context-appropriate AI-driven therapy suggestions, shaping a promising future for AI in patient care.

## Figures and Tables

**Table 1:** Patient characteristics.

	Number of Patients
Breast cancer history	
Primary breast cancer without treatment	9
Primary breast cancer with initial treatment	15
First relapse of breast cancer	4
Second relapse of breast cancer	2
Histology	
Invasive breast cancer	28
Ductal carcinoma in situ (G3)	1
Phyllodes tumor	1
Immunohistochemistry	
Estrogen receptor-positive	24
Estrogen receptor-negative	4
Progesterone receptor-positive	19
Progesterone receptor-negative	9
HER-2 receptor-positive	3
HER-2 receptor-negative	25
Age	31–88 years

**Table 2:** Overview of ChatGPT recommendations.

Treatment Categories	Frequency of Recommendation
Surgery	
Yes	27
No	3
Chemotherapy	
Yes	21
No	9
Radiotherapy	
Yes	27
No	3
HER-2 therapy	
Yes	3
No	27
Endocrine therapy	
Yes	20
No	10

## Data Availability

The original contributions presented in the study are included in the article/Supplementary Materials; further inquiries can be directed to the corresponding author.

## Supplementary Materials

The following supporting information can be downloaded at: https://www.mdpi.com/article/10.3390/curroncol31070284/s1, Figure S1: Inter-rater variance analyses; File S1: Patient cases; File S2: Evaluation form.
