# Supplementary material for: Quality of ChatGPT-Generated Therapy Recommendations for Breast Cancer Treatment in Gynecology
Source: Curr Oncol. 2024 Jul 1;31(7):3845–54. doi: 10.3390/curroncol31070284 (PMC11275284; doi:10.3390/curroncol31070284)
Supplement: Supplementary file 1 [file curroncol-31-00284-s001.zip › Figure S1_Inter-rater variance analyses.pdf]

Figure S1 – Inter-rater variance analyses

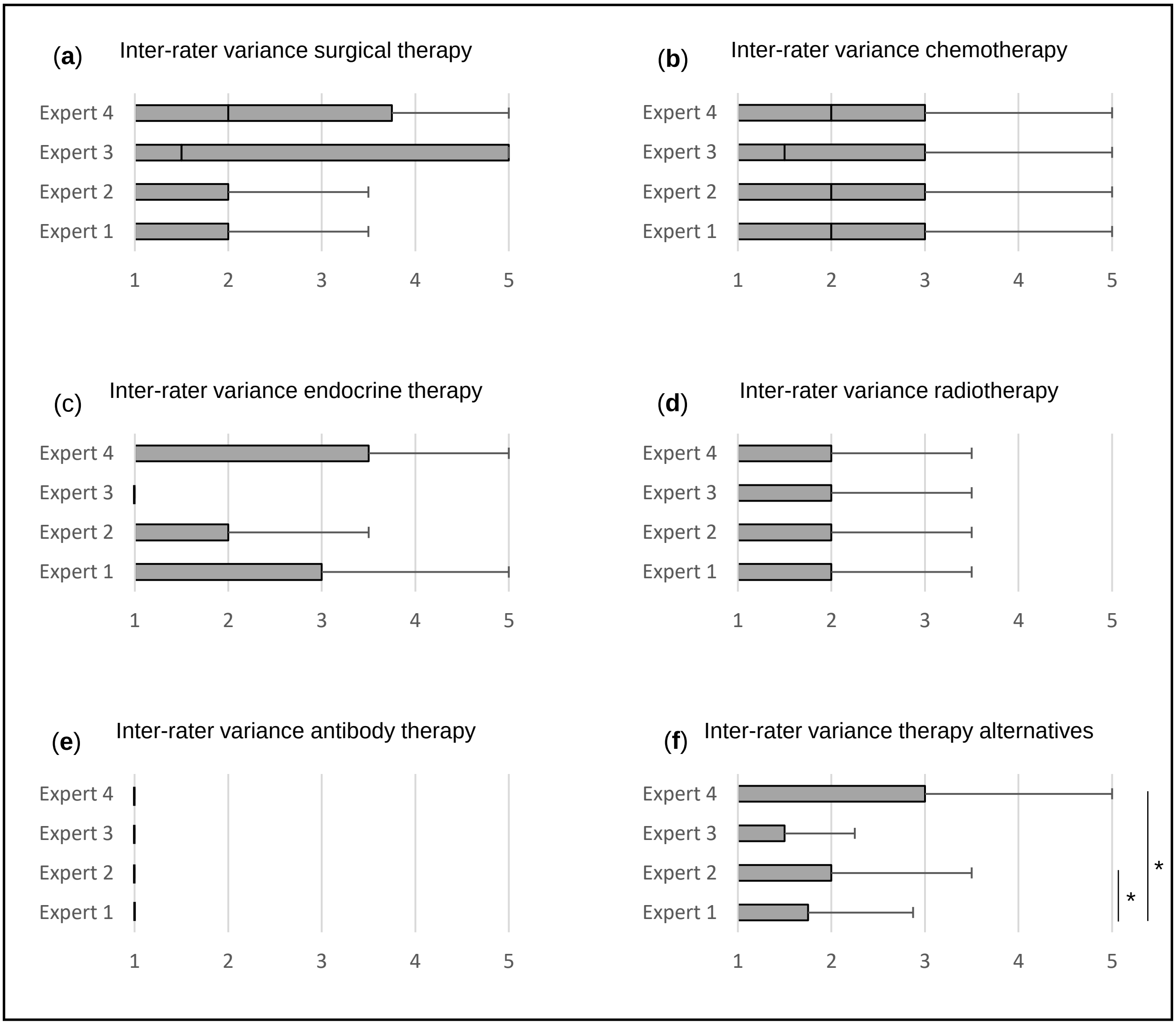

Figure S1:

Analyses of inter-rater variance for each recommendation category. (**a**) Inter-rater variance for ratings of surgical therapy recommendations. (**b**) Inter-rater variance for ratings of chemotherapy. (**c**) Inter-rater variance for ratings of endocrine therapy recommendations. (**d**) Inter-rater variance for ratings of radiotherapy recommendations. (**e**) Inter-rater variance for ratings of antibody therapy recommendations. **(f)** Inter-rater variance for ratings of therapy alternatives. Experts rated the suggestions with the following scale: *correct without limitations* = 1, *correct with minor limitations* = 2, *better alternative available* = 3, *partially wrong* = 4 and *completely wrong* = 5. Boxplots include marked median. *p < 0.05 (Students T-test).
