# Supplementary material for: Quality of ChatGPT-Generated Therapy Recommendations for Breast Cancer Treatment in Gynecology
Source: Curr Oncol. 2024 Jul 1;31(7):3845–54. doi: 10.3390/curroncol31070284 (PMC11275284; doi:10.3390/curroncol31070284)
Supplement: Supplementary file 1 [file curroncol-31-00284-s001.zip › File S1_Patient cases.pdf]

Fall 1

Anamnese:
38-jährig, G2/P2
Vorerkrankungen: Endometriose, Hypothyreose
Voroperationen: Z.n. 1 x Sectio, Z.n. Appendektomie, Z.n. Endometriose-Operation
Medikamente: L-Thyroxin 200 mg/d
Familienanamnese: leer

Aktuelle Diagnose:
Triple negatives Mamma-CA links
cT1b (0,7 cm), cN0, M0
ER 0%, PR 0%, HER2 negativ, Ki-67 40%, G3
Aktuell unter neoadjuvanter Chemotherapie mit Carboplatin/Nab-Paclitaxel, darunter sonographisch Komplettremission
Z.n. humangenetischer Beratung (keine pathogene Mutation für BRCA 1, BRCA 2, CHEK 2-Gen)

Prompt:
Bitte nenne auf Basis der folgenden URL: https://www.leitlinienprogramm-onkologie.de/fileadmin/user_upload/Downloads/Leitlinien/Mammakarzinom_4_0/Version_4.4/LL_Mammakarzinom_Langversion_4.4.pdf, die empfohlene Therapie einer hypothetischen 38-jährigen prämenopausalen Patientin mit einem triple negativen Mammakarzinom links.
Nutze die aktuelle Tumorformel: cT1b (0,7 cm), cN0, M0, ER 0%, PR 0%, HER2 negativ, Ki-67 40%, G3
Es wurde bereits eine neoadjuvante Chemotherapie mit Carboplatin/Nab-Paclitaxel durchgeführt.
Als Vorerkrankungen ist eine Hypothyreose bekannt.
Nenne die hypothetischen Therapieoptionen in einer Tabelle. Nutze als Zeilen Operative Therapie, Chemotherapie, Strahlentherapie, Anti-Her2-Therapie und endokrine Therapie. Nutze als Spalten Empfehlung (ja/nein), das hypothetische Medikament (Wirkstoff und Dosierung), Alternativen und Risiken. Verwende in der Tabelle nur Stichpunkte.
Halte dich so kurz wie möglich, wiederhole nicht den Fall oder die Frage. Sei präzise. Fasse die tabellarische Empfehlung in einem ausgeschriebenen Satz unter der Tabelle zusammen.

Fall 1 – Empfehlung ChatGPT:

Therapieoption	Empfehlung	Medikament (Wirkstoff und Dosierung)	Alternativen	Risiken
Operative Therapie	Ja	Mastektomie oder brusterhaltende Operation	-	Infektionen, Nachblutungen, Narbenbildung
Chemotherapie	Ja	Anthrazyklin-basierte Chemotherapie (z.B. Epirubicin 90 mg/m² + Cyclophosphamid 600 mg/m² alle 3 Wochen i.v.)	-	Knochenmarksuppression, Übelkeit, Haarausfall
Strahlentherapie	Ja	-	-	Hautreaktionen, Müdigkeit
Anti-HER2-Therapie	Nein	-	-	-
Endokrine Therapie	Nein	-	-	-

Die empfohlene Therapie für die hypothetische Patientin mit einem triple negativen Mammakarzinom umfasst eine operative Therapie in Form einer Mastektomie oder brusterhaltenden Operation sowie eine anthrazyklin-basierte Chemotherapie. Eine Strahlentherapie wird empfohlen, während eine Anti-HER2-Therapie und endokrine Therapie nicht empfohlen werden. Risiken und Alternativen sind in der Tabelle aufgeführt.

Für die hypothetische Patientin mit einem triple negativen Mammakarzinom wird eine operative Therapie (Mastektomie oder brusterhaltende Operation), eine anthrazyklin-basierte Chemotherapie und eine Strahlentherapie empfohlen. Eine Anti-HER2-Therapie und endokrine Therapie werden nicht empfohlen.

Translated version

Case 1

Medical history:
Age: 38, G2/P2
Medical history: Endometriosis, Hypothyroidism
Previous surgeries: History of 1 Cesarean section, Appendectomy, Endometriosis surgery
Medications: L-Thyroxine 200 mg/day
Family history: None

Current diagnosis:
Triple-negative breast cancer on the left side
cT1b (0.7 cm), cN0, M0
ER 0%, PR 0%, HER2 negative, Ki-67 40%, G3
Currently undergoing neoadjuvant chemotherapy with Carboplatin/Nab-Paclitaxel, achieving complete remission as per ultrasound
History of genetic counseling (no pathogenic mutation for BRCA 1, BRCA 2, CHEK 2 gene)

Prompt:
Please, based on the following URL: https://www.leitlinienprogramm-onkologie.de/fileadmin/user_upload/Downloads/Leitlinien/Mammakarzinom_4_0/Version_4.4/LL_Mammakarzinom_Langversion_4.4.pdf, provide the recommended therapy for a hypothetical 38-year-old premenopausal patient with triple-negative breast cancer on the left side. Use the current tumor formula: cT1b (0.7 cm), cN0, M0, ER 0%, PR 0%, HER2 negative, Ki-67 40%, G3. Neoadjuvant chemotherapy with Carboplatin/Nab-Paclitaxel has already been administered. Hypothyroidism is a known pre-existing condition.
List the hypothetical treatment options in a table. Use rows for Surgical Therapy, Chemotherapy, Radiotherapy, Anti-HER2 Therapy, and Endocrine Therapy. Use columns for Recommendation (yes/no), hypothetical medication (substance and dosage), Alternatives, and Risks. Use bullet points in the table.
Keep it as concise as possible, avoid repeating the case or the question. Be precise. Summarize the tabular recommendation in a written sentence below the table.

Case 1 – Recommendation ChatGPT

**Therapy options**	**Recommendation**	**Medication (Active agent and dosage)**	**Alternatives**	**Risks**
Surgery	Yes	Mastectomy or breast-conserving surgery	-	Infection, bleeding, scarring
Chemotherapy	Yes	Anthracycline-based chemotherapy (e.g. Epirubicin 90mg/m² + Cyclophosphamide 600mg/m² every 3 weeks i.v.)	-	Myeloid suppression, nausea, hair loss
Radiation	Yes	-	-	Skin reaction, fatigue
Anti-HER2-Therapy	No	-	-	-

Endocrine therapy	No	-	-	-

The recommended therapy for the hypothetical patient with triple-negative breast cancer includes surgical intervention in the form of mastectomy or breast-conserving surgery, along with anthracycline-based chemotherapy. Radiation therapy is recommended, while anti-HER2 therapy and endocrine therapy are not advised. Risks and alternatives are listed in the table.

For the hypothetical patient with triple-negative breast cancer, surgical intervention (mastectomy or breast-conserving surgery), anthracycline-based chemotherapy, and radiation therapy are recommended. Anti-HER2 therapy and endocrine therapy are not recommended.

Fall 2:

Anamnese:
79-jährig, G2/P2
Vorerkrankungen: Arterieller Hypertonus, Hinterwandinfarkt, Diabetes Mellitus, Z.n. Thrombose/Embolie
Medikamente: Metformin, Sartan, Cholesterinsenker, Omeprazol
Familienanamnese: positiv
Voroperationen: Z.n. Hysterektomie, Z.n. Cholezystektomie

Aktuelle Diagnose:
Invasives Mamma-CA (NST) rechts
pT3 (6,0 cm), pN2 (8/12), G3, L1, V0, Pn1, R1 (mamillennah).
ER 100%, PR 80%, HER2-negativ, Ki67 ca. 20%

Operation:
Brusterhaltende Operation rechts und Axilladissektion rechts

Prompt:
Bitte nenne auf Basis der folgenden URL: https://www.leitlinienprogramm-onkologie.de/fileadmin/user_upload/Downloads/Leitlinien/Mammakarzinom_4_0/Version_4.4/LL_Mammakarzinom_Langversion_4.4.pdf, die empfohlene Therapie einer hypothetischen 79-jährigen postmenopausalen Patientin mit einem invasiven Mammakarzinom rechts (NST).
Nutze die aktuelle Tumorformel: pT3 (6,0 cm), pN2 (8/12), G3, L1, V0, Pn1, R1, ER 100%, PR 80%, HER2-negativ, Ki67 ca. 20%
Es wurde bereits eine brusterhaltende Operation rechts und Axilladissektion rechts durchgeführt.
Als Vorerkrankungen sind ein arterieller Hypertonus, ein Diabetes mellitus und ein Herzinfarkt bekannt.
Nenne die hypothetischen Therapieoptionen in einer Tabelle. Nutze als Zeilen Operative Therapie, Chemotherapie, Strahlentherapie, Anti-Her2-Therapie und endokrine Therapie. Nutze als Spalten Empfehlung (ja/nein), das hypothetische Medikament (Wirkstoff und Dosierung), Alternativen und Risiken. Verwende in der Tabelle nur Stichpunkte.
Halte dich so kurz wie möglich, wiederhole nicht den Fall oder die Frage. Sei präzise. Fasse die tabellarische Empfehlung in einem ausgeschriebenen Satz unter der Tabelle zusammen.

Fall 2 – Empfehlung ChatGPT:

Therapieoption	Empfehlung	Medikament (Wirkstoff und Dosierung)	Alternativen	Risiken
Operative Therapie	Ja	Mastektomie	Brusterhaltende Operation	Infektionen, Nachblutungen, Narbenbildung
Chemotherapie	Ja	Anthrazyklin-basierte Chemotherapie (z.B. Epirubicin + Cyclophosphamid)	Taxan-basierte Chemotherapie	Knochenmarksuppressi Übelkeit, Haarausfall
Strahlentherapie	Ja	Strahlentherapie nach brusterhaltender Operation	-	Hautreaktionen, Müdigk
Anti-HER2-Therapie	Nein	-	-	-
Endokrine Therapie	Ja	Aromatasehemmer (z.B. Letrozol)	-	Gelenkbeschwerden, Osteoporose, Hitzewallungen, Stimmungsschwankung

Die empfohlene Therapie für die hypothetische Patientin mit einem invasiven Mammakarzinom rechts umfasst eine operative Therapie in Form einer Mastektomie, eine anthrazyklin-basierte Chemotherapie, eine Strahlentherapie nach brusterhaltender Operation und eine endokrine Therapie mit einem Aromatasehemmer. Eine Anti-HER2-Therapie wird in diesem Fall nicht empfohlen. Risiken und Alternativen sind in der Tabelle aufgeführt.

Für die hypothetische Patientin mit einem invasiven Mammakarzinom rechts wird eine operative Therapie (Mastektomie), eine anthrazyklin-basierte Chemotherapie, eine Strahlentherapie nach brusterhaltender Operation und eine endokrine Therapie mit einem Aromatasehemmer empfohlen. Eine Anti-HER2-Therapie wird nicht empfohlen.

Translated version:

Case 2

Medical history:
79 years old, G2/P2
Pre-existing conditions: Arterial hypertension, posterior wall infarction, diabetes mellitus, history of thrombosis/embolism
Medications: Metformin, sartan, cholesterol-lowering medication, omeprazole
Family history: Positive
Previous surgeries: History of hysterectomy, history of cholecystectomy

Current diagnosis:
Invasive breast cancer (NST) on the right side
pT3 (6.0 cm), pN2 (8/12), G3, L1, V0, Pn1, R1 (close to the nipple).
ER 100%, PR 80%, HER2-negative, Ki67 approximately 20%

Surgery:
Right breast-conserving surgery and right axillary dissection

Prompt:
Please, based on the following URL: https://www.leitlinienprogramm-onkologie.de/fileadmin/user_upload/Downloads/Leitlinien/Mammakarzinom_4_0/Version_4.4/LL_Mammakarzinom_Langversion_4.4.pdf, provide the recommended therapy for a hypothetical 79-year-old postmenopausal patient with invasive breast cancer on the right side (NST). Use the current tumor formula: pT3 (6.0 cm), pN2 (8/12), G3, L1, V0, Pn1, R1, ER 100%, PR 80%, HER2-negative, Ki67 approximately 20%. Breast-conserving surgery and right axillary dissection have already been performed. Pre-existing conditions include arterial hypertension, diabetes mellitus, and a history of heart attack.
List the hypothetical treatment options in a table. Use rows for Surgical Therapy, Chemotherapy, Radiotherapy, Anti-HER2 Therapy, and Endocrine Therapy. Use columns for Recommendation (yes/no), hypothetical medication (substance and dosage), Alternatives, and Risks. Use bullet points in the table.
Keep it as concise as possible, avoid repeating the case or the question. Be precise. Summarize the tabular recommendation in a written sentence below the table.

Case 2 – Recommendation ChatGPT

**Therapy options**	**Recommendation**	**Medication (Active agent and dosage)**	**Alternatives**	**Risks**
Surgery	Yes	Mastectomy	Breast-conserving surgery	Infection, bleeding, scarring
Chemotherapy	Yes	Anthracycline-based chemotherapy (e.g. Epirubicin + Cyclophosphamide)	Taxane-based chemotherapy	Myeloid suppression, nausea, hair loss
Radiation	Yes	Radiation after breast-conserving surgery	-	Skin reaction, fatigue
Anti-HER2-Therapy	No	-	-	-

Endocrine therapy	Yes	Aromatase inhibitor (e.g. letrozole)	-	Joint discomfort, osteoporosis, hot flushes, mood swings

The recommended therapy for the hypothetical patient with invasive breast cancer on the right side includes surgical treatment in the form of a mastectomy, anthracycline-based chemotherapy, radiation therapy following breast-conserving surgery, and endocrine therapy with an aromatase inhibitor. Anti-HER2 therapy is not recommended in this case. Risks and alternatives are listed in the table.

For the hypothetical patient with invasive breast cancer on the right side, surgical treatment (mastectomy), anthracycline-based chemotherapy, radiation therapy following breast-conserving surgery, and endocrine therapy with an aromatase inhibitor are recommended. Anti-HER2 therapy is not recommended.

Fall 3:

Anamnese:
87-jährige Patientin, G4/P2
Vorerkrankungen: Maculadegeneration, Carotisstenose, Z.n. rez. HWI
kein Nikotinabusus
Familienanamnese: leer
Allergien: Amoxicillin, Trihydrat
Medikamenteneinnahme: ASS, Rosuvastatin, Jodesten

Aktuelle Diagnose:
Invasiv lobuläres Mammakarzinom rechts
pT2 (maximal 3,1 cm Durchmesser), pN1a (1/9, 0,7 cm), G2, L0, V0, Pn1, R1 (mamillennah)
Immunphänotyp: ER 100%; PR 60%; HER2-negativ; Ki67 25%

Operation:
Mammasegmentresektion rechts und Sentinellymphonodektomie

Prompt:
Bitte nenne auf Basis der folgenden URL: https://www.leitlinienprogramm-onkologie.de/fileadmin/user_upload/Downloads/Leitlinien/Mammakarzinom_4_0/Version_4.4/LL_Mammakarzinom_Langversion_4.4.pdf, die empfohlene Therapie einer hypothetischen 87-jährigen postmenopausalen Patientin mit einem invasiven lobulären Mammakarzinom rechts.
Nutze die aktuelle Tumorformel: pT2, pN1a (1/9, 0,7 cm), G2, L0, V0, Pn1, R1, ER 100%; PR 60%; HER2-negativ; Ki67 25%
Es wurde bereits eine Mammasegmentresektion rechts und Sentinellymphonodektomie rechts durchgeführt.
Als Vorerkrankungen sind eine Maculadegeneration und eine Carotisstenose bekannt.
Nenne die hypothetischen Therapieoptionen in einer Tabelle. Nutze als Zeilen Operative Therapie, Chemotherapie, Strahlentherapie, Anti-Her2-Therapie und endokrine Therapie. Nutze als Spalten Empfehlung (ja/nein), das hypothetische Medikament (Wirkstoff und Dosierung), Alternativen und Risiken. Verwende in der Tabelle nur Stichpunkte.
Halte dich so kurz wie möglich, wiederhole nicht den Fall oder die Frage. Sei präzise. Fasse die tabellarische Empfehlung in einem ausgeschriebenen Satz unter der Tabelle zusammen.

Fall 3 – Empfehlung ChatGPT:

Therapieoption	Empfehlung	Medikament (Wirkstoff und Dosierung)	Alternativen	Risiken
Operative Therapie	Ja	Mastektomie	Brusterhaltende Operation	Infektionen, Nachblutungen, Narbenbildung
Chemotherapie	Nein	-	-	-
Strahlentherapie	Ja	Strahlentherapie nach brusterhaltender Operation	-	Hautreaktionen, Müdigkeit
Anti-HER2-Therapie	Nein	-	-	-
Endokrine Therapie	Ja	Aromatasehemmer (z.B. Letrozol)	Tamoxifen	Gelenkbeschwerden, Osteoporose, Hitzewallungen, Stimmungsschwankung

Für die hypothetische Patientin mit einem invasiven lobulären Mammakarzinom rechts wird eine operative Therapie in Form einer Mastektomie sowie eine Strahlentherapie nach brusterhaltender Operation empfohlen. Eine Chemotherapie und eine Anti-HER2-Therapie werden in diesem Fall nicht empfohlen. Die endokrine Therapie mit einem Aromatasehemmer (z.B. Letrozol) ist empfohlen, wobei Tamoxifen eine Alternative darstellt. Risiken und Alternativen sind in der Tabelle aufgeführt.

Für die hypothetische Patientin mit einem invasiven lobulären Mammakarzinom rechts wird eine operative Therapie (Mastektomie), eine Strahlentherapie nach brusterhaltender Operation und eine endokrine Therapie mit einem Aromatasehemmer empfohlen. Eine Chemotherapie und eine Anti-HER2-Therapie werden nicht empfohlen.

Translated version:

Case 3

Medical history:
87-year-old patient, G4/P2
Pre-existing conditions: Macular degeneration, carotid stenosis, history of recurrent urinary tract infection
No nicotine abuse
Family history: None
Allergies: Amoxicillin, trihydrate
Medication: Aspirin (ASS), Rosuvastatin, Iodine supplements

Current diagnosis:
Invasive lobular breast cancer on the right side
pT2 (maximum diameter 3.1 cm), pN1a (1/9, 0.7 cm), G2, L0, V0, Pn1, R1 (near areola)
Immunophenotype: ER 100%; PR 60%; HER2-negative; Ki67 25%

Surgery:
Right breast segmental resection and sentinel lymph node dissection

Prompt:
Please, based on the following URL: https://www.leitlinienprogramm-onkologie.de/fileadmin/user_upload/Downloads/Leitlinien/Mammakarzinom_4_0/Version_4.4/LL_Mammakarzinom_Langversion_4.4.pdf, provide the recommended therapy for a hypothetical 87-year-old postmenopausal patient with invasive lobular breast cancer on the right side. Use the current tumor formula: pT2, pN1a (1/9, 0.7 cm), G2, L0, V0, Pn1, R1, ER 100%; PR 60%; HER2-negative; Ki67 25%. Right breast segmental resection and sentinel lymph node dissection have already been performed. Pre-existing conditions include macular degeneration and carotid stenosis.
List the hypothetical treatment options in a table. Use rows for Surgical Therapy, Chemotherapy, Radiotherapy, Anti-HER2 Therapy, and Endocrine Therapy. Use columns for Recommendation (yes/no), hypothetical medication (substance and dosage), Alternatives, and Risks. Use bullet points in the table.
Keep it as concise as possible, avoid repeating the case or the question. Be precise. Summarize the tabular recommendation in a written sentence below the table.

Case 3 – Recommendation ChatGPT

**Therapy options**	**Recommendation**	**Medication (Active agent and dosage)**	**Alternatives**	**Risks**
Surgery	Yes	Mastectomy	Breast-conserving surgery	Infection, bleeding, scarring
Chemotherapy	No	-	-	-
Radiation	Yes	Radiation after breast-conserving surgery	-	Skin reaction, fatigue
Anti-HER2-Therapy	No	-	-	-
Endocrine therapy	Yes	Aromatase inhibitor (e.g. letrozole)	Tamoxifen	Joint discomfort, osteoporosis, hot flushes, mood swings

For the hypothetical patient with invasive lobular breast cancer on the right side, surgical treatment in the form of a mastectomy, as well as radiation therapy following breast-conserving surgery, is recommended. Chemotherapy and anti-HER2 therapy are not recommended in this case. Endocrine therapy with an aromatase inhibitor (e.g., letrozole) is recommended, with tamoxifen being an alternative. Risks and alternatives are listed in the table.

For the hypothetical patient with invasive lobular breast cancer on the right side, surgical treatment (mastectomy), radiation therapy following breast-conserving surgery, and endocrine therapy with an aromatase inhibitor are recommended. Chemotherapy and anti-HER2 therapy are not recommended.

Fall 4:

Anamnese:
68jährige G2/P2
Vor 9 Jahren DCIS, brusterhaltende Therapie und Nachresektion links, anschließend Radiatio
Vor 6 Jahren Mammakarzinom links, pT1b (8 mm), pN0 (0/14, 6 sn); G2, R0, pTis (5 cm)
ER +, PR +, HER2 -, Ki67 15 %
Ablatio Mamma links mit Sentinel-Lymphonodektomie der Axilla links
Therapie mit Tamoxifen für 1 Jahr, anschließend Letrozol für weitere 3 Jahre
Unter Tamoxifen Nekrose des Sprunggelenkes links, unter Letrozol Osteoporose mit Brüchen im Bereich des rechten Fußes

Familienanamnese: Magen-Karzinom
Vorerkrankungen: Arterielle Hypertonie, Diabetes mellitus, Z.n. Thrombose
Von-Willebrand-Syndrom Typ 1

Aktuelle Diagnose:
Herdbefund sonografisch und Tastbefund im Bereich einer Ablationarbe linksseitig
Histologisch Mammakarzinomrezidiv G2, cT1a (5mm), cN0, Mx
ER 100%, PR100%, HER2-negativ, Ki67 ca. 15%
Unauffällige Staging-Untersuchung

Prompt:
Bitte nenne auf Basis der folgenden URL: https://www.leitlinienprogramm-onkologie.de/fileadmin/user_upload/Downloads/Leitlinien/Mammakarzinom_4_0/Version_4.4/LL_Mammakarzinom_Langversion_4.4.pdf, die empfohlene Therapie einer hypothetischen 68-jährigen postmenopausalen Patientin mit einem Mammakarzinomrezidiv links.
Nutze die aktuelle Tumorformel: cT1a (5mm), cN0, Mx, G2, ER 100%, PR100%, HER2-negativ, Ki67 ca. 15%
Berücksichtige die Vorgeschichte der hypothetischen Patientin mit einem Mammakarzinom links vor 6 Jahren bei dem bereits eine Mamma-Ablation links sowie eine Sentinel-Lymphonodektomie der Axilla links durchgeführt wurde.
Als Vorerkrankungen sind eine arterielle Hypertonie und ein Diabetes mellitus bekannt.
Nenne die hypothetischen Therapieoptionen in einer Tabelle. Nutze als Zeilen Operative Therapie, Chemotherapie, Strahlentherapie, Anti-Her2-Therapie und endokrine Therapie. Nutze als Spalten Empfehlung (ja/nein), das hypothetische Medikament (Wirkstoff und Dosierung), Alternativen und Risiken. Verwende in der Tabelle nur Stichpunkte.
Halte dich so kurz wie möglich, wiederhole nicht den Fall oder die Frage. Sei präzise. Fasse die tabellarische Empfehlung in einem ausgeschriebenen Satz unter der Tabelle zusammen.

Fall 4 – Empfehlung ChatGPT:

Therapieoption	Empfehlung	Medikament (Wirkstoff und Dosierung)	Alternativen	Risiken
Operative Therapie	Ja	Mastektomie	Brusterhaltende Operation	Infektionen, Nachblutungen, Narbenbildung
Chemotherapie	Ja	Epirubicin/Cyclophosphamid (EC)	Docetaxel/Cyclophosphamid (TC)	Knochenmarksuppression, Übelkeit, Haarausfall
Strahlentherapie	Ja	Bestrahlung der Brustwand und Axilla	-	Hautreaktionen, Müdigkeit
Anti-HER2-Therapie	Nein	-	-	-
Endokrine Therapie	Ja	Aromatasehemmer (z.B. Letrozol)	Tamoxifen	Gelenkbeschwerden, Osteoporose, Hitzewallungen

Für die hypothetische Patientin mit einem Mammakarzinomrezidiv links wird eine operative Therapie in Form einer Mastektomie, eine Chemotherapie mit Epirubicin/Cyclophosphamid (EC) sowie eine Strahlentherapie der Brustwand und Axilla empfohlen. Eine Anti-HER2-Therapie wird in diesem Fall nicht empfohlen. Die endokrine Therapie mit einem Aromatasehemmer (z.B. Letrozol) ist empfohlen, wobei Tamoxifen eine Alternative darstellt. Risiken und Alternativen sind in der Tabelle aufgeführt.

Für die hypothetische Patientin mit einem Mammakarzinomrezidiv links wird eine operative Therapie (Mastektomie), eine Chemotherapie (Epirubicin/Cyclophosphamid) und eine Strahlentherapie empfohlen. Eine Anti-HER2-Therapie wird nicht empfohlen. Die endokrine Therapie mit einem Aromatasehemmer ist empfohlen.

Translated version:

Case 4

Medical history:
68 years old, G2/P2
9 years ago, ductal carcinoma in situ (DCIS), breast-conserving therapy, and re-resection on the left, followed by radiation
6 years ago, left breast carcinoma, pT1b (8 mm), pN0 (0/14, 6 sentinel nodes); G2, R0, pTis (5 cm)
ER +, PR +, HER2 -, Ki67 15%
Left mastectomy with sentinel lymph node dissection on the left axilla
Therapy with Tamoxifen for 1 year, followed by Letrozole for another 3 years
Necrosis of the left ankle under Tamoxifen, osteoporosis with fractures in the area of the right foot under Letrozole

Family history: Gastric carcinoma
Pre-existing conditions: arterial hypertension, diabetes mellitus, history of thrombosis, von Willebrand syndrome type 1

Current diagnosis:
Sonographically and palpably detected lesion in the area of the left ablation scar
Histologically confirmed recurrence of breast carcinoma G2, cT1a (5 mm), cN0, Mx
ER 100%, PR 100%, HER2-negative, Ki67 approximately 15%
Unremarkable staging examination

Prompt:
Please, based on the following URL: https://www.leitlinienprogramm-onkologie.de/fileadmin/user_upload/Downloads/Leitlinien/Mammakarzinom_4_0/Version_4.4/LL_Mammakarzinom_Langversion_4.4.pdf, provide the recommended therapy for a hypothetical 68-year-old postmenopausal patient with a left-sided recurrence of breast carcinoma. Use the current tumor formula: cT1a (5 mm), cN0, Mx, G2, ER 100%, PR 100%, HER2-negative, Ki67 approximately 15%. Consider the medical history of the hypothetical patient with breast carcinoma on the left side 6 years ago, where a left mastectomy and sentinel lymph node dissection on the left axilla were performed. Pre-existing conditions include arterial hypertension and diabetes mellitus.
List the hypothetical treatment options in a table. Use rows for Surgical Therapy, Chemotherapy, Radiotherapy, Anti-HER2 Therapy, and Endocrine Therapy. Use columns for Recommendation (yes/no), hypothetical medication (substance and dosage), Alternatives, and Risks. Use bullet points in the table.
Keep it as concise as possible, avoid repeating the case or the question. Be precise. Summarize the tabular recommendation in a written sentence below the table.

Case 4 – Recommendation ChatGPT

**Therapy options**	**Recommendation**	**Medication (Active agent and dosage)**	**Alternatives**	**Risks**
Surgery	Yes	Mastectomy	Breast-conserving surgery	Infection, bleeding, scarring
Chemotherapy	Yes	Epirubicin / Cyclophosphamide (EC)	Docetaxel / Cyclophosphamide (TC)	Myeloid suppression, nausea, hair loss

Radiation	Yes	Radiation therapy of the chest wall and axilla	-	Skin reaction, fatigue
Anti-HER2-Therapy	No	-	-	-
Endocrine therapy	Yes	Aromatase inhibitor (e.g. letrozole)	Tamoxifen	Joint discomfort, osteoporosis, hot flushes

For the hypothetical patient with a left-sided breast cancer recurrence, surgical treatment in the form of a mastectomy, chemotherapy with Epirubicin/Cyclophosphamide (EC), and radiation therapy of the chest wall and axilla are recommended. Anti-HER2 therapy is not recommended in this case. Endocrine therapy with an aromatase inhibitor (e.g., Letrozole) is recommended, with Tamoxifen being an alternative. Risks and alternatives are listed in the table.

For the hypothetical patient with a left-sided breast cancer recurrence, surgical treatment (mastectomy), chemotherapy (Epirubicin/Cyclophosphamide), and radiation therapy are recommended. Anti-HER2 therapy is not recommended. Endocrine therapy with an aromatase inhibitor is recommended.

Fall 5:

Anamnese:
64-jährige, postmenopausale, Nulligravida
Vorerkrankungen: Z.n. tiefer Venenthrombose mit Lungenarterienembolie unter Hormonsubstitution (seither keine Hormone mehr), Hypothyreose
Allergie: Pollen
Medikation: L-Thyroxin, Simvastatin, Ginko
Familienanamnese bzgl. Malignomen leer

Aktuelle Diagnose:
Inv. Mammakarzinom (NST) rechts
pT1b (1,0 cm), pTis (1,5 cm), pN0 (0/1 sn), G1, L0, V0, Pn0, R0
ER 100%, PR 50%, HER2-negativ., Ki 67 10%, G1

Operation:
Mammasegmentresektion rechts sowie Sentinellymphonodektomie rechts axillär

Prompt:
Bitte nenne auf Basis der folgenden URL: https://www.leitlinienprogramm-onkologie.de/fileadmin/user_upload/Downloads/Leitlinien/Mammakarzinom_4_0/Version_4.4/LL_Mammakarzinom_Langversion_4.4.pdf, die empfohlene Therapie einer hypothetischen 64-jährigen postmenopausalen Patientin mit einem invasiven Mammakarzinom rechts (NST).
Nutze die aktuelle Tumorformel: pT1b (1,0 cm), pTis (1,5 cm), pN0 (0/1 sn), L0, V0, Pn0, R0, ER 100%, PR 50%, HER2-negativ., Ki 67 10%, G1
Es wurde bereits eine Mammasegmentresektion rechts sowie Sentinellymphonodektomie rechts axillär durchgeführt.
Als Vorerkrankungen sind eine Hypothyreose und eine tiefe Beinvenenthrombose bekannt.
Nenne die hypothetischen Therapieoptionen in einer Tabelle. Nutze als Zeilen Operative Therapie, Chemotherapie, Strahlentherapie, Anti-Her2-Therapie und endokrine Therapie. Nutze als Spalten Empfehlung (ja/nein), das hypothetische Medikament (Wirkstoff und Dosierung), Alternativen und Risiken. Verwende in der Tabelle nur Stichpunkte.
Halte dich so kurz wie möglich, wiederhole nicht den Fall oder die Frage. Sei präzise. Fasse die tabellarische Empfehlung in einem ausgeschriebenen Satz unter der Tabelle zusammen.

Fall 5 – Empfehlung ChatGPT:

Therapieoption	Empfehlung	Medikament (Wirkstoff und Dosierung)	Alternativen	Risiken
Operative Therapie	Ja	Mastektomie oder brusterhaltende Operation mit Bestrahlung	-	Infektionen, Nachblutungen, Narbenbildung
Chemotherapie	Nein	-	-	-
Strahlentherapie	Ja	Bestrahlung der Brustwand bei brusterhaltender Operation	-	Hautreaktionen, Müdigkeit
Anti-HER2-Therapie	Nein	-	-	-
Endokrine Therapie	Ja	Aromatasehemmer (z.B. Anastrozol)	Tamoxifen	Gelenkbeschwerden, Osteoporose, Hitzewallungen

Für die hypothetische Patientin mit einem invasiven Mammakarzinom rechts wird eine operative Therapie in Form einer Mastektomie oder brusterhaltenden Operation mit Bestrahlung empfohlen. Eine Chemotherapie und Anti-HER2-Therapie werden nicht empfohlen. Bei der endokrinen Therapie ist ein Aromatasehemmer wie Anastrozol empfohlen, wobei Tamoxifen eine Alternative darstellt. Risiken und Alternativen sind in der Tabelle aufgeführt.

Für die hypothetische Patientin mit einem invasiven Mammakarzinom rechts (NST) wird eine operative Therapie (Mastektomie oder brusterhaltende Operation mit Bestrahlung) und eine endokrine Therapie mit einem Aromatasehemmer (z.B. Anastrozol) empfohlen. Eine Chemotherapie und Anti-HER2-Therapie sind in diesem Fall nicht empfohlen. Risiken und Alternativen sind in der Tabelle aufgeführt.

Translated version:

Case 5

Medical history:
64-year-old, postmenopausal, nulligravida
Pre-existing conditions: History of deep vein thrombosis with pulmonary artery embolism under hormone replacement therapy (no hormones since then), hypothyroidism
Allergy: Pollen
Medication: L-Thyroxine, Simvastatin, Ginkgo
Family history regarding malignancies: None

Current diagnosis:
Invasive breast cancer (NST) on the right side
pT1b (1.0 cm), pTis (1.5 cm), pN0 (0/1 sn), G1, L0, V0, Pn0, R0
ER 100%, PR 50%, HER2-negative, Ki 67 10%, G1

Surgery:
Right breast segmental resection and right axillary sentinel lymph node dissection

Prompt:
Please, based on the following URL: https://www.leitlinienprogramm-onkologie.de/fileadmin/user_upload/Downloads/Leitlinien/Mammakarzinom_4_0/Version_4.4/LL_Mammakarzinom_Langversion_4.4.pdf, provide the recommended therapy for a hypothetical 64-year-old postmenopausal patient with invasive breast cancer on the right side (NST). Use the current tumor formula: pT1b (1.0 cm), pTis (1.5 cm), pN0 (0/1 sn), L0, V0, Pn0, R0, ER 100%, PR 50%, HER2-negative, Ki 67 10%, G1. Right breast segmental resection and right axillary sentinel lymph node dissection have already been performed. Pre-existing conditions include hypothyroidism and a history of deep vein thrombosis.
List the hypothetical treatment options in a table. Use rows for Surgical Therapy, Chemotherapy, Radiotherapy, Anti-HER2 Therapy, and Endocrine Therapy. Use columns for Recommendation (yes/no), hypothetical medication (substance and dosage), Alternatives, and Risks. Use bullet points in the table.
Keep it as concise as possible, avoid repeating the case or the question. Be precise. Summarize the tabular recommendation in a written sentence below the table.

Case 5 – Recommendation ChatGPT

**Therapy options**	**Recommendation**	**Medication (Active agent and dosage)**	**Alternatives**	**Risks**
Surgery	Yes	Mastectomy or breast-conserving surgery with radiation	-	Infection, bleeding, scarring
Chemotherapy	No	-	-	-
Radiation	Yes	Radiation therapy of the chest wall after breast-conserving surgery	-	Skin reaction, fatigue
Anti-HER2-Therapy	No	-	-	-
Endocrine therapy	Yes	Aromatase inhibitor (e.g. anastrozole)	Tamoxifen	Joint discomfort, osteoporosis, hot flushes

For the hypothetical patient with invasive breast cancer on the right side, surgical treatment in the form of a mastectomy or breast-conserving operation with radiation is recommended. Chemotherapy and anti-HER2 therapy are not recommended. In the case of endocrine therapy, an aromatase inhibitor such as Anastrozole is recommended, with Tamoxifen as an alternative. Risks and alternatives are listed in the table.

For the hypothetical patient with invasive breast cancer on the right side (NST), surgical treatment (mastectomy or breast-conserving operation with radiation) and endocrine therapy with an aromatase inhibitor (e.g., Anastrozole) are recommended. Chemotherapy and anti-HER2 therapy are not recommended in this case. Risks and alternatives are listed in the table.

Fall 6:

Anamnese:
72-jährige, G3/P3
Art. Hypertonie, Hypothyreose
Z.n. Appendektomie
Keine Allergien
Medikation: L-Thyroxin, Antihypertensivum
Familienanamnese leer bzgl. Malignomen

Aktuelle Diagnose:
Invasives Mammakarzinom rechts, G2
Initial: cT2 (sonographisch 22mm, mammographisch 35mm Mikrokalk), cN0 (histologisch unauffälliger Lymphknoten), Mx
Aktuelle Tumorformel:
pT2 (2,1 cm), pTis (1,5 cm), pN0 (0/3 sn), G2, L0, V0, Pn0, R0 (DCIS), R0 (mit Nachresektaten)
ER 100 %, PR 95 %, Her2 neg. (1+), Ki67 ca. 10-15 %

Operation:
Brusterhaltende Therapie sowie Sentinellymphonodektomie

Prompt:
Bitte nenne auf Basis der folgenden URL: https://www.leitlinienprogramm-onkologie.de/fileadmin/user_upload/Downloads/Leitlinien/Mammakarzinom_4_0/Version_4.4/LL_Mammakarzinom_Langversion_4.4.pdf, die empfohlene Therapie einer hypothetischen 72-jährigen postmenopausalen Patientin mit einem invasiven Mammakarzinom rechts.
Nutze die aktuelle Tumorformel: pT2 (2,1cm), pTis (1,5 cm), pN0 (0/3 sn), G2, L0, V0, Pn0, R0 (DCIS), R0 (mit Nachresektaten), ER 100 %, PR 95 %, HER2-negativ, Ki67 ca. 10-15 %
Es wurde bereits eine brusterhaltende Therapie sowie Sentinellymphonodektomie rechts durchgeführt.
Als Vorerkrankungen sind eine arterielle Hypertonie und eine Hypothyreose bekannt.
Nenne die hypothetischen Therapieoptionen in einer Tabelle. Nutze als Zeilen Operative Therapie, Chemotherapie, Strahlentherapie, Anti-Her2-Therapie und endokrine Therapie. Nutze als Spalten Empfehlung (ja/nein), das hypothetische Medikament (Wirkstoff und Dosierung), Alternativen und Risiken. Verwende in der Tabelle nur Stichpunkte.
Halte dich so kurz wie möglich, wiederhole nicht den Fall oder die Frage. Sei präzise. Fasse die tabellarische Empfehlung in einem ausgeschriebenen Satz unter der Tabelle zusammen.

Fall 6 – Empfehlung ChatGPT:

Therapieoption	Empfehlung	Medikament (Wirkstoff und Dosierung)	Alternativen	Risiken
Operative Therapie	Ja	Mastektomie oder brusterhaltende Operation mit Bestrahlung	-	Infektionen, Nachblutungen, Narbenbildung
Chemotherapie	Nein	-	-	-
Strahlentherapie	Ja	Bestrahlung der Brustwand bei brusterhaltender Operation	-	Hautreaktionen, Müdigkeit
Anti-HER2-Therapie	Nein	-	-	-
Endokrine Therapie	Ja	Aromatasehemmer (z.B. Anastrozol)	Tamoxifen	Gelenkbeschwerden, Osteoporose, Hitzewallungen

Für die hypothetische Patientin mit einem invasiven Mammakarzinom rechts wird eine operative Therapie in Form einer Mastektomie oder brusterhaltenden Operation mit Bestrahlung empfohlen. Eine Chemotherapie und Anti-HER2-Therapie werden nicht empfohlen. Bei der endokrinen Therapie ist ein Aromatasehemmer wie Anastrozol empfohlen, wobei Tamoxifen eine Alternative darstellt. Risiken und Alternativen sind in der Tabelle aufgeführt.

Für die hypothetische 72-jährige postmenopausale Patientin mit einem invasiven Mammakarzinom rechts (pT2, pTis, pN0, G2) wird eine operative Therapie in Form einer Mastektomie oder brusterhaltenden Operation mit Bestrahlung sowie eine endokrine Therapie mit einem Aromatasehemmer (z.B. Anastrozol) empfohlen. Eine Chemotherapie und Anti-HER2-Therapie sind in diesem Fall nicht empfohlen. Risiken und Alternativen sind in der Tabelle aufgeführt.

Translated version:

Case 6

Medical history:
72 years old, G3/P3
Arterial hypertension, hypothyroidism
History of appendectomy
No allergies
Medication: L-Thyroxine, antihypertensive
Family history regarding malignancies: None

Current diagnosis:
Invasive breast cancer on the right side, G2
Initial: cT2 (sonographically 22mm, mammographically 35mm microcalcifications), cN0 (histologically inconspicuous lymph nodes), Mx
Current tumor formula:
pT2 (2.1 cm), pTis (1.5 cm), pN0 (0/3 sn), G2, L0, V0, Pn0, R0 (DCIS), R0 (with re-resections)
ER 100%, PR 95%, HER2-negative (1+), Ki67 approximately 10-15%

Surgery:
Breast-conserving therapy and sentinel lymph node dissection

Prompt:
Please, based on the following URL: https://www.leitlinienprogramm-onkologie.de/fileadmin/user_upload/Downloads/Leitlinien/Mammakarzinom_4_0/Version_4.4/LL_Mammakarzinom_Langversion_4.4.pdf, provide the recommended therapy for a hypothetical 72-year-old postmenopausal patient with invasive breast cancer on the right side. Use the current tumor formula: pT2 (2.1 cm), pTis (1.5 cm), pN0 (0/3 sn), G2, L0, V0, Pn0, R0 (DCIS), R0 (with re-resections), ER 100%, PR 95%, HER2-negative, Ki67 approximately 10-15%. Breast-conserving therapy and sentinel lymph node dissection on the right side have already been performed. Pre-existing conditions include arterial hypertension and hypothyroidism.
List the hypothetical treatment options in a table. Use rows for Surgical Therapy, Chemotherapy, Radiotherapy, Anti-HER2 Therapy, and Endocrine Therapy. Use columns for Recommendation (yes/no), hypothetical medication (substance and dosage), Alternatives, and Risks. Use bullet points in the table.
Keep it as concise as possible, avoid repeating the case or the question. Be precise. Summarize the tabular recommendation in a written sentence below the table.

Case 6 – Recommendation ChatGPT

**Therapy options**	**Recommendation**	**Medication (Active agent and dosage)**	**Alternatives**	**Risks**
Surgery	Yes	Mastectomy or breast-conserving surgery with radiation	-	Infection, bleeding, scarring
Chemotherapy	No	-	-	-
Radiation	Yes	Radiation therapy of the chest wall after breast-conserving surgery	-	Skin reaction, fatigue
Anti-HER2-Therapy	No	-	-	-

Endocrine therapy	Yes	Aromatase inhibitor (e.g. anastrozole)	Tamoxifen	Joint discomfort, osteoporosis, hot flushes

For the hypothetical patient with invasive breast cancer on the right side, surgical treatment in the form of a mastectomy or breast-conserving operation with radiation is recommended. Chemotherapy and anti-HER2 therapy are not recommended. In the case of endocrine therapy, an aromatase inhibitor such as Anastrozole is recommended, with Tamoxifen as an alternative. Risks and alternatives are listed in the table.

For the hypothetical 72-year-old postmenopausal patient with invasive breast cancer on the right side (pT2, pTis, pN0, G2), surgical treatment in the form of a mastectomy or breast-conserving operation with radiation, and endocrine therapy with an aromatase inhibitor (e.g., Anastrozole) are recommended. Chemotherapy and anti-HER2 therapy are not recommended in this case. Risks and alternatives are listed in the table.

Fall 7:

Anamnese:
46-jährig, prämenopausal Patientin, G1/P1
Keine internistischen Erkrankungen
Allergie: Pollen
Familienanamnese bzgl. Malignomen leer

Aktuelle Diagnose:
Histologisch gesichertes muzinöses Mammakarzinom links
Initial: cT1c (15mm), cN0, Mx; IHC: ER 90%, PR 30%, Her2 neg., Ki67 5%, G2
Aktuelle Tumorformel:
pT1c (1,3 cm), pN0 (0/3 sn), G2, L0, V0, Pn0, R0
Immunphänotyp: ER +; PR +; HER2-negativ; Ki67 10%

Operation:
Brusterhaltende Therapie sowie Sentinellymphonodektomie

Prompt:
Bitte nenne auf Basis der folgenden URL: https://www.leitlinienprogramm-onkologie.de/fileadmin/user_upload/Downloads/Leitlinien/Mammakarzinom_4_0/Version_4.4/LL_Mammakarzinom_Langversion_4.4.pdf, die empfohlene Therapie einer hypothetischen 46-jährigen prämenopaualen Patientin mit einem muzinösen Mammakarzinom links.
Nutze die aktuelle Tumorformel: pT1c (1,3 cm), pN0 (0/3 sn), G2, L0, V0, Pn0, R0, ER +; PR +; HER2-negativ; Ki67 10%
Es wurde bereits eine brusterhaltende Therapie sowie Sentinellymphonodektomie links durchgeführt.
Es sind keine Vorerkrankungen bekannt.
Nenne die hypothetischen Therapieoptionen in einer Tabelle. Nutze als Zeilen Operative Therapie, Chemotherapie, Strahlentherapie, Anti-Her2-Therapie und endokrine Therapie. Nutze als Spalten Empfehlung (ja/nein), das hypothetische Medikament (Wirkstoff und Dosierung), Alternativen und Risiken. Verwende in der Tabelle nur Stichpunkte.
Halte dich so kurz wie möglich, wiederhole nicht den Fall oder die Frage. Sei präzise. Fasse die tabellarische Empfehlung in einem ausgeschriebenen Satz unter der Tabelle zusammen.

Fall 7 – Empfehlung ChatGPT:

Therapieoption	Empfehlung	Medikament (Wirkstoff und Dosierung)	Alternativen	Risiken
Operative Therapie	Ja	Brusterhaltende Operation mit Bestrahlung	-	Infektionen, Nachblutungen, Narbenbildung
Chemotherapie	Nein	-	-	-
Strahlentherapie	Ja	Bestrahlung der Brustwand bei brusterhaltender Operation	-	Hautreaktionen, Müdigkeit
Anti-HER2-Therapie	Nein	-	-	-
Endokrine Therapie	Ja	Tamoxifen oder Aromatasehemmer (z.B. Anastrozol)	-	Hitzewallungen, Gelenkschmerzen, Osteoporose

Für die hypothetische 46-jährige prämenopausale Patientin mit einem muzinösen Mammakarzinom links (pT1c, pN0, G2) wird eine brusterhaltende Operation mit Bestrahlung empfohlen. Eine Chemotherapie und Anti-HER2-Therapie werden nicht empfohlen. Bei der endokrinen Therapie ist Tamoxifen oder ein Aromatasehemmer wie Anastrozol eine Option. Risiken und Alternativen sind in der Tabelle aufgeführt.

Für die hypothetische 46-jährige prämenopausale Patientin mit einem muzinösen Mammakarzinom links (pT1c, pN0, G2) wird eine brusterhaltende Operation mit Bestrahlung sowie eine endokrine Therapie mit Tamoxifen oder einem Aromatasehemmer empfohlen. Eine Chemotherapie und Anti-HER2-Therapie sind in diesem Fall nicht empfohlen. Risiken und Alternativen sind in der Tabelle aufgeführt.

Translated version:

Case 7

Medical History:
46-year-old, premenopausal patient, G1/P1
No internal medical conditions
Allergy: Pollen
Family history regarding malignancies is empty

Current Diagnosis:
Histologically confirmed mucinous breast carcinoma on the left
Initial: cT1c (15mm), cN0, Mx; IHC: ER 90%, PR 30%, Her2 neg., Ki67 5%, G2
Current Tumor Formula:
pT1c (1.3 cm), pN0 (0/3 sn), G2, L0, V0, Pn0, R0
Immunophenotype: ER +; PR +; HER2-negative; Ki67 10%

Surgery:
Breast-conserving therapy and sentinel lymph node dissection

Prompt:
Please, based on the following URL: https://www.leitlinienprogramm-onkologie.de/fileadmin/user_upload/Downloads/Leitlinien/Mammakarzinom_4_0/Version_4.4/LL_Mammakarzinom_Langversion_4.4.pdf, provide the recommended therapy for a hypothetical 46-year-old premenopausal patient with a mucinous breast carcinoma on the left. Use the current tumor formula: pT1c (1.3 cm), pN0 (0/3 sn), G2, L0, V0, Pn0, R0, ER +; PR +; HER2-negative; Ki67 10%. Breast-conserving therapy and sentinel lymph node dissection on the left have already been performed. No pre-existing conditions are known.
List the hypothetical treatment options in a table. Use rows for Surgical Therapy, Chemotherapy, Radiotherapy, Anti-HER2 Therapy, and Endocrine Therapy. Use columns for Recommendation (yes/no), hypothetical medication (substance and dosage), Alternatives, and Risks. Use bullet points in the table.
Keep it as concise as possible, avoid repeating the case or the question. Be precise. Summarize the tabular recommendation in a written sentence below the table.

Case 7 – Recommendation ChatGPT

**Therapy options**	**Recommendation**	**Medication (Active agent and dosage)**	**Alternatives**	**Risks**
Surgery	Yes	Breast-conserving surgery with radiation	-	Infection, bleeding, scarring
Chemotherapy	No	-	-	-
Radiation	Yes	Radiation therapy of the chest wall after breast-conserving surgery	-	Skin reaction, fatigue
Anti-HER2-Therapy	No	-	-	-
Endocrine therapy	Yes	Tamoxifen or aromatase inhibitor (e.g. anastrozole)	-	Joint discomfort, osteoporosis, hot flushes

For the hypothetical 46-year-old premenopausal patient with a mucinous breast carcinoma on the left (pT1c, pN0, G2), a breast-conserving surgery with radiation is recommended. Chemotherapy and anti-HER2 therapy are not recommended. For endocrine therapy, Tamoxifen or an aromatase inhibitor like Anastrozole is an option. Risks and alternatives are listed in the table.

For the hypothetical 46-year-old premenopausal patient with a mucinous breast carcinoma on the left (pT1c, pN0, G2), a breast-conserving surgery with radiation, as well as endocrine therapy with Tamoxifen or an aromatase inhibitor, is recommended. Chemotherapy and anti-HER2 therapy are not recommended in this case. Risks and alternatives are listed in the table.

Fall 8:

Anamnese:
52-jährig
Multiple endokrine Neoplasie Typ 1 (MEN-Typ 1)
- Z.n. Enukleation eines Insulinoms mit Pankreaslinksresektion
- Z.n. primärem Hyperparathyreoidismus mit Parathyreoidektomie; reimplantierter Nebenschilddrüse
- cMRT Kein Nachweis eines Hypophysenadenoms; intakte adenohypophysäre Partialfunktionen

Z.n. Hysterektomie bei Uterus myomatosus
Z.n. Fibroadenom rechte Brust

Aktuelle Diagnose:
Mammabiopsie (links, mamillenwärts) mit Anteilen eines apokrinen DCIS geringer Differenzierung (G3 nach Van Nuys).

Prompt:
Bitte nenne auf Basis der folgenden URL: https://www.leitlinienprogramm-onkologie.de/fileadmin/user_upload/Downloads/Leitlinien/Mammakarzinom_4_0/Version_4.4/LL_Mammakarzinom_Langversion_4.4.pdf, die empfohlene Therapie einer hypothetischen 52-jährigen postmenopausalen Patientin mit einem apokrinen DCIS links, G3.
Aktuell gibt es keine Tumorformel, der Differenzierung Grad ist G3.
Bisher wurde keine Therapie durchgeführt.
Berücksichtige die Vorgeschichte der hypothetischen Patientin mit multiplen endokrinen Neoplasien Typ 1.
Die hypothetische Patientin wurde bereits hysterektomiert. Sie hat keine weiteren Vorerkrankungen.
Nenne die hypothetischen Therapieoptionen in einer Tabelle. Nutze als Zeilen Operative Therapie, Chemotherapie, Strahlentherapie, Anti-Her2-Therapie und endokrine Therapie. Nutze als Spalten Empfehlung (ja/nein), das hypothetische Medikament (Wirkstoff und Dosierung), Alternativen und Risiken. Verwende in der Tabelle nur Stichpunkte.
Halte dich so kurz wie möglich, wiederhole nicht den Fall oder die Frage. Sei präzise. Fasse die tabellarische Empfehlung in einem ausgeschriebenen Satz unter der Tabelle zusammen.

Fall 8 – Empfehlung ChatGPT:

Therapieoption	Empfehlung	Medikament (Wirkstoff und Dosierung)	Alternativen	Risiken
Operative Therapie	Ja	Brustamputation oder brusterhaltende Operation mit Bestrahlung	-	Infektionen, Nachblutungen, Narbenbildung
Chemotherapie	Nein	-	-	-
Strahlentherapie	Ja	Bestrahlung bei brusterhaltender Operation	-	Hautreaktionen, Müdigkeit
Anti-HER2-Therapie	Nein	-	-	-
Endokrine Therapie	Ja	Tamoxifen oder Aromatasehemmer (z.B. Anastrozol)	-	Hitzewallungen, Gelenkschmerzen, Osteoporose

Für die hypothetische 52-jährige postmenopausale Patientin mit einem apokrinen DCIS links (G3) wird eine operative Therapie in Form einer Brustamputation oder brusterhaltenden Operation mit Bestrahlung empfohlen. Eine Chemotherapie und Anti-HER2-Therapie werden nicht empfohlen. Bei der endokrinen Therapie ist Tamoxifen oder ein Aromatasehemmer wie Anastrozol eine Option. Risiken und Alternativen sind in der Tabelle aufgeführt.

Für die hypothetische 52-jährige postmenopausale Patientin mit einem apokrinen DCIS links (G3) wird eine operative Therapie in Form einer Brustamputation oder brusterhaltenden Operation mit Bestrahlung sowie eine endokrine Therapie mit Tamoxifen oder einem Aromatasehemmer empfohlen. Eine Chemotherapie und Anti-HER2-Therapie sind in diesem Fall nicht empfohlen. Risiken und Alternativen sind in der Tabelle aufgeführt.

Translated version:

Case 8

Medical History:
52 years old
Multiple Endocrine Neoplasia Type 1 (MEN Type 1)
- Post enucleation of an insulinoma with pancreatic left resection
- Post primary hyperparathyroidism with parathyroidectomy; reimplanted parathyroid gland
- cMRI shows no evidence of a pituitary adenoma; intact adenohypophyseal partial functions
Post hysterectomy for uterine myomatosis
Post fibroadenoma in the right breast

Current Diagnosis:
Breast biopsy (left, towards the nipple) with components of low-grade apocrine DCIS (G3 according to Van Nuys).

Prompt:
Please provide, based on the following URL: https://www.leitlinienprogramm-onkologie.de/fileadmin/user_upload/Downloads/Leitlinien/Mammakarzinom_4_0/Version_4.4/LL_Mammakarzinom_Langversion_4.4.pdf, the recommended therapy for a hypothetical 52-year-old postmenopausal patient with apocrine DCIS on the left, G3.
There is currently no tumor formula; the differentiation grade is G3.
No therapy has been performed so far.
Consider the hypothetical patient's history with multiple endocrine neoplasia type 1.
The hypothetical patient has already undergone a hysterectomy. She has no other pre-existing conditions.
List the hypothetical treatment options in a table. Use rows for Surgical Therapy, Chemotherapy, Radiotherapy, Anti-HER2 Therapy, and Endocrine Therapy. Use columns for Recommendation (yes/no), hypothetical medication (substance and dosage), Alternatives, and Risks. Use bullet points in the table.
Keep it as concise as possible, avoid repeating the case or the question. Be precise. Summarize the tabular recommendation in a written sentence below the table.

Case 8 – Recommendation ChatGPT

**Therapy options**	**Recommendation**	**Medication (Active agent and dosage)**	**Alternatives**	**Risks**
Surgery	Yes	Mastectomy or breast-conserving surgery with radiation	-	Infection, bleeding, scarring
Chemotherapy	Nein	-	-	-
Radiation	Yes	Radiation therapy after breast-conserving surgery	-	Skin reaction, fatigue
Anti-HER2-Therapy	Nein	-	-	-
Endocrine therapy	Yes	Tamoxifen or aromatase inhibitor (e.g. anastrozole)	-	Joint discomfort, osteoporosis, hot flushes

For the hypothetical 52-year-old postmenopausal patient with apocrine DCIS on the left (G3), surgical therapy in the form of either mastectomy or breast-conserving surgery with radiation is recommended. Chemotherapy and

anti-HER2 therapy are not advised. In the realm of endocrine therapy, Tamoxifen or an aromatase inhibitor such as Anastrozole is an option. Risks and alternatives are detailed in the table.

For the hypothetical 52-year-old postmenopausal patient with apocrine DCIS on the left (G3), a recommended course of action involves surgical therapy, either through mastectomy or breast-conserving surgery with radiation, along with endocrine therapy using Tamoxifen or an aromatase inhibitor. Chemotherapy and anti-HER2 therapy are not recommended in this case. Risks and alternatives are presented in the table.

Fall 9:

Anamnese:
63-jährige, 1 Para, postmenopausal
Letzte gynäkologische Vorsorge vor Jahren
Z.n. OP bei cerebralem Aneurysma mit Clipping vor 30J
Z.n. Operation an der LWS vor 20J
Bekannte Osteoporose
Keine bekannten internistischen Vorerkrankungen
Nikotinabusus
Allergie: Isocillin, Tramalunverträglichkeit
Familienanamnese: Schwester mit Cervixkarzinom

Aktuelle Diagnose:
ED Exulzerierendes Mammakarzinom links
cT4, cN2 (LK-Metastase links axillär histologisch gesichert), M1 (ossär)
ER 100%, PR 75%, HER2-negativ; Ki67 ca. 30%
Intrinsischer Subtyp (St Gallen 2015): luminal B/A
V.a. Lymphknotenmetastasen axillär rechts sowie infra- und supraclaviculär und cervical
Ossäre Metastasen: Schädelkalotte, vertebral (betont BWK3-5) und costal, linker Humerusschaft, Os sacrum
Nebenbefundlich: ED Hyperthyreose

Prompt:
Bitte nenne auf Basis der folgenden URL: https://www.leitlinienprogramm-onkologie.de/fileadmin/user_upload/Downloads/Leitlinien/Mammakarzinom_4_0/Version_4.4/LL_Mammakarzinom_Langversion_4.4.pdf, die empfohlene Therapie einer hypothetischen 63-jährigen postmenopausalen Patientin mit einem exulzerierenden Mammakarzinom links.
Nutze die aktuelle Tumorformel: cT4, cN2 (Lymphknoten Metastase links axillär), M1 (ossär), ER 100%, PR 75%, HER2-negativ; Ki67 ca. 30%
Es wurde bisher keine Therapie durchgeführt.
Als Vorerkrankungen ist eine Osteoporose bekannt.
Nenne die hypothetischen Therapieoptionen in einer Tabelle. Nutze als Zeilen Operative Therapie, Chemotherapie, Strahlentherapie, Anti-Her2-Therapie und endokrine Therapie. Nutze als Spalten Empfehlung (ja/nein), das hypothetische Medikament (Wirkstoff und Dosierung), Alternativen und Risiken. Verwende in der Tabelle nur Stichpunkte.
Halte dich so kurz wie möglich, wiederhole nicht den Fall oder die Frage. Sei präzise. Fasse die tabellarische Empfehlung in einem ausgeschriebenen Satz unter der Tabelle zusammen.

Fall 9 – Empfehlung ChatGPT:

Therapieoptionen	Empfehlung	Medikament (Wirkstoff und Dosierung)	Alternativen	Risiken
Operative Therapie	Ja	Mastektomie	Brusterhaltende Op	Postoperative Komplikationen
Chemotherapie	Ja	Anthrazyklin- und Taxan-basierte	Keine Chemotherapie	Knochenmarktoxizi[illegible]
		Chemotherapie		Übelkeit, Haarausfa[illegible]
				Müdigkeit
Strahlentherapie	Ja	Bestrahlung der Brustwand und der	Keine Strahlentherapie	Hautreizungen,
		betroffenen Lymphknoten		Fatigue-Syndrom
Anti-HER2-Therapie	Nein	Keine Anti-HER2-Therapie	-	-
Endokrine Therapie	Ja	Aromatasehemmer	Selektiver Estrogenrezeptor-Modulator	Osteoporose, Hitzewallungen

Die empfohlene Therapie umfasst eine operative Therapie in Form einer Mastektomie, eine Chemotherapie mit anthrazyklin- und taxan-basierten Regimen, eine Strahlentherapie der Brustwand und betroffenen Lymphknoten, sowie eine endokrine Therapie mit Aromatasehemmern. Eine Anti-HER2-Therapie ist in diesem Fall nicht empfohlen. Die Risiken und Alternativen sind in der Tabelle aufgeführt.

Translated version:

Case 9

Medical History:
63 years old, 1 parity, postmenopausal
Last gynecological check-up years ago
History of cerebral aneurysm surgery with clipping 30 years ago
History of lumbar spine surgery 20 years ago
Known osteoporosis
No known internal medical conditions
Nicotine abuse
Allergy: Isocillin, intolerance to Tramal
Family history: Sister with cervical carcinoma

Current Diagnosis:
ED Ulcerating breast cancer on the left
cT4, cN2 (histologically confirmed left axillary lymph node metastasis), M1 (osseous)
ER 100%, PR 75%, HER2-negative; Ki67 approximately 30%
Intrinsic subtype (St. Gallen 2015): luminal B/A
Suspicion of lymph node metastases on the right axilla, infraclavicular, supraclavicular, and cervical regions
Osseous metastases: skull vault, vertebral (emphasizing T3-5), costal, left humeral shaft, sacrum
Incidental finding: ED hyperthyroidism

Prompt:
Please provide, based on the following URL: https://www.leitlinienprogramm-onkologie.de/fileadmin/user_upload/Downloads/Leitlinien/Mammakarzinom_4_0/Version_4.4/LL_Mammakarzinom_Langversion_4.4.pdf, the recommended therapy for a hypothetical 63-year-old postmenopausal patient with an ulcerating breast cancer on the left.
Use the current tumor formula: cT4, cN2 (lymph node metastasis left axillary), M1 (osseous), ER 100%, PR 75%, HER2-negative; Ki67 approximately 30%
No therapy has been conducted so far. Osteoporosis is a known pre-existing condition.
List the hypothetical treatment options in a table. Use rows for Surgical Therapy, Chemotherapy, Radiotherapy, Anti-HER2 Therapy, and Endocrine Therapy. Use columns for Recommendation (yes/no), hypothetical medication (substance and dosage), Alternatives, and Risks. Use bullet points in the table.
Keep it as concise as possible, avoid repeating the case or the question. Be precise. Summarize the tabular recommendation in a written sentence below the table.

Case 9 – Recommendation ChatGPT

**Therapy options**	**Recommendation**	**Medication (Active agent and dosage)**	**Alternatives**	**Risks**
Surgery	Yes	Mastectomy	Breast-conserving surgery	Postsurgical complications
Chemotherapy	Yes	Anthracycline- and taxane-based chemotherapy	No chemotherapy	Myeloid toxicity, nausea, hair loss, fatigue
Radiation	Yes	Radiation therapy of the chest wall and relevant lymph nodes	No radiation	Skin reaction, fatigue

Anti-HER2-Therapy	No	No anti-HER2 therapy	-	-
Endocrine therapy	Yes	Aromatase inhibitor	Selective Estrogen Receptor Modulator	Osteoporosis, hot flushes

The recommended therapy includes surgical intervention in the form of a mastectomy, chemotherapy using anthracycline- and taxane-based regimens, radiation therapy targeting the chest wall and affected lymph nodes, as well as endocrine therapy with aromatase inhibitors. Anti-HER2 therapy is not advised in this case. Risks and alternatives are detailed in the table.

Fall 10:

Anamnese:
73-jährig, postmenopausal, 3G/1P
Arterielle Hypertonie, Hypercholesterinämie, Rhythmusstörung, Diabetes Mellitus
Koronare 1-Gefäßerkrankung ohne Progress oder interventionsbedürftige Stenosen (vor 3J)
- mit normaler LV-Funktion
- Z.n. PTCA und DES-Impl. RD1 bei NSTEMI (vor 10J)
- LAD am Abgang des RD1 mittelgradig stenosiert (vor 8J)

Hepatitis B
Z.n. Nephrektomie li. bei Nierenzysten
Z.n. Gastritis vor 4J
Allergien: keine
Rauchen: nein
Medikamente: ASS
Familienanamnese: leer

Aktuelle Diagnose:
ED eines multifokalen Mamma-CA links NST
cT1c, m (10+8+8+3+3mm), cN0 (histologisch kein LK-Gewebe in Stanze, CT unauffällig), MX, G3
Immunphänotyp: ER 100%, PR0%, HER2-positiv, Ki67 fokal bis ca. 45%
Zwei Herde 0,2 x 0,2 x 0,3 cm und 0,3 x 0,2 x 0,2 cm
Zwei Herde 0,8 x 0,8 x 0,5 cm und 0,8 x 1,1 x 0.8 cm
Ein Herd 0,7 x 1,0 x 0,7 cm
- 3 Herde stanzbioptisch gesichert und Clip-markiert
- LK Biopsie links erfolgt: kein LK Gewebe erfasst

Prompt:
Bitte nenne auf Basis der folgenden URL: https://www.leitlinienprogramm-onkologie.de/fileadmin/user_upload/Downloads/Leitlinien/Mammakarzinom_4_0/Version_4.4/LL_Mammakarzinom_Langversion_4.4.pdf, die empfohlene Therapie einer hypothetischen 73-jährigen postmenopausalen Patientin mit einem multifokalen Mammakarzinom links (NST) mit 5 Herden.
Nutze die aktuelle Tumorformel: cT1c, cN0 (histologisch kein LK-Gewebe in Stanze, CT unauffällig), MX, G3, ER 100%, PR0%, HER2-positiv, Ki67 fokal bis ca. 45%
Bisher wurde keine Therapie durchgeführt.
Berücksichtige die Vorgeschichte der hypothetischen Patientin mit einem Myokardinfarkt und einer Nephrektomie links.
Als Vorerkrankungen sind eine Hepatitis B, eine arterielle Hypertonie, Hypercholesterinämie, und ein Diabetes Mellitus bekannt.
Nenne die hypothetischen Therapieoptionen in einer Tabelle. Nutze als Zeilen Operative Therapie, Chemotherapie, Strahlentherapie, Anti-Her2-Therapie und endokrine Therapie. Nutze als Spalten Empfehlung (ja/nein), das hypothetische Medikament (Wirkstoff und Dosierung), Alternativen und Risiken. Verwende in der Tabelle nur Stichpunkte.
Halte dich so kurz wie möglich, wiederhole nicht den Fall oder die Frage. Sei präzise. Fasse die tabellarische Empfehlung in einem ausgeschriebenen Satz unter der Tabelle zusammen.

Fall 10 – Empfehlung ChatGPT:

Therapieoptionen	Empfehlung	Medikament (Wirkstoff und Dosierung)	Alternativen	Risiken
Operative Therapie	Ja	Mastektomie	Brusterhaltende Op	Postoperative Komplikationen
Chemotherapie	Ja	Anthrazyklin- und Taxan-basierte	Keine Chemotherapie	Knochenmarktoxizität,
		Chemotherapie		Übelkeit, Haarausfall,
				Müdigkeit
Strahlentherapie	Ja	Bestrahlung der Brustwand und der	Keine Strahlentherapie	Hautreizungen,
		betroffenen Lymphknoten		Fatigue-Syndrom
Anti-HER2-Therapie	Ja	Trastuzumab + Pertuzumab	Trastuzumab allein	Kardiotoxizität,
		(Herceptin + Perjeta)		Infusionsreaktionen
Endokrine Therapie	Nein	Keine endokrine Therapie	-	-

Die empfohlene Therapie umfasst eine operative Therapie in Form einer Mastektomie, eine Chemotherapie mit anthrazyklin- und taxan-basierten Regimen, eine Strahlentherapie der Brustwand und betroffenen Lymphknoten, sowie eine Anti-HER2-Therapie mit Trastuzumab und Pertuzumab. Eine endokrine Therapie ist in diesem Fall nicht empfohlen. Die Risiken und Alternativen sind in der Tabelle aufgeführt.

Die empfohlene Therapie für die 73-jährige postmenopausale Patientin mit einem multifokalen Mammakarzinom links umfasst eine Mastektomie, eine Chemotherapie, eine Strahlentherapie und eine Anti-HER2-Therapie. Eine endokrine Therapie ist nicht empfohlen. Es wurden Risiken und Alternativen berücksichtigt.

Translated version:

Case 10

Medical History:
73 years old, postmenopausal, 3 pregnancies/1 live birth
Arterial hypertension, hypercholesterolemia, arrhythmia, diabetes mellitus
Coronary one-vessel disease without progression or intervention-requiring stenoses (3 years ago)
- With normal left ventricular function
- Post PTCA and DES implantation in the RD1 for NSTEMI (10 years ago)
- LAD moderately stenosed at the origin of RD1 (8 years ago)

Hepatitis B
Post left nephrectomy for renal cysts
History of gastritis 4 years ago
Allergies: None
Smoking: No
Medications: Aspirin (ASS)
Family history: Empty

Current Diagnosis:
Ulcerating ductal carcinoma in situ (DCIS) in the left breast with multiple foci
cT1c, m (10+8+8+3+3mm), cN0 (no lymph node tissue in punch biopsy, CT unremarkable), MX, G3
Immunophenotype: ER 100%, PR 0%, HER2-positive, Ki67 focally up to approximately 45%
Two foci: 0.2 x 0.2 x 0.3 cm and 0.3 x 0.2 x 0.2 cm
Two foci: 0.8 x 0.8 x 0.5 cm and 0.8 x 1.1 x 0.8 cm
One focus: 0.7 x 1.0 x 0.7 cm
- 3 foci confirmed by core needle biopsy and marked with clips
- Left lymph node biopsy performed: no lymph node tissue detected

Prompt:
Please provide, based on the following URL: https://www.leitlinienprogramm-onkologie.de/fileadmin/user_upload/Downloads/Leitlinien/Mammakarzinom_4_0/Version_4.4/LL_Mammakarzinom_Langversion_4.4.pdf, the recommended therapy for a hypothetical 73-year-old postmenopausal patient with a multifocal ductal carcinoma in situ (DCIS) in the left breast with 5 foci.
Use the current tumor formula: cT1c, cN0 (no lymph node tissue in punch biopsy, CT unremarkable), MX, G3, ER 100%, PR 0%, HER2-positive, Ki67 focally up to approximately 45%
No therapy has been conducted so far. Consider the hypothetical patient's history of myocardial infarction and left nephrectomy.
Pre-existing conditions include Hepatitis B, arterial hypertension, hypercholesterolemia, and diabetes mellitus.
List the hypothetical treatment options in a table. Use rows for Surgical Therapy, Chemotherapy, Radiotherapy, Anti-HER2 Therapy, and Endocrine Therapy. Use columns for Recommendation (yes/no), hypothetical medication (substance and dosage), Alternatives, and Risks. Use bullet points in the table.
Keep it as concise as possible, avoid repeating the case or the question. Be precise. Summarize the tabular recommendation in a written sentence below the table.

Case 10 – Recommendation ChatGPT

**Therapy options**	**Recommendation**	**Medication (Active agent and dosage)**	**Alternatives**	**Risks**
Surgery	Yes	Mastectomy	Breast-conserving surgery	Postsurgical complications

Chemotherapy	Yes	Anthracycline- and taxane-based chemotherapy	No chemotherapy	Myeloid toxicity, nausea, hair loss, fatigue
Radiation	Yes	Radiation therapy of the chest wall and relevant lymph nodes	No radiation	Skin reaction, fatigue
Anti-HER2-Therapy	Yes	Trastuzumab and pertuzumab (Herceptin and perjeta)	Trastuzumab monotherapy	Cardiac toxicity, reaction to infusion
Endocrine therapy	No	No endocrine therapy	-	-

The recommended therapy includes surgical intervention in the form of a mastectomy, chemotherapy using anthracycline- and taxane-based regimens, radiation therapy targeting the chest wall and affected lymph nodes, as well as anti-HER2 therapy with Trastuzumab and Pertuzumab. Endocrine therapy is not advised in this case. Risks and alternatives are detailed in the table.

The recommended therapy for the 73-year-old postmenopausal patient with multifocal breast cancer on the left involves mastectomy, chemotherapy, radiation therapy, and anti-HER2 therapy. Endocrine therapy is not recommended. Risks and alternatives have been taken into account.

Fall 11:

Anamnese:
51-jährig, perimenopausal
Art. Hypertonie
Z.n. Hysterektomie (Querlapartomie) bei Myomen (vor 15J)
Z.n. Ovarialzystenenukleation
Familienanamnese bzgl. Malignomen leer
Hormonersatztherapie mit Livial bei Wechseljahresbeschwerden

Aktuelle Diagnose:
Mammakarzinom NST links bei 2 Uhr mit intratumoralen Nekrosen, LK-Metastasierung
Mind. cT2 (sonographisch 32mm HB, Tastbefund jedoch 7cm), cN+ (4 suspekte Lymphknoten), Mx
Immunphänotyp: ER 0%; PR 0%; HER2-negativ; Ki67 ca. 90%.

Prompt:
Bitte nenne auf Basis der folgenden URL: https://www.leitlinienprogramm-onkologie.de/fileadmin/user_upload/Downloads/Leitlinien/Mammakarzinom_4_0/Version_4.4/LL_Mammakarzinom_Langversion_4.4.pdf, die empfohlene Therapie einer hypothetischen 51-jährigen perimenopausalen Patientin mit einem Mammakarzinom links (NST).
Nutze die aktuelle Tumorformel: cT2 (sonographisch 32mm Herdbefund, Tastbefund jedoch 7cm), cN+ (4 suspekte Lymphknoten), Mx, ER 0%; PR 0%; HER2-negativ; Ki67 ca. 90%.
Es wurde bisher keine Therapie durchgeführt.
Berücksichtige die Vorgeschichte der hypothetischen Patientin mit einer Hormonersatztherapie bei Wechseljahresbeschwerden.
Als Vorerkrankungen ist eine arterielle Hypertonie bekannt.
Nenne die hypothetischen Therapieoptionen in einer Tabelle. Nutze als Zeilen Operative Therapie, Chemotherapie, Strahlentherapie, Anti-Her2-Therapie und endokrine Therapie. Nutze als Spalten Empfehlung (ja/nein), das hypothetische Medikament (Wirkstoff und Dosierung), Alternativen und Risiken. Verwende in der Tabelle nur Stichpunkte.
Halte dich so kurz wie möglich, wiederhole nicht den Fall oder die Frage. Sei präzise. Fasse die tabellarische Empfehlung in einem ausgeschriebenen Satz unter der Tabelle zusammen.

Fall 11 – Empfehlung ChatGPT:

Therapieoptionen	Empfehlung	Medikament (Wirkstoff und Dosierung)	Alternativen	Risiken
Operative Therapie	Ja	Mastektomie oder brusterhaltende	-	Wundheilungsstörungen
		Operation		Infektionen
Chemotherapie	Ja	Anthrazyklin- und Taxan-basierte	Keine Chemotherapie	Knochenmarktoxizität,
		Chemotherapie		Übelkeit, Haarausfall,
				Müdigkeit
Strahlentherapie	Ja	Bestrahlung der Brustwand und der	Keine Strahlentherapie	Hautreizungen,
		betroffenen Lymphknoten		Fatigue-Syndrom
Anti-HER2-Therapie	Nein	Keine Anti-HER2-Therapie	-	-
Endokrine Therapie	Nein	Keine endokrine Therapie	-	-

Die empfohlene Therapie umfasst eine operative Therapie in Form einer Mastektomie oder brusterhaltenden Operation, eine Chemotherapie mit anthrazyklin- und taxan-basierten Regimen und eine Strahlentherapie der Brustwand und betroffenen Lymphknoten. Eine Anti-HER2-Therapie ist in diesem Fall nicht empfohlen, und es wird keine endokrine Therapie empfohlen. Die Risiken und Alternativen sind in der Tabelle aufgeführt.

Die empfohlene Therapie für die 51-jährige perimenopausale Patientin mit einem Mammakarzinom links umfasst eine operative Therapie (Mastektomie oder brusterhaltende Operation), eine Chemotherapie und eine Strahlentherapie. Es wird keine Anti-HER2-Therapie oder endokrine Therapie empfohlen. Es wurden Risiken und Alternativen berücksichtigt.

Translated version:

Case 11

Medical History:
51 years old, perimenopausal
Arterial hypertension
History of hysterectomy (transverse laparotomy) for fibroids (15 years ago)
History of ovarian cyst enucleation
Family history regarding malignancies is empty
Hormone replacement therapy with Livial for menopausal symptoms

Current Diagnosis:
Invasive ductal carcinoma (NST) in the left breast at 2 o'clock with intratumoral necrosis, lymph node metastasis
At least cT2 (sonographically 32mm lesion, clinical examination suggests 7cm), cN+ (4 suspicious lymph nodes), Mx
Immunophenotype: ER 0%; PR 0%; HER2-negative; Ki67 approximately 90%.

Prompt:
Please provide, based on the following URL: https://www.leitlinienprogramm-onkologie.de/fileadmin/user_upload/Downloads/Leitlinien/Mammakarzinom_4_0/Version_4.4/LL_Mammakarzinom_Langversion_4.4.pdf, the recommended therapy for a hypothetical 51-year-old perimenopausal patient with invasive ductal carcinoma (NST) in the left breast.
Use the current tumor formula: cT2 (sonographically 32mm lesion, clinical examination suggests 7cm), cN+ (4 suspicious lymph nodes), Mx, ER 0%; PR 0%; HER2-negative; Ki67 approximately 90%.
No therapy has been conducted so far. Consider the hypothetical patient's history of hormone replacement therapy for menopausal symptoms.
Arterial hypertension is a known pre-existing condition.
List the hypothetical treatment options in a table. Use rows for Surgical Therapy, Chemotherapy, Radiotherapy, Anti-HER2 Therapy, and Endocrine Therapy. Use columns for Recommendation (yes/no), hypothetical medication (substance and dosage), Alternatives, and Risks. Use bullet points in the table.
Keep it as concise as possible, avoid repeating the case or the question. Be precise. Summarize the tabular recommendation in a written sentence below the table.

Case 11 – Recommendation ChatGPT

Therapy options	Recommendation	Medication (Active agent and dosage)	Alternatives	Risks
Surgery	Yes	Mastectomy or breast-conserving surgery	-	Impaired wound healing, infection
Chemotherapy	Yes	Anthracycline- and taxane-based chemotherapy	No chemotherapy	Myeloid toxicity, nausea, hair loss, fatigue
Radiation	Yes	Radiation therapy of the chest wall and relevant lymph nodes	No radiation	Skin reaction, fatigue
Anti-HER2-Therapy	No	No anti-HER2 therapy	-	-

Endocrine therapy	No	No endocrine therapy	-	-

The recommended therapy includes surgical intervention in the form of either mastectomy or breast-conserving surgery, chemotherapy using anthracycline- and taxane-based regimens, and radiation therapy targeting the chest wall and affected lymph nodes. Anti-HER2 therapy is not advised in this case, and no endocrine therapy is recommended. Risks and alternatives are detailed in the table.

The recommended therapy for the 51-year-old perimenopausal patient with left breast cancer involves surgical intervention (mastectomy or breast-conserving surgery), chemotherapy, and radiation therapy. Anti-HER2 therapy or endocrine therapy is not recommended. Risks and alternatives have been taken into account.

Fall 12:

Anamnese:
53-jährige, G1/P1, postmenopausal
Internistisch gesund
Allergien: keine
Medikamenteneinnahme: keine
Nikotinabusus: nein
Familienanamnese: unbekannt

Aktuelle Diagnose:
ED eines invasiven Mammakarzinom NST rechts
Initial: cT1c, cN0, cMx, ER 100%, PR 100%, HER2-negativ, Ki67 ca. 15%
Aktuell: pT1c (maximal 1,1 cm Durchmesser), pN1a (1/2 sn, max. 0,9 cm Durchmesser, fokal kapselüberschreitend), G2, L0, V0, Pn0, R0, lokal.
Immunhistochemie: ER 100%, PR 100%, HER2-negativ, Ki67 ca. 15%

Operation vom:
Brust-erhaltende Therapie sowie Sentinellymphonodektomie rechts

Prompt:
Bitte nenne auf Basis der folgenden URL: https://www.leitlinienprogramm-onkologie.de/fileadmin/user_upload/Downloads/Leitlinien/Mammakarzinom_4_0/Version_4.4/LL_Mammakarzinom_Langversion_4.4.pdf, die empfohlene Therapie einer hypothetischen 53-jährigen postmenopausalen Patientin mit einem invasiven Mammakarzinom rechts (NST).
Nutze die aktuelle Tumorformel: pT1c (maximal 1,1 cm Durchmesser), pN1a (1/2 sn, max. 0,9 cm Durchmesser, fokal kapselüberschreitend), G2, L0, V0, Pn0, R0, ER 100%, PR 100%, HER2-negativ, Ki67 ca. 15%
Es wurde bereits eine brusterhaltende Therapie sowie Sentinellymphonodektomie rechts durchgeführt.
Es sind keine Vorerkrankungen bekannt.
Nenne die hypothetischen Therapieoptionen in einer Tabelle. Nutze als Zeilen Operative Therapie, Chemotherapie, Strahlentherapie, Anti-Her2-Therapie und endokrine Therapie. Nutze als Spalten Empfehlung (ja/nein), das hypothetische Medikament (Wirkstoff und Dosierung), Alternativen und Risiken. Verwende in der Tabelle nur Stichpunkte.
Halte dich so kurz wie möglich, wiederhole nicht den Fall oder die Frage. Sei präzise. Fasse die tabellarische Empfehlung in einem ausgeschriebenen Satz unter der Tabelle zusammen.

Fall 12 – Empfehlung ChatGPT:

Therapieoptionen	Empfehlung	Medikament (Wirkstoff und Dosierung)	Alternativen	Risiken
Operative Therapie	Ja	Brusterhaltende Operation oder	Mastektomie	Infektionen, Blutungen,
		Mastektomie		Serombildung, Narbenbildung
Chemotherapie	Ja	Anthrazyklin- und Taxan-basierte	-	Knochenmarktoxizität,
		Chemotherapie		Übelkeit, Haarausfall,
				Müdigkeit
Strahlentherapie	Ja	Strahlentherapie der Brustwand	-	Hautreizungen, Fatigue-Syndrom
Anti-HER2-Therapie	Nein	Keine Anti-HER2-Therapie	-	-
Endokrine Therapie	Nein	Keine endokrine Therapie	-	-

Die empfohlene Therapie umfasst eine brusterhaltende Operation oder Mastektomie als operative Therapie, eine Anthrazyklin- und Taxan-basierte Chemotherapie und eine Strahlentherapie der Brustwand. Eine Anti-HER2-Therapie oder endokrine Therapie wird in diesem Fall nicht empfohlen. Die Risiken und Alternativen sind in der Tabelle aufgeführt.

Die empfohlene Therapie für die 53-jährige postmenopausale Patientin mit einem invasiven Mammakarzinom rechts umfasst eine brusterhaltende Operation oder Mastektomie, eine Chemotherapie und eine Strahlentherapie der Brustwand. Es wird keine Anti-HER2-Therapie oder endokrine Therapie empfohlen. Risiken und Alternativen wurden berücksichtigt.

Translated version:

Case 12

Medical History:
53 years old, G1/P1, postmenopausal
Internally healthy
Allergies: None
Medication intake: None
Nicotine use: No
Family history: Unknown

Current Diagnosis:
Invasive ductal carcinoma (NST) in the right breast
Initial: cT1c, cN0, cMx, ER 100%, PR 100%, HER2-negative, Ki67 approximately 15%
Current: pT1c (maximal diameter 1.1 cm), pN1a (1/2 sn, maximum diameter 0.9 cm, focal capsular invasion), G2, L0, V0, Pn0, R0, local.
Immunohistochemistry: ER 100%, PR 100%, HER2-negative, Ki67 approximately 15%

Surgery:
Breast-conserving therapy and right sentinel lymph node dissection

Prompt:
Please provide, based on the following URL: https://www.leitlinienprogramm-onkologie.de/fileadmin/user_upload/Downloads/Leitlinien/Mammakarzinom_4_0/Version_4.4/LL_Mammakarzinom_Langversion_4.4.pdf, the recommended therapy for a hypothetical 53-year-old postmenopausal patient with invasive ductal carcinoma in the right breast (NST).
Use the current tumor formula: pT1c (maximal diameter 1.1 cm), pN1a (1/2 sn, maximum diameter 0.9 cm, focal capsular invasion), G2, L0, V0, Pn0, R0, ER 100%, PR 100%, HER2-negative, Ki67 approximately 15%
Breast-conserving therapy and right sentinel lymph node dissection have already been performed.
No pre-existing conditions are known.
List the hypothetical treatment options in a table. Use rows for Surgical Therapy, Chemotherapy, Radiotherapy, Anti-HER2 Therapy, and Endocrine Therapy. Use columns for Recommendation (yes/no), hypothetical medication (substance and dosage), Alternatives, and Risks. Use bullet points in the table.
Keep it as concise as possible, avoid repeating the case or the question. Be precise. Summarize the tabular recommendation in a written sentence below the table.

Case 12 – Recommendation ChatGPT

Therapy options	Recommendation	Medication (Active agent and dosage)	Alternatives	Risks
Surgery	Yes	Mastectomy or breast-conserving surgery	Mastectomy	Infection, bleeding, scarring, seroma formation
Chemotherapy	Yes	Anthracycline- and taxane-based chemotherapy	-	Myeloid toxicity, nausea, hair loss, fatigue
Radiation	Yes	Radiation therapy of the chest wall	-	Skin reaction, fatigue
Anti-HER2-Therapy	No	No anti-HER2 therapy	-	-

Endocrine therapy	No	No endocrine therapy	-	-

The recommended therapy includes breast-conserving surgery or mastectomy as the surgical intervention, anthracycline- and taxane-based chemotherapy, and radiation therapy targeting the chest wall. Anti-HER2 therapy or endocrine therapy is not advised in this case. Risks and alternatives are detailed in the table.

The recommended therapy for the 53-year-old postmenopausal patient with invasive ductal carcinoma in the right breast involves breast-conserving surgery or mastectomy, chemotherapy, and radiation therapy targeting the chest wall. Anti-HER2 therapy or endocrine therapy is not recommended. Risks and alternatives have been taken into account.

Fall 13:

Anamnese:
32-jährige, G0/P0, prämenopausal
Z.n. Mammareduktionsplastik bds. Vor 13J
Keine bekannten Vorerkrankungen oder Allergien
Familienanamnese: Mutter Cervix-Ca, Opa Bronchial-Ca
Mirena-Spirale
Humangenetische Untersuchung initiiert

Aktuelle Diagnose:
ED eines invasiven Mammakarzinoms (NST) links
cT3 (MRT Ausdehnung von 3,8 x 5,8 x 6,9 cm, sonographisch mind. 2,9cm + V.a. Satellitenherde), cN0, M0 ER 100%, PR 100%, HER2-negativ, Ki67 10%, G2
Anbei: DCIS, G2

Staging:
CT-Thorax/Abdomen: Kein Hinweis auf Malignome, Metastasen oder Lymphome, lediglich links axillär unspezifisch betonter Lymphknotenbesatz.

Prompt:
Bitte nenne auf Basis der folgenden URL: https://www.leitlinienprogramm-onkologie.de/fileadmin/user_upload/Downloads/Leitlinien/Mammakarzinom_4_0/Version_4.4/LL_Mammakarzinom_Langversion_4.4.pdf, die empfohlene Therapie einer hypothetischen 32-jährigen prämenopausalen Patientin mit einem invasiven Mammakarzinom links (NST).
Nutze die aktuelle Tumorformel: cT3 (Verdacht auf Satellitenherde), cN0, M0 ER 100%, PR 100%, HER2-negativ, Ki67 10%, G2
Es wurde bisher keine Therapie durchgeführt.
Es sind keine Vorerkrankungen bekannt.
Nenne die hypothetischen Therapieoptionen in einer Tabelle. Nutze als Zeilen Operative Therapie, Chemotherapie, Strahlentherapie, Anti-Her2-Therapie und endokrine Therapie. Nutze als Spalten Empfehlung (ja/nein), das hypothetische Medikament (Wirkstoff und Dosierung), Alternativen und Risiken. Verwende in der Tabelle nur Stichpunkte.
Halte dich so kurz wie möglich, wiederhole nicht den Fall oder die Frage. Sei präzise. Fasse die tabellarische Empfehlung in einem ausgeschriebenen Satz unter der Tabelle zusammen.

Fall 13 – Empfehlung ChatGPT:

Therapieoptionen	Empfehlung	Medikament (Wirkstoff und Dosierung)	Alternativen	Risiken
Operative Therapie	Ja	Brusterhaltende Operation oder	Mastektomie	Infektionen, Blutungen, Narbenbildung
		Mastektomie		Serombildung
Chemotherapie	Ja	Anthrazyklin- und Taxan-basierte	-	Knochenmarktoxizität, Übelkeit,
		Chemotherapie		Haarausfall, Müdigkeit
Strahlentherapie	Ja	Strahlentherapie der Brustwand	-	Hautreizungen, Fatigue-Syndrom
Anti-HER2-Therapie	Nein	Keine Anti-HER2-Therapie	-	-
Endokrine Therapie	Nein	Keine endokrine Therapie	-	-

Die empfohlene Therapie umfasst eine brusterhaltende Operation oder Mastektomie als operative Therapie, eine Anthrazyklin- und Taxan-basierte Chemotherapie und eine Strahlentherapie der Brustwand. Eine Anti-HER2-Therapie oder endokrine Therapie wird in diesem Fall nicht empfohlen. Die Risiken und Alternativen sind in der Tabelle aufgeführt.

Die empfohlene Therapie für die 32-jährige prämenopausale Patientin mit einem invasiven Mammakarzinom links umfasst eine brusterhaltende Operation oder Mastektomie, eine Chemotherapie und eine Strahlentherapie der Brustwand. Es wird keine Anti-HER2-Therapie oder endokrine Therapie empfohlen. Risiken und Alternativen wurden berücksichtigt.

Translated version:

Case 13

Medical History:
32 years old, G0/P0, premenopausal
History of bilateral breast reduction surgery 13 years ago
No known pre-existing conditions or allergies
Family history: Mother with cervical cancer, Grandfather with bronchial cancer
Mirena intrauterine device
Initiated human genetic testing

Current Diagnosis:
Invasive ductal carcinoma (NST) in the left breast
cT3 (MRI dimensions of 3.8 x 5.8 x 6.9 cm, sonographically at least 2.9 cm + suspicion of satellite lesions), cN0, M0 ER 100%, PR 100%, HER2-negative, Ki67 10%, G2
Additional finding: DCIS, G2

Staging:
CT Thorax/Abdomen: No evidence of malignancies, metastases, or lymphomas; only unspecific accentuated lymph nodes on the left axilla.

Prompt:
Please provide, based on the following URL: https://www.leitlinienprogramm-onkologie.de/fileadmin/user_upload/Downloads/Leitlinien/Mammakarzinom_4_0/Version_4.4/LL_Mammakarzinom_Langversion_4.4.pdf, the recommended therapy for a hypothetical 32-year-old premenopausal patient with invasive ductal carcinoma in the left breast (NST).
Use the current tumor formula: cT3 (suspicion of satellite lesions), cN0, M0 ER 100%, PR 100%, HER2-negative, Ki67 10%, G2
No therapy has been conducted so far.
No pre-existing conditions are known.
List the hypothetical treatment options in a table. Use rows for Surgical Therapy, Chemotherapy, Radiotherapy, Anti-HER2 Therapy, and Endocrine Therapy. Use columns for Recommendation (yes/no), hypothetical medication (substance and dosage), Alternatives, and Risks. Use bullet points in the table.
Keep it as concise as possible, avoid repeating the case or the question. Be precise. Summarize the tabular recommendation in a written sentence below the table.

Case 13 – Recommendation ChatGPT

**Therapy options**	**Recommendation**	**Medication (Active agent and dosage)**	**Alternatives**	**Risks**
Surgery	Yes	Mastectomy or breast-conserving surgery	Mastectomy	Infection, bleeding, scarring, seroma formation
Chemotherapy	Yes	Anthracycline- and taxane-based chemotherapy	-	Myeloid toxicity, nausea, hair loss, fatigue
Radiation	Yes	Radiation therapy of the chest wall	-	Skin reaction, fatigue
Anti-HER2-Therapy	No	No anti-HER2 therapy	-	-

Endocrine therapy	No	No endocrine therapy	-	-

The recommended therapy includes breast-conserving surgery or mastectomy as the surgical intervention, anthracycline- and taxane-based chemotherapy, and radiation therapy targeting the chest wall. Anti-HER2 therapy or endocrine therapy is not advised in this case. Risks and alternatives are detailed in the table.

The recommended therapy for the 32-year-old premenopausal patient with invasive ductal carcinoma in the left breast involves breast-conserving surgery or mastectomy, chemotherapy, and radiation therapy targeting the chest wall. Anti-HER2 therapy or endocrine therapy is not recommended. Risks and alternatives have been taken into account.

Fall 14:

Anamnese:
82-jährig
Z.n. Mammakarzinom rechts vor 24J, Brust-erhaltende Therapie + Axilladissektion rechts durchgeführt, Hormonrezeptor positiv, HER2-neu nicht bestimmt, postoperative Radiatio erfolgt, adjuvante endokrine Therapie für 3 Jahre erfolgt
Apendektomie (vor 70J)
Tonsillektomie (vor 60J)
Ovarektomie, einseitig (vor 60J)
Abrasio bei Hypermenorrhoe (vor 35J):
- Hysterektomie bei anschließender Massenblutung, lt. Pat. bds. Adnexektomie
Weitere Diagnosen:
PAVK rechts mit Stent (seit 10J)
Arterieller Hypertonus, Hyperlipidämie, Nikotinabsus (55 PY)
Familienanamnese: Leer für Brust- und Eierstockkrebs

Aktuelle Diagnose:
Tastbefund rechts, Kontrollhistologie Narbenkomplex
- Stanzbiopsie nach Referenzpathologischer Begutachtung mit V.a. Phylloidtumor

Prompt:
Bitte nenne auf Basis der folgenden URL: https://www.leitlinienprogramm-onkologie.de/fileadmin/user_upload/Downloads/Leitlinien/Mammakarzinom_4_0/Version_4.4/LL_Mammakarzinom_Langversion_4.4.pdf, die empfohlene Therapie einer hypothetischen 82-jährigen postmenopausalen Patientin mit einem Phylloidtumor der rechten Seite.
Keine aktuelle Tumorformel vorhanden.
Es wurde bisher keine Therapie durchgeführt.
Berücksichtige die Vorgeschichte der hypothetischen Patientin mit einem Mammakarzinom rechts vor 24 Jahren, dass brusterhaltend operiert, strahlentherapiert und adjuvant endokrin therapiert wurde.
Als Vorerkrankungen sind eine arterielle Hypertonie und Hyperlipidämie bekannt.
Nenne die hypothetischen Therapieoptionen in einer Tabelle. Nutze als Zeilen Operative Therapie, Chemotherapie, Strahlentherapie, Anti-Her2-Therapie und endokrine Therapie. Nutze als Spalten Empfehlung (ja/nein), das hypothetische Medikament (Wirkstoff und Dosierung), Alternativen und Risiken. Verwende in der Tabelle nur Stichpunkte.
Halte dich so kurz wie möglich, wiederhole nicht den Fall oder die Frage. Sei präzise. Fasse die tabellarische Empfehlung in einem ausgeschriebenen Satz unter der Tabelle zusammen.

Fall 14 – Empfehlung ChatGPT:

Therapieoptionen	Empfehlung	Medikament (Wirkstoff und Dosierung)	Alternativen	Risiken
Operative Therapie	Ja	Tumorresektion	-	Blutungen, Infektionen, Nachblutung
Chemotherapie	Nein	Keine Chemotherapie	-	-
Strahlentherapie	Nein	Keine Strahlentherapie	-	-
Anti-HER2-Therapie	Nein	Keine Anti-HER2-Therapie	-	-
Endokrine Therapie	Nein	Keine endokrine Therapie	-	-

Die empfohlene Therapie für die 82-jährige postmenopausale Patientin mit einem Phylloidtumor der rechten Seite umfasst die operative Tumorresektion. Es wird keine Chemotherapie, Strahlentherapie, Anti-HER2-Therapie oder endokrine Therapie empfohlen. Risiken und Alternativen wurden berücksichtigt.

Die empfohlene Therapieoption für die 82-jährige postmenopausale Patientin mit einem Phylloidtumor der rechten Seite ist die operative Tumorresektion. Es werden keine weiteren Therapien wie Chemotherapie, Strahlentherapie, Anti-HER2-Therapie oder endokrine Therapie empfohlen. Risiken und Alternativen wurden berücksichtigt.

Translated version:

Case 14

Medical History:
82 years old
History of right breast cancer 24 years ago, underwent breast-conserving therapy + right axillary dissection, hormone receptor-positive, HER2-neu not determined, postoperative radiation performed, adjuvant endocrine therapy for 3 years
Appendectomy (70 years ago)
Tonsillectomy (60 years ago)
Oophorectomy, unilateral (60 years ago)
Dilation and curettage for hypermenorrhea (35 years ago):
- Hysterectomy due to subsequent massive bleeding, according to the patient, bilateral adnexectomy performed

Other diagnoses:
Peripheral arterial disease (PAD) on the right with stent (for 10 years)
Arterial hypertension, hyperlipidemia, nicotine abuse (55 pack-years)
Family history: Negative for breast and ovarian cancer

Current Diagnosis:
Palpation finding on the right, control histology of scar complex
- Punch biopsy after reference pathological assessment with suspected phyllodes tumor

Prompt:
Please provide, based on the following URL: https://www.leitlinienprogramm-onkologie.de/fileadmin/user_upload/Downloads/Leitlinien/Mammakarzinom_4_0/Version_4.4/LL_Mammakarzinom_Langversion_4.4.pdf, the recommended therapy for a hypothetical 82-year-old postmenopausal patient with a phyllodes tumor on the right side.
No current tumor formula available.
No therapy has been performed so far.
Take into account the hypothetical patient's history with right breast cancer 24 years ago, treated with breast-conserving surgery, radiotherapy, and adjuvant endocrine therapy.
Known pre-existing conditions include arterial hypertension and hyperlipidemia.
List the hypothetical treatment options in a table. Use rows for Surgical Therapy, Chemotherapy, Radiotherapy, Anti-HER2 Therapy, and Endocrine Therapy. Use columns for Recommendation (yes/no), hypothetical medication (substance and dosage), Alternatives, and Risks. Use bullet points in the table.
Keep it as concise as possible, avoid repeating the case or the question. Be precise. Summarize the tabular recommendation in a written sentence below the table.

Case 14 – Recommendation ChatGPT

**Therapy options**	**Recommendation**	**Medication (Active agent and dosage)**	**Alternatives**	**Risks**
Surgery	Yes	Tumor resection	-	Bleeding, infection
Chemotherapy	No	No chemotherapy	-	-
Radiation	No	No radiation	-	-
Anti-HER2-Therapy	No	No anti-HER2 therapy	-	-
Endocrine therapy	No	No endocrine therapy	-	-

The recommended therapy for the 82-year-old postmenopausal patient with a phyllodes tumor on the right side includes surgical tumor resection. Chemotherapy, radiotherapy, anti-HER2 therapy, or endocrine therapy is not recommended. Risks and alternatives have been considered.

The recommended treatment option for the 82-year-old postmenopausal patient with a phyllodes tumor on the right side is surgical tumor resection. No additional therapies such as chemotherapy, radiotherapy, anti-HER2 therapy, or endocrine therapy are recommended. Risks and alternatives have been considered.

Fall 15:

Anamnese:
65-jährig, G0/P0
Keine Vorerkrankungen
Familienanamnese: leer
Medikamente: keine

Aktuelle Diagnose:
Invasives Mammakarzinom links NST
cT3 (71x52mm), cN1, cMx
ER 15%, PR 0%, HER2-negativ, Ki67 ca. 65%

Prompt:
Bitte nenne auf Basis der folgenden URL: https://www.leitlinienprogramm-onkologie.de/fileadmin/user_upload/Downloads/Leitlinien/Mammakarzinom_4_0/Version_4.4/LL_Mammakarzinom_Langversion_4.4.pdf, die empfohlene Therapie einer hypothetischen 65-jährigen postmenopausalen Patientin mit einem invasiven Mammakarzinom links (NST).
Nutze die aktuelle Tumorformel: cT3 (71x52mm), cN1, cMx, ER 15%, PR 0%, HER2-negativ, Ki67 ca. 65%
Es wurde bisher keine Therapie durchgeführt.
Es sind keine Vorerkrankungen bekannt.
Nenne die hypothetischen Therapieoptionen in einer Tabelle. Nutze als Zeilen Operative Therapie, Chemotherapie, Strahlentherapie, Anti-Her2-Therapie und endokrine Therapie. Nutze als Spalten Empfehlung (ja/nein), das hypothetische Medikament (Wirkstoff und Dosierung), Alternativen und Risiken. Verwende in der Tabelle nur Stichpunkte.
Halte dich so kurz wie möglich, wiederhole nicht den Fall oder die Frage. Sei präzise. Fasse die tabellarische Empfehlung in einem ausgeschriebenen Satz unter der Tabelle zusammen.

Fall 15 – Empfehlung ChatGPT:

Therapieoptionen	Empfehlung	Medikament (Wirkstoff und Dosierung)	Alternativen	Risiken
Operative Therapie	Ja	Brustamputation oder brusterhaltende Therapie	-	Blutungen, Infektionen, Wundheilungsstörung
Chemotherapie	Ja	Anthrazyklin-basierte oder Taxan-basierte Therapie	-	Knochenmarkssuppressic Übelkeit, Haarausfall
Strahlentherapie	Ja	Externe Radiotherapie	-	Hautreaktionen, Müdigkei
Anti-HER2-Therapie	Nein	Keine Anti-HER2-Therapie	-	-
Endokrine Therapie	Ja	Aromatasehemmer oder Tamoxifen	-	Östrogenmangel, Wechselwirkungen mit anderen Medikamenten

Die empfohlene Therapie für die 65-jährige postmenopausale Patientin mit einem invasiven Mammakarzinom links (NST) umfasst eine operative Therapie, entweder in Form einer Brustamputation oder brusterhaltenden Therapie. Es wird auch eine Chemotherapie empfohlen, entweder mit Anthrazyklin-basierten oder Taxan-basierten Medikamenten. Eine Strahlentherapie sollte ebenfalls durchgeführt werden. Eine Anti-HER2-Therapie ist nicht notwendig, da der HER2-Status negativ ist. Zusätzlich wird eine endokrine Therapie mit Aromatasehemmern oder Tamoxifen empfohlen. Risiken und Alternativen wurden berücksichtigt.

Die empfohlene Therapieoption für die 65-jährige postmenopausale Patientin mit einem invasiven Mammakarzinom links (NST) umfasst eine operative Therapie (Brustamputation oder brusterhaltende Therapie), Chemotherapie (Anthrazyklin-basiert oder Taxan-basiert), Strahlentherapie sowie endokrine Therapie (Aromatasehemmer oder Tamoxifen). Eine Anti-HER2-Therapie ist nicht erforderlich. Risiken und Alternativen wurden berücksichtigt.

Translated version:

Case 15

Medical History:
65 years old, G0/P0
No pre-existing conditions
Family history: empty
Medications: none

Current diagnosis:
Invasive ductal carcinoma, left breast NST
cT3 (71x52mm), cN1, cMx
ER 15%, PR 0%, HER2-negative, Ki67 approx. 65%

Prompt:
Please provide, based on the following URL: https://www.leitlinienprogramm-onkologie.de/fileadmin/user_upload/Downloads/Leitlinien/Mammakarzinom_4_0/Version_4.4/LL_Mammakarzinom_Langversion_4.4.pdf, the recommended therapy for a hypothetical 65-year-old postmenopausal patient with invasive ductal carcinoma in the left breast (NST).
Use the current tumor formula: cT3 (71x52mm), cN1, cMx, ER 15%, PR 0%, HER2-negative, Ki67 approx. 65%
No therapy has been conducted so far.
No pre-existing conditions are known.
List the hypothetical treatment options in a table. Use rows for Surgical Therapy, Chemotherapy, Radiotherapy, Anti-HER2 Therapy, and Endocrine Therapy. Use columns for Recommendation (yes/no), hypothetical medication (substance and dosage), Alternatives, and Risks. Use bullet points in the table.
Keep it as concise as possible, avoid repeating the case or the question. Be precise. Summarize the tabular recommendation in a written sentence below the table.

Case 15 – Recommendation ChatGPT

**Therapy options**	**Recommendation**	**Medication (Active agent and dosage)**	**Alternatives**	**Risks**
Surgery	Yes	Mastectomy or breast-conserving surgery	-	Impaired wound healing, bleeding, infection
Chemotherapy	Yes	Anthracycline- or taxane-based chemotherapy	-	Myeloid toxicity, nausea, hair loss
Radiation	Yes	External radiation	-	Skin reaction, fatigue
Anti-HER2-Therapy	No	No anti-HER2 therapy	-	-
Endocrine therapy	Yes	Aromatase inhibitor or tamoxifen	-	Estrogen deficiency, interference with other medications

The recommended therapy for the 65-year-old postmenopausal patient with invasive ductal carcinoma in the left breast (NST) includes surgical intervention, either in the form of mastectomy or breast-conserving therapy. Chemotherapy is also recommended, either with anthracycline-based or taxane-based medications. Radiation

therapy should also be performed. Anti-HER2 therapy is not necessary as the HER2 status is negative. Additionally, endocrine therapy with aromatase inhibitors or tamoxifen is recommended. Risks and alternatives have been taken into account.

The recommended therapy option for the 65-year-old postmenopausal patient with invasive ductal carcinoma in the left breast (NST) includes surgical intervention (mastectomy or breast-conserving therapy), chemotherapy (anthracycline-based or taxane-based), radiation therapy, and endocrine therapy (aromatase inhibitors or tamoxifen). Anti-HER2 therapy is not required. Risks and alternatives have been considered.

Fall 16:

Anamnese:
58-Jährige, G4/P2, postmenopausal (seit 6J)
-LINKS: Invasives Mammakarzinom (NST) ED vor 4 Jahren, subkutane Mastektomie mit autologer Konversion nach mammographischer Drahtmarkierung und Sentinellymphonodektomie Axilla links
-RECHTS: Angleichende Mammareduktionsplastik
2 Jahre Endokrinen Therapie, Abbruch vor 2 Jahren gegenüber Letrozol

Bekanntes Lynch-Syndrom (HNPCC-Syndrom)
Urothel-Karzinom (vor 9J)
- (Harnblasen-Harnleiter-Karzinom), partielle Ureterektomie, TUR Blase
- T0, lokale Chemo
Bekannte Thrombophilie mit Faktor-V: Q506-Heterozygotie
Beta-Fibrinogen-Hae-III-Heterozygotie
Protein-C-2405C-Homozygotie
Lungenembolie (vor12J), Thrombose untere Extremität und Becken, Rezidivierende Phlebitiden rechtes Bein
Lipoprotein(a)-Erhöhung, Adipositas
Hypothyreose
rezidivierende depressive Störung, Angststörung
Allergien: Heuschnupfen, Asthma, Nahrungsmittel, braunes Pflaster.
Medikamente: L-Thyroxin, Marcumar

Familiäre Karzinomanamnese:
Mutter mit 48J Uterus-Kolon-Mamma-Ca, Tante Uterus-Kolon-Ca, Großvater Kolon-Ca, Vater Prostata- CA (75J), Cousine der Mutter mit 50 Jahren Mamma-CA, Vater d. Mutter mit 36 und 63 Jahren Colon-CA

Aktuelle Diagnose:
Trifokales Mamma-Ca Rezidiv links, NST bei HNPCC-Syndrom (heterozygote MSH2-Mutation) und VUS im BRCA2-Gen; rcTc (m=3, bis 12 mm), cN0, M0
I) NST links präpektoral, Clip markiert
- G2, ER 12%, PR 0%, HER2-negativ, Ki67 15%
II) NST links präpektoral:, Clip markiert
- G2, ER 12%, PR 0%, HER2-negativ, Ki67 18%
III) Mamma links präpektoral: keine Stanze erfolgt

Aktuell: pT2 (2,3 cm), pN0 (0/4 sn), G2, L0, V0, Pn0, R0
ER 15%; PR 0%; HER2-negativ, Ki67 ca. 20%.
Intrinsischer Subtyp (St Gallen 2015): luminal A/B

Prompt:
Bitte nenne auf Basis der folgenden URL: https://www.leitlinienprogramm-onkologie.de/fileadmin/user_upload/Downloads/Leitlinien/Mammakarzinom_4_0/Version_4.4/LL_Mammakarzinom_Langversion_4.4.pdf, die empfohlene Therapie einer hypothetischen 58-jährigen postmenopausalen Patientin mit einem trifokalem Mammakarzinomrezidiv links.
Nutze die aktuelle Tumorformel: pT2 (2,3 cm), pN0 (0/4 sn), G2, L0, V0, Pn0, R0, ER 15%; PR 0%; HER2-negativ, Ki67 ca. 20%
Es wurde bisher keine Therapie durchgeführt.
Berücksichtige die Vorgeschichte der hypothetischen Patientin mit einem invasiven Mammakarzinom der linken Brust, dass vor 4 Jahren brusterhaltend therapiert wurde.

Als Vorerkrankungen sind ein Lynch-Syndrom, eine Thrombophilie, eine Lungenembolie, eine Hypothyreose und eine depressive Störung bekannt.
Nenne die hypothetischen Therapieoptionen in einer Tabelle. Nutze als Zeilen Operative Therapie, Chemotherapie, Strahlentherapie, Anti-Her2-Therapie und endokrine Therapie. Nutze als Spalten Empfehlung (ja/nein), das hypothetische Medikament (Wirkstoff und Dosierung), Alternativen und Risiken. Verwende in der Tabelle nur Stichpunkte.
Halte dich so kurz wie möglich, wiederhole nicht den Fall oder die Frage. Sei präzise. Fasse die tabellarische Empfehlung in einem ausgeschriebenen Satz unter der Tabelle zusammen.

Fall 16 – Empfehlung ChatGPT:

Therapieoptionen	Empfehlung	Medikament (Wirkstoff und Dosierung)	Alternativen	Risiken
Operative Therapie	Ja	Mastektomie oder brusterhaltende Therapie	-	Blutungen, Infektionen, Wundheilungsstörung
Chemotherapie	Ja	Anthrazyklin-basierte oder Taxan-basierte Therapie	-	Knochenmarkssuppressi Übelkeit, Haarausfall
Strahlentherapie	Ja	Externe Radiotherapie	-	Hautreaktionen, Müdigkei
Anti-HER2-Therapie	Nein	Keine Anti-HER2-Therapie	-	-
Endokrine Therapie	Ja	Aromatasehemmer oder Tamoxifen	-	Östrogenmangel, Wechselwirkungen mit anderen Medikamenten

Die empfohlene Therapie für die 58-jährige postmenopausale Patientin mit einem trifokalen Mammakarzinomrezidiv links umfasst eine operative Therapie, entweder in Form einer Mastektomie oder brusterhaltenden Therapie. Es wird auch eine Chemotherapie empfohlen, entweder mit Anthrazyklin-basierten oder Taxan-basierten Medikamenten. Eine Strahlentherapie sollte ebenfalls durchgeführt werden. Eine Anti-HER2-Therapie ist nicht erforderlich, da der HER2-Status negativ ist. Zusätzlich wird eine endokrine Therapie mit Aromatasehemmern oder Tamoxifen empfohlen. Risiken und Alternativen wurden berücksichtigt.

Die empfohlene Therapieoption für die 58-jährige postmenopausale Patientin mit einem trifokalen Mammakarzinomrezidiv links umfasst eine operative Therapie (Mastektomie oder brusterhaltende Therapie), Chemotherapie (Anthrazyklin-basiert oder Taxan-basiert), Strahlentherapie sowie endokrine Therapie (Aromatasehemmer oder Tamoxifen). Eine Anti-HER2-Therapie ist nicht erforderlich. Risiken und Alternativen wurden berücksichtigt.

Translated version:

Case 16

Medical History:
58-year-old, G4/P2, postmenopausal (for 6 years)
- LEFT: Invasive ductal carcinoma (NST) diagnosed 4 years ago, underwent subcutaneous mastectomy with autologous conversion after mammographic wire marking, and sentinel lymph node dissection in the left axilla.
- RIGHT: Concurrent breast reduction surgery.
Endocrine therapy for 2 years, discontinued 2 years ago (Letrozole).

Known Lynch syndrome (HNPCC syndrome)
Urothelial carcinoma (9 years ago)
- (Bladder-ureter carcinoma), partial ureterectomy, TUR bladder
- T0, local chemotherapy
Known thrombophilia with Factor V: Q506 heterozygosity
Beta-fibrinogen Hae-III heterozygosity
Protein C-2405C homozygosity
Pulmonary embolism (12 years ago), thrombosis in lower extremity and pelvis, recurrent phlebitis in the right leg
Elevated lipoprotein(a), obesity
Hypothyroidism
Recurrent depressive disorder, anxiety disorder
Allergies: Hay fever, asthma, food, brown patch.
Medications: L-Thyroxine, Marcumar (warfarin)

Family Cancer History:
Mother with uterine-colon-breast cancer at 48, aunt with uterine-colon cancer, grandfather with colon cancer, father with prostate cancer (at 75), mother's cousin with breast cancer at 50, mother's father with colon cancer at 36 and 63.

Current Diagnosis:
Trifocal breast cancer recurrence on the left, NST in the context of HNPCC syndrome (heterozygous MSH2 mutation), and VUS in the BRCA2 gene; rcTc (m=3, up to 12 mm), cN0, M0
I) NST on the left prepectoral, clip marked
- G2, ER 12%, PR 0%, HER2-negative, Ki67 15%
II) NST on the left prepectoral, clip marked
- G2, ER 12%, PR 0%, HER2-negative, Ki67 18%
III) Breast on the left prepectoral: no biopsy performed

Currently: pT2 (2.3 cm), pN0 (0/4 sn), G2, L0, V0, Pn0, R0
ER 15%; PR 0%; HER2-negative, Ki67 approximately 20%.
Intrinsic subtype (St. Gallen 2015): luminal A/B

Prompt:
Based on the following URL: https://www.leitlinienprogramm-onkologie.de/fileadmin/user_upload/Downloads/Leitlinien/Mammakarzinom_4_0/Version_4.4/LL_Mammakarzinom_Langversion_4.4.pdf, please provide the recommended treatment for a hypothetical 58-year-old postmenopausal patient with a trifocal recurrence of breast cancer on the left.
Utilize the current tumor formula: pT2 (2.3 cm), pN0 (0/4 sn), G2, L0, V0, Pn0, R0, ER 15%; PR 0%; HER2-negative, Ki67 approximately 20%.

No treatment has been administered so far. Consider the patient's history of invasive breast cancer on the left, which was treated conservatively 4 years ago.
The patient's medical history includes Lynch syndrome, thrombophilia, pulmonary embolism, hypothyroidism, and depressive disorder.
List the hypothetical treatment options in a table. Use rows for Surgical Therapy, Chemotherapy, Radiotherapy, Anti-HER2 Therapy, and Endocrine Therapy. Use columns for Recommendation (yes/no), hypothetical medication (substance and dosage), Alternatives, and Risks. Use bullet points in the table.
Keep it as concise as possible, avoid repeating the case or the question. Be precise. Summarize the tabular recommendation in a written sentence below the table.

Case 16 – Recommendation ChatGPT

**Therapy options**	**Recommendation**	**Medication (Active agent and dosage)**	**Alternatives**	**Risks**
Surgery	Yes	Mastectomy or breast-conserving surgery	-	Impaired wound healing, bleeding, infection
Chemotherapy	Yes	Anthracycline- or taxane-based chemotherapy	-	Myeloid toxicity, nausea, hair loss
Radiation	Yes	External radiation	-	Skin reaction, fatigue
Anti-HER2-Therapy	No	No anti-HER2 therapy	-	-
Endocrine therapy	Yes	Aromatase inhibitor or tamoxifen	-	Estrogen deficiency, interference with other medications

The recommended treatment for the 58-year-old postmenopausal patient with a trifocal recurrence of breast cancer on the left includes surgical intervention, either in the form of a mastectomy or breast-conserving therapy. Chemotherapy is also recommended, either with anthracycline-based or taxane-based medications. Radiation therapy should also be performed. Anti-HER2 therapy is not required as the HER2 status is negative. Additionally, endocrine therapy with aromatase inhibitors or tamoxifen is recommended. Risks and alternatives have been considered.

The recommended treatment option for the 58-year-old postmenopausal patient with a trifocal recurrence of breast cancer on the left includes surgical intervention (mastectomy or breast-conserving therapy), chemotherapy (anthracycline-based or taxane-based), radiation therapy, and endocrine therapy (aromatase inhibitors or tamoxifen). Anti-HER2 therapy is not required. Risks and alternatives have been considered.

Fall 17:

Anamnese:
66-jährige, postmenopausale G2/P2 (2x Spontanpartus)
Z.n. Covid-Infektion (Pat. ist 2x geimpft)
Varicosis Beine bds.
Familienanamnese: leer für Karzinome
Allergien: keine
Rauchen: nein

Aktuelle Diagnose:
Invasives Mammakarzinom (NST) mit lobulär-ähnlichem Wachstumsmuster rechts
Aktuell: pT1b (1,0 cm), pN0 (0/1 sn), G2, L0, V0, Pn0, R0
ER 100%, PR <1%, HER2-negativ, Ki67 ca. 20%

Initial: cT1c (1,14 cm), cN0, MX, G2
ER 100%, PR 0%, HER2-negativ, Ki67 ca. 20%

Operation:
Brust-erhaltene Therapie rechts mit Sentinellymphonodektomie rechts

Prompt:
Bitte nenne auf Basis der folgenden URL: https://www.leitlinienprogramm-onkologie.de/fileadmin/user_upload/Downloads/Leitlinien/Mammakarzinom_4_0/Version_4.4/LL_Mammakarzinom_Langversion_4.4.pdf, die empfohlene Therapie einer hypothetischen 66-jährigen postmenopausalen Patientin mit einem invasiven Mammakarzinom rechts (NST).
Nutze die aktuelle Tumorformel: pT1b (1,0 cm), pN0 (0/1 sn), G2, L0, V0, Pn0, R0, ER 100%, PR <1%, HER2-negativ, Ki67 ca. 20%
Es wurde bereits eine brusterhaltene Therapie rechts mit Sentinellymphonodektomie rechts durchgeführt.
Es sind keine Vorerkrankungen bekannt.
Nenne die hypothetischen Therapieoptionen in einer Tabelle. Nutze als Zeilen Operative Therapie, Chemotherapie, Strahlentherapie, Anti-Her2-Therapie und endokrine Therapie. Nutze als Spalten Empfehlung (ja/nein), das hypothetische Medikament (Wirkstoff und Dosierung), Alternativen und Risiken. Verwende in der Tabelle nur Stichpunkte.
Halte dich so kurz wie möglich, wiederhole nicht den Fall oder die Frage. Sei präzise. Fasse die tabellarische Empfehlung in einem ausgeschriebenen Satz unter der Tabelle zusammen.

Fall 17 – Empfehlung ChatGPT:

Therapieoptionen	Empfehlung	Medikament (Wirkstoff und Dosierung)	Alternativen	Risiken
Operative Therapie	Ja	Brusterhaltende Therapie oder Mastektomie	-	Blutungen, Infektionen, Wundheilungsstörung
Chemotherapie	Nein	Keine Chemotherapie	-	-
Strahlentherapie	Ja	Externe Radiotherapie	-	Hautreaktionen, Müdigkeit
Anti-HER2-Therapie	Nein	Keine Anti-HER2-Therapie	-	-
Endokrine Therapie	Ja	Aromatasehemmer oder Tamoxifen	-	Östrogenmangel, Wechselwirkungen mit anderen Medikamenten

Die empfohlene Therapie für die 66-jährige postmenopausale Patientin mit einem invasiven Mammakarzinom rechts (NST) umfasst eine brusterhaltende Therapie oder Mastektomie als operative Therapie. Eine Chemotherapie ist in diesem Fall nicht erforderlich. Es wird eine Strahlentherapie empfohlen. Eine Anti-HER2-Therapie ist nicht notwendig, da der HER2-Status negativ ist. Zusätzlich wird eine endokrine Therapie mit Aromatasehemmern oder Tamoxifen empfohlen. Risiken und Alternativen wurden berücksichtigt.

Die empfohlene Therapieoption für die 66-jährige postmenopausale Patientin mit einem invasiven Mammakarzinom rechts (NST) umfasst eine brusterhaltende Therapie oder Mastektomie als operative Therapie, eine Strahlentherapie sowie endokrine Therapie mit Aromatasehemmern oder Tamoxifen. Eine Chemotherapie oder Anti-HER2-Therapie ist in diesem Fall nicht erforderlich. Risiken und Alternativen wurden berücksichtigt.

Translated version:

Case 17

Medical History:
66 years old, postmenopausal G2/P2 (2 spontaneous deliveries)
History of Covid infection (patient is vaccinated twice)
Bilateral leg varicose veins
Family history: No history of carcinomas
Allergies: None
Smoking: No

Current Diagnosis:
Invasive ductal carcinoma (NST) with lobular-like growth pattern on the right
Current: pT1b (1.0 cm), pN0 (0/1 sn), G2, L0, V0, Pn0, R0
ER 100%, PR <1%, HER2-negative, Ki67 approximately 20%

Initial: cT1c (1.14 cm), cN0, MX, G2
ER 100%, PR 0%, HER2-negative, Ki67 approximately 20%

Surgery:
Breast-conserving therapy on the right with right sentinel lymph node dissection

Prompt:
Please provide, based on the following URL: https://www.leitlinienprogramm-onkologie.de/fileadmin/user_upload/Downloads/Leitlinien/Mammakarzinom_4_0/Version_4.4/LL_Mammakarzinom_Langversion_4.4.pdf, the recommended treatment for a hypothetical 66-year-old postmenopausal patient with an invasive ductal carcinoma on the right (NST).
Use the current tumor formula: pT1b (1.0 cm), pN0 (0/1 sn), G2, L0, V0, Pn0, R0, ER 100%, PR <1%, HER2-negative, Ki67 approximately 20%
A breast-conserving therapy on the right with sentinel lymph node dissection has already been performed.
No other pre-existing conditions are known.
List the hypothetical treatment options in a table. Use rows for Surgical Therapy, Chemotherapy, Radiotherapy, Anti-HER2 Therapy, and Endocrine Therapy. Use columns for Recommendation (yes/no), hypothetical medication (substance and dosage), Alternatives, and Risks. Use bullet points in the table.
Keep it as concise as possible, avoid repeating the case or the question. Be precise. Summarize the tabular recommendation in a written sentence below the table.

Case 17 – Recommendation ChatGPT

**Therapy options**	**Recommendation**	**Medication (Active agent and dosage)**	**Alternatives**	**Risks**
Surgery	Yes	Mastectomy or breast-conserving surgery	-	Impaired wound healing, bleeding, infection
Chemotherapy	No	No chemotherapy	-	-
Radiation	Yes	External radiation	-	Skin reaction, fatigue
Anti-HER2-Therapy	No	No anti-HER2 therapy	-	-

Endocrine therapy	Yes	Aromatase inhibitor or tamoxifen	-	Estrogen deficiency, interference with other medications

The recommended treatment for the 66-year-old postmenopausal patient with invasive ductal carcinoma on the right (NST) includes breast-conserving therapy or mastectomy as the surgical intervention. Chemotherapy is not necessary in this case. Radiation therapy is recommended. Anti-HER2 therapy is not required since the HER2 status is negative. Additionally, endocrine therapy with aromatase inhibitors or tamoxifen is recommended. Risks and alternatives have been considered.

The recommended treatment option for the 66-year-old postmenopausal patient with invasive ductal carcinoma on the right (NST) includes breast-conserving therapy or mastectomy as the surgical intervention, radiation therapy, and endocrine therapy with aromatase inhibitors or tamoxifen. Chemotherapy or anti-HER2 therapy is not necessary in this case. Risks and alternatives have been considered.

Fall 18:

Anamnese:
67-jährige Nullipara, postmenopausal
Arterieller Hypertonus
latente Hypothyreose
Z.n. Darm-OP bei Blutung vor 5J
Allergien: Nickel, Chrom
Medikamente: Candesartan, ASS 100,
Nikotinabusus: 5-7 Zigaretten / Tag
Familienanamnese: Onkel (v) Bronchialkarzinom

Aktuelle Diagnose:
ED disseminierte Lebermetastasierung, Hautmetastasierung
Invasives Mammakarzinom rechts NST ED vor 3 Jahren
Aktuell: pT1b (1,0 cm Durchmesser), pTis (3,5 cm Durchmesser), G3, pN0 (0/2 sn), M1, L0, V0, Pn0, R0 (invasives Karzinom und DCIS).
Immunphänotyp: ER 100%; PR 0%; HER2-negativ; Ki67 ca. 30%.
Intrinsischer Subtyp: Luminal B/A

Epikrise:
Invasives Mammakarzinom rechts NST ED vor 3 Jahren sowie Z.n. BET rechts + SLN rechts (vor 3J)
Postoperative-adjuvante hypofraktionierte Bestrahlung der rechten Mamma ad 40,05 Gy (ED 2,67 Gy), anschließend sequentieller Boost der ehemaligen Tumorregion ad 50,05 Gy kumulativ. (ED 2,0 Gy)
Keine adjuvante Chemotherapie
Derzeit antihormonelle Therapie mit Anastrozol

Prompt:
Bitte nenne auf Basis der folgenden URL: https://www.leitlinienprogramm-onkologie.de/fileadmin/user_upload/Downloads/Leitlinien/Mammakarzinom_4_0/Version_4.4/LL_Mammakarzinom_Langversion_4.4.pdf, die empfohlene Therapie einer hypothetischen 67-jährigen postmenopausalen Patientin mit einer disseminierten Lebermetastasierung und Hautmetastasierung eines invasiven Mammakarzinoms rechts.
Nutze die aktuelle Tumorformel: pT1b (1,0 cm Durchmesser), pTis (3,5 cm Durchmesser), G3, pN0 (0/2 sn), M1, L0, V0, Pn0, R0. ER 100%; PR 0%; HER2-negativ; Ki67 ca. 30%.
Es wurde bereits eine brusterhaltende Therapie und Sentinellymphonodektomie der rechten Seite vor 3 Jahren durchgeführt.
Berücksichtige die Vorgeschichte der hypothetischen Patientin mit bereits durchgeführter adjuvanter Bestrahlung und antihormoneller Therapie mit Anastrozol. Eine Chemotherapie wurde bisher nicht durchgeführt.
Als Vorerkrankungen sind eine arterielle Hypertonie und eine Hypothyreose bekannt.
Nenne die hypothetischen Therapieoptionen in einer Tabelle. Nutze als Zeilen Operative Therapie, Chemotherapie, Strahlentherapie, Anti-Her2-Therapie und endokrine Therapie. Nutze als Spalten Empfehlung (ja/nein), das hypothetische Medikament (Wirkstoff und Dosierung), Alternativen und Risiken. Verwende in der Tabelle nur Stichpunkte.
Halte dich so kurz wie möglich, wiederhole nicht den Fall oder die Frage. Sei präzise. Fasse die tabellarische Empfehlung in einem ausgeschriebenen Satz unter der Tabelle zusammen.

Fall 18 – Empfehlung ChatGPT:

Therapieoptionen	Empfehlung	Medikament (Wirkstoff und Dosierung)	Alternativen	Risiken
Operative Therapie	Nein	Keine operative Therapie	-	-
Chemotherapie	Ja	Systemische Chemotherapie (z. B. Paclitaxel)	Alternativen	Knochenmarksuppression, Übelkeit, Haarausfall
Strahlentherapie	Ja	Palliative Strahlentherapie	-	Müdigkeit, Hautreaktionen
Anti-HER2-Therapie	Nein	Keine Anti-HER2-Therapie	-	-
Endokrine Therapie	Nein	Keine endokrine Therapie	-	-

Die empfohlene Therapieoption für die 67-jährige postmenopausale Patientin mit einer disseminierten Lebermetastasierung und Hautmetastasierung eines invasiven Mammakarzinoms rechts umfasst eine systemische Chemotherapie, wie zum Beispiel Paclitaxel, zur Behandlung der Metastasen. Zusätzlich wird eine palliative Strahlentherapie zur Linderung von Symptomen empfohlen. Es ist keine Anti-HER2-Therapie oder endokrine Therapie erforderlich. Risiken und Alternativen wurden berücksichtigt.

Die empfohlene Therapieoption für die 67-jährige postmenopausale Patientin mit einer disseminierten Lebermetastasierung und Hautmetastasierung eines invasiven Mammakarzinoms rechts umfasst eine systemische Chemotherapie mit Paclitaxel sowie eine palliative Strahlentherapie. Es ist keine operative Therapie, Anti-HER2-Therapie oder endokrine Therapie erforderlich. Risiken und Alternativen wurden berücksichtigt.

Translated version:

Case 18

Medical history:
67-year-old nulliparous, postmenopausal
Arterial hypertension
Latent hypothyroidism
History of colorectal surgery due to bleeding 5 years ago
Allergies: Nickel, Chrome
Medications: Candesartan, Aspirin 100 mg
Nicotine use: 5-7 cigarettes/day
Family history: Uncle (maternal) with bronchial carcinoma

Current Diagnosis:
ED disseminated liver metastasis, skin metastasis
Invasive ductal carcinoma on the right, NST, ED, 3 years ago
Current: pT1b (1.0 cm diameter), pTis (3.5 cm diameter), G3, pN0 (0/2 sn), M1, L0, V0, Pn0, R0 (invasive carcinoma and DCIS).
Immunophenotype: ER 100%, PR 0%, HER2-negative, Ki67 approximately 30%.
Intrinsic subtype: Luminal B/A

Epikrisis:
Invasive ductal carcinoma on the right, NST, ED, 3 years ago, and history of breast-conserving therapy on the right + sentinel lymph node biopsy (3 years ago).
Postoperative-adjuvant hypofractionated radiation therapy to the right breast at 40.05 Gy (ED 2.67 Gy), followed by a sequential boost to the former tumor region at 50.05 Gy cumulative (ED 2.0 Gy).
No adjuvant chemotherapy
Currently undergoing antihormonal therapy with Anastrozole

Prompt:
Please provide, based on the following URL: https://www.leitlinienprogramm-onkologie.de/fileadmin/user_upload/Downloads/Leitlinien/Mammakarzinom_4_0/Version_4.4/LL_Mammakarzinom_Langversion_4.4.pdf, the recommended treatment for a hypothetical 67-year-old postmenopausal patient with disseminated liver and skin metastases of an invasive ductal carcinoma on the right.
Use the current tumor formula: pT1b (1.0 cm diameter), pTis (3.5 cm diameter), G3, pN0 (0/2 sn), M1, L0, V0, Pn0, R0. ER 100%, PR 0%, HER2-negative, Ki67 approximately 30%.
A breast-conserving therapy and sentinel lymph node biopsy on the right were performed 3 years ago. Consider the patient's history of prior adjuvant radiation therapy and antihormonal treatment with Anastrozole. Chemotherapy has not been administered to date.
Known pre-existing conditions include arterial hypertension and hypothyroidism.
List the hypothetical treatment options in a table. Use rows for Surgical Therapy, Chemotherapy, Radiotherapy, Anti-HER2 Therapy, and Endocrine Therapy. Use columns for Recommendation (yes/no), hypothetical medication (substance and dosage), Alternatives, and Risks. Use bullet points in the table.
Keep it as concise as possible, avoid repeating the case or the question. Be precise. Summarize the tabular recommendation in a written sentence below the table.

Case 18 – Recommendation ChatGPT

**Therapy options**	**Recommendation**	**Medication (Active agent and dosage)**	**Alternatives**	**Risks**
Surgery	No	No surgery	-	-
Chemotherapy	Yes	Systemic chemotherapy (e.g. paclitaxel)	Alternatives	Myeloid toxicity, nausea, hair loss
Radiation	Yes	Palliative radiation therapy	-	Skin reaction, fatigue
Anti-HER2-Therapy	No	No anti-HER2 therapy	-	-
Endocrine therapy	No	No endocrine therapy	-	-

The recommended treatment option for the 67-year-old postmenopausal patient with disseminated liver and skin metastases of an invasive ductal carcinoma on the right involves systemic chemotherapy, such as Paclitaxel, for the treatment of the metastases. Additionally, palliative radiation therapy is recommended to alleviate symptoms. No anti-HER2 therapy or endocrine therapy is required. Risks and alternatives have been considered.

The recommended treatment option for the 67-year-old postmenopausal patient with disseminated liver and skin metastases of an invasive ductal carcinoma on the right includes systemic chemotherapy with Paclitaxel as well as palliative radiation therapy. No surgical intervention, anti-HER2 therapy, or endocrine therapy is required. Risks and alternatives have been considered.

Fall 19:

Anamnese:
42-jährige, prämenopausale G3/P3
Z.n. Thrombophlebitis und Lymphangitis linker Arm
Z.n. Mammaaugmentation mit Allergan 365g retropektoraler Implantateinlage bds vor 10J
Familienanamnese: leer
Z.n. Nikotinabusus (1 Packung/ Tag)
Z.n. Liposuktion
Internistisch gesund

Aktuelle Diagnose:
2. Rezidiv des vorbekannten Mammakarzinoms i.S. von neu aufgetretenen Hautmetastasen im Bereich beider Narben
Initial multizentrisches Mammakarzinom links NST vor 3J
ypT1a (max. 0,2 cm), ypTis (max. 10,5 cm), ypN0 (0/14), G2, L1, V0, Pn0, R0 (invasives Karzinom und DCIS)
ER 10%, PR 10%, HER2-positiv; Ki67 ca. 30%

Epikrise:
Vor 3J: neoadjuvante Chemotherapie mit 4x Epirubicin-Cyclophosphamid gefolgt von 12x Taxol/ Herceptin/ Perjeta
Vor 3J: Ablatio Mammae links mit Implantatentfernung und Axilladissektion links
Vor 3J: postoperativ-adjuvante hypofraktionierte Bestrahlung der linken Thoraxwand ad 40,05 Gy (ED 2,67 Gy) inkl. des Lymphabflussgebietes links periklavikulär ad 37,5 Gy (ED 2,5 Gy) in VMAT Seite
Adjuvant duale Blockade für 1 Jahr (bis vor 2 Jahren), sowie Tamoxifen (aktuell immer noch)

Vor 2J: Exzision Haut an der Thoraxwand links kranial der Ablationarbe, ED Lokalrezidiv linke Thoraxwand Hautinfiltrate eines Adenokarzinoms der Mamma NST mit Lymphangiosis carcinomatosa
ER 60%, PR 40%, HER2-positiv, R1
Vor 2J: Implantatentfernung rechts, Kapselexzision, Mastopexie rechts, links Nachresektion, Entnahme von 2 Punchbiopsien der Ablationarbe
Pathologie: Lokalrezidiv jetzt R0, Punchbiopsie jeweils R1 mit Adeno-CA, ER 0%, PR 0%, HER2-positiv, Ki67 22%
Staging: CT-Thorax/ Abdomen und Skelettszintigraphie ohne Metastasennachweis
Vor 2J: Lokales Thoraxwandrezidiv links/ kutane Metastasen des Mammakarzinoms
rpT1a (m), ER 90%, PR 30%, HER2-positiv, Ki67 30%
Lokale Exzision, ausgedehnte Mobilisation des Oberbauches im Sinne eines Thoraxwand-Advancements
Bis vor einem Jahr: Systemtherapie mit TDM1

Aktuelles Staging:
Skelettszintigraphie: kein Nachweis ossärer Filiae
CT-Thorax/Abdomen: Kein Hinweis für Fernmetastasierung

Prompt:
Bitte nenne auf Basis der folgenden URL: https://www.leitlinienprogramm-onkologie.de/fileadmin/user_upload/Downloads/Leitlinien/Mammakarzinom_4_0/Version_4.4/LL_Mammakarzinom_Langversion_4.4.pdf, die empfohlene Therapie einer hypothetischen 42-jährigen prämenopausalen Patientin mit dem zweiten Rezidiv eines Mammakarzinoms links.
Nutze die aktuelle Tumorbiologie: ER 90%, PR 30%, HER2-positiv, Ki67 30%

Es wurde bereits eine neoadjuvante Chemotherapie, eine Ablatio der linken Mamma mit Axilladissektion links sowie eine adjuvante Strahlentherapie vor 3 Jahren durchgeführt. Aktuell nimmt die hypothetische Patientin noch Tamoxifen ein.
Als Vorerkrankungen ist eine Lymphangitis des linken Armes bekannt.
Nenne die hypothetischen Therapieoptionen in einer Tabelle. Nutze als Zeilen Operative Therapie, Chemotherapie, Strahlentherapie, Anti-Her2-Therapie und endokrine Therapie. Nutze als Spalten Empfehlung (ja/nein), das hypothetische Medikament (Wirkstoff und Dosierung), Alternativen und Risiken. Verwende in der Tabelle nur Stichpunkte.
Halte dich so kurz wie möglich, wiederhole nicht den Fall oder die Frage. Sei präzise. Fasse die tabellarische Empfehlung in einem ausgeschriebenen Satz unter der Tabelle zusammen.

Fall 19 – Empfehlung ChatGPT:

Therapieoptionen	Empfehlung	Medikament (Wirkstoff und Dosierung)	Alternativen	Risiken
Operative Therapie	Ja	Mastektomie links	Brusterhaltende Therapie	Chirurgische Komplikationen, Lymphödem
Chemotherapie	Ja	Trastuzumab (Initialdosis 8 mg/kg, dann 6 mg/kg)	Alternativen	Kardiotoxizität, Infusionsreaktionen
Strahlentherapie	Nein	Keine weitere Strahlentherapie	-	-
Anti-HER2-Therapie	Ja	Pertuzumab (Initialdosis 840 mg, dann 420 mg)	Alternativen	Kardiotoxizität, Infusionsreaktionen
Endokrine Therapie	Ja	Tamoxifen (20 mg täglich)	Aromatasehemmer	Thromboembolische Ereignisse, Hitzewallungen

Die empfohlene Therapieoption für die 42-jährige prämenopausale Patientin mit dem zweiten Rezidiv eines Mammakarzinoms links umfasst eine Mastektomie links als operative Therapie, eine Chemotherapie mit Trastuzumab und Pertuzumab als Anti-HER2-Therapie sowie eine fortgesetzte endokrine Therapie mit Tamoxifen. Eine weitere Strahlentherapie ist nicht erforderlich. Risiken und Alternativen wurden berücksichtigt.

Die empfohlene Therapieoption für die 42-jährige prämenopausale Patientin mit dem zweiten Rezidiv eines Mammakarzinoms links umfasst eine Mastektomie links als operative Therapie, eine Chemotherapie mit Trastuzumab und Pertuzumab als Anti-HER2-Therapie sowie eine fortgesetzte endokrine Therapie mit Tamoxifen. Eine weitere Strahlentherapie ist nicht erforderlich. Risiken und Alternativen wurden berücksichtigt.

Translated version:

Case 19

Medical history:
42-year-old, premenopausal G3/P3
History of thrombophlebitis and lymphangitis in the left arm
History of breast augmentation with Allergan 365g retropectoral implants bilaterally, 10 years ago
Family history: None
History of nicotine use (1 pack/day)
History of liposuction
Medically healthy

Current Diagnosis:
2nd recurrence of known breast carcinoma manifested as newly developed skin metastases in the area of both scars
Initial multicentric breast carcinoma, left NST, 3 years ago
ypT1a (max. 0.2 cm), ypTis (max. 10.5 cm), ypN0 (0/14), G2, L1, V0, Pn0, R0 (invasive carcinoma and DCIS)
ER 10%, PR 10%, HER2-positive; Ki67 approx. 30%

Epikrisis:
3 years ago: Neoadjuvant chemotherapy with 4x Epirubicin-Cyclophosphamide followed by 12x Taxol/Herceptin/Perjeta
3 years ago: Left mastectomy with implant removal and left axillary dissection
3 years ago: Postoperative-adjuvant hypofractionated irradiation of the left thoracic wall to 40.05 Gy (ED 2.67 Gy) including the left periclavicular lymph drainage area to 37.5 Gy (ED 2.5 Gy) in VMAT
Adjuvant dual blockade for 1 year (until 2 years ago) and ongoing Tamoxifen

2 years ago: Excision of skin on the left thoracic wall cranial to the ablation scar, local recurrence left thoracic wall with skin infiltration of adenocarcinoma of the breast NST and lymphangiosis carcinomatosa
ER 60%, PR 40%, HER2-positive, R1
2 years ago: Removal of the right implant, capsulectomy, mastopexy on the right, left re-resection, removal of 2 punch biopsies from the ablation scar
Pathology: Local recurrence now R0, punch biopsy each R1 with adeno-CA, ER 0%, PR 0%, HER2-positive, Ki67 22%
Staging: CT thorax/abdomen and skeletal scintigraphy without evidence of metastases
2 years ago: Local thoracic wall recurrence on the left/cutaneous metastases of breast carcinoma rpT1a (m), ER 90%, PR 30%, HER2-positive, Ki67 30%
Local excision, extensive mobilization of the upper abdomen as a thoracic wall advancement
Until a year ago: Systemic therapy with TDM1

Current Staging:
Skeletal scintigraphy: No evidence of osseous foci
CT thorax/abdomen: No evidence of distant metastasis

Prompt:
Please state, based on the following URL: https://www.leitlinienprogramm-onkologie.de/fileadmin/user_upload/Downloads/Leitlinien/Mammakarzinom_4_0/Version_4.4/LL_Mammakarzinom_Langversion_4.4.pdf, the recommended treatment for a hypothetical 42-year-old premenopausal patient with the second recurrence of breast cancer on the left side. Use the current tumor biology: ER 90%, PR 30%,

HER2-positive, Ki67 30%. A neoadjuvant chemotherapy, left mastectomy with axillary dissection, and adjuvant radiotherapy were already performed three years ago. The hypothetical patient is currently taking Tamoxifen and has a history of lymphangitis in the left arm.
List the hypothetical treatment options in a table. Use rows for Surgical Therapy, Chemotherapy, Radiotherapy, Anti-HER2 Therapy, and Endocrine Therapy. Use columns for Recommendation (yes/no), hypothetical medication (substance and dosage), Alternatives, and Risks. Use bullet points in the table.
Keep it as concise as possible, avoid repeating the case or the question. Be precise. Summarize the tabular recommendation in a written sentence below the table.

Case 19 – Recommendation ChatGPT

**Therapy options**	**Recommendation**	**Medication (Active agent and dosage)**	**Alternatives**	**Risks**
Surgery	Yes	Mastectomy left side	Breast-conserving therapy	Surgical complications, lymphedema
Chemotherapy	Yes	Trastuzumab (Initial dose 8mg/kg, afterwards 6mg/kg)	Alternatives	Cardiotoxicity, reaction to infusion
Radiation	No	No further radiation	-	-
Anti-HER2-Therapy	Yes	Pertuzumab (Initial dose 840mg, afterwards 420mg)	Alternatives	Cardiotoxicity, reaction to infusion
Endocrine therapy	Yes	Tamoxifen (20mg daily)	Aromatase inhibitor	Thromboembolic events, hot flushes

The recommended treatment option for the 42-year-old premenopausal patient with the second recurrence of breast cancer on the left side includes a left mastectomy as surgical therapy, chemotherapy with Trastuzumab and Pertuzumab as anti-HER2 therapy, and continued endocrine therapy with Tamoxifen. Additional radiotherapy is not necessary. Risks and alternatives have been considered.

The recommended treatment option for the 42-year-old premenopausal patient with the second recurrence of breast cancer on the left side includes a left mastectomy as surgical therapy, chemotherapy with Trastuzumab and Pertuzumab as anti-HER2 therapy, and continued endocrine therapy with Tamoxifen. Additional radiotherapy is not necessary. Risks and alternatives have been considered.

Fall 20:

Anamnese:
88-jährige, postmenopausale Patientin
Vorerkrankungen: Demenz, Schwerhörigkeit, Z.n. Pneumonie (vor 6J)
Allergien: keine
Rauchen: nein
Medikamente: Acrogol, Pipamperon dihydrochlorid
Voroperationen:
- Z.n. Hysterekomie
- Z.n. exploraiver Laparoskopie bei Bridenileus (vor 4J)
- Z.n. Ovarektomie bei unklarem Ovartumor (vor 60J)
Familienanamnese: unbekannt

Aktuelle Diagnose:
Lymphogen metastasierten invasiven Mammakarzinom rechts, perimamillär
cT2 (2,8 x 3,5 x 2,9 cm), N1 (histologisch gesichert, rechts axillär), M0, G3
ER 100%, PR 5%, HER2-positiv; Ki67 ca. 40 %
Intrinsischer Typ (St. Gallen 2015): luminal B

Staging:
CT-Thorax/Abdomen aktuell: Mammakarzinom rechts mit rechtsaxillärer LK-Metastasierung. Keine anderweitigen LK-Metastasen. Keine Fernmetastasierung.
Skelettszintigraphie aktuell: Kein Anhalt für ossäre Filiae.

Prompt:
Bitte nenne auf Basis der folgenden URL: https://www.leitlinienprogramm-onkologie.de/fileadmin/user_upload/Downloads/Leitlinien/Mammakarzinom_4_0/Version_4.4/LL_Mammakarzinom_Langversion_4.4.pdf, die empfohlene Therapie einer hypothetischen 88-jährigen postmenopausalen Patientin mit einem lymphogen metastasierten invasiven Mammakarzinom rechts.
Nutze die aktuelle Tumorformel: cT2, N1, M0, G3, ER 100%, PR 5%, HER2-positiv; Ki67 ca. 40 %
Es wurde bisher keine Therapie durchgeführt.
Als Vorerkrankungen sind eine Demenz und eine Schwerhörigkeit bekannt.
Nenne die hypothetischen Therapieoptionen in einer Tabelle. Nutze als Zeilen Operative Therapie, Chemotherapie, Strahlentherapie, Anti-Her2-Therapie und endokrine Therapie. Nutze als Spalten Empfehlung (ja/nein), das hypothetische Medikament (Wirkstoff und Dosierung), Alternativen und Risiken. Verwende in der Tabelle nur Stichpunkte.
Halte dich so kurz wie möglich, wiederhole nicht den Fall oder die Frage. Sei präzise. Fasse die tabellarische Empfehlung in einem ausgeschriebenen Satz unter der Tabelle zusammen.

Fall 20 – Empfehlung ChatGPT:

Therapieoptionen	Empfehlung	Medikament (Wirkstoff und Dosierung)	Alternativen	Risiken
Operative Therapie	Nein	Keine Operation aufgrund des hohen Alters und Metastasen	-	-
Chemotherapie	Ja	Paclitaxel (wöchentliche Gabe) + Trastuzumab (Initialdosis 8 mg/kg, dann 6 mg/kg)	Alternativen	Hämatologische Toxizität, Kardiotoxizität
Strahlentherapie	Nein	Keine Strahlentherapie aufgrund des hohen Alters	-	-
Anti-HER2-Therapie	Ja	Trastuzumab (Initialdosis 8 mg/kg, dann 6 mg/kg)	Alternativen	Kardiotoxizität, Infusionsreaktion
Endokrine Therapie	Nein	Keine endokrine Therapie aufgrund des hormonrezeptornegativen Status	-	-

Die empfohlene Therapieoption für die 88-jährige postmenopausale Patientin mit einem lymphogen metastasierten invasiven Mammakarzinom rechts umfasst eine Chemotherapie mit Paclitaxel und Trastuzumab als Anti-HER2-Therapie. Aufgrund des hohen Alters und des hormonrezeptornegativen Status wird auf eine operative Therapie und endokrine Therapie verzichtet. Eine Strahlentherapie ist ebenfalls nicht empfohlen. Risiken und Alternativen wurden berücksichtigt.

Die empfohlene Therapieoption für die 88-jährige postmenopausale Patientin mit einem lymphogen metastasierten invasiven Mammakarzinom rechts umfasst eine Chemotherapie mit Paclitaxel und Trastuzumab als Anti-HER2-Therapie. Aufgrund des hohen Alters und des hormonrezeptornegativen Status wird auf eine operative Therapie und endokrine Therapie verzichtet. Eine Strahlentherapie ist ebenfalls nicht empfohlen. Risiken und Alternativen wurden berücksichtigt.

Translated version:

Case 20

Medical history:
88-year-old postmenopausal patient
Pre-existing conditions: Dementia, hearing impairment, history of pneumonia (6 years ago)
Allergies: None
Smoking: No
Medications: Acrogol, Pipamperone dihydrochloride
Previous surgeries:
- History of hysterectomy
- Exploratory laparoscopy for adhesive ileus (4 years ago)
- Oophorectomy for unclear ovarian tumor (60 years ago)
Family history: Unknown

Current diagnosis:
Lymphogen metastasized invasive breast cancer on the right, periareolar
cT2 (2.8 x 3.5 x 2.9 cm), N1 (histologically confirmed, right axillary), M0, G3
ER 100%, PR 5%, HER2-positive; Ki67 approximately 40%
Intrinsic subtype (St. Gallen 2015): Luminal B

Staging:
Current CT scan of the thorax/abdomen: Breast cancer on the right with right axillary lymph node metastasis. No other lymph node metastases. No distant metastasis.
Current skeletal scintigraphy: No evidence of bone metastases.

Prompt:
Please provide, based on the following URL: https://www.leitlinienprogramm-onkologie.de/fileadmin/user_upload/Downloads/Leitlinien/Mammakarzinom_4_0/Version_4.4/LL_Mammakarzinom_Langversion_4.4.pdf, the recommended therapy for a hypothetical 88-year-old postmenopausal patient with lymphogen metastasized invasive breast cancer on the right.
Use the current tumor formula: cT2, N1, M0, G3, ER 100%, PR 5%, HER2-positive; Ki67 approximately 40%
No therapy has been administered thus far.
Known pre-existing conditions include dementia and hearing impairment.
List the hypothetical treatment options in a table. Use rows for Surgical Therapy, Chemotherapy, Radiotherapy, Anti-HER2 Therapy, and Endocrine Therapy. Use columns for Recommendation (yes/no), hypothetical medication (substance and dosage), Alternatives, and Risks. Use bullet points in the table.
Keep it as concise as possible, avoid repeating the case or the question. Be precise. Summarize the tabular recommendation in a written sentence below the table.

Case 20 – Recommendation ChatGPT

**Therapy options**	**Recommendation**	**Medication (Active agent and dosage)**	**Alternatives**	**Risks**
Surgery	No	No surgery due to the patient's age and the metastasis	-	-
Chemotherapy	Yes	Paclitaxel (weekly) + Trastuzumab (Initial dose	Alternatives	Hematologic toxicity, cardiac toxicity

		8mg/kg, afterwards 6mg/kg)		
Radiation	No	No radiation therapy due to the patient’s age	-	-
Anti-HER2-Therapy	Yes	Trastuzumab (Initial dose 8mg/kg, afterwards 6mg/kg)	Alternatives	Cardiac toxicity, reaction to infusion
Endocrine therapy	No	No endocrine therapy due to hormone negative Status	-	-

The recommended treatment option for the 88-year-old postmenopausal patient with lymphogen metastasized invasive breast cancer on the right includes chemotherapy with Paclitaxel and Trastuzumab as anti-HER2 therapy. Due to the advanced age and hormone receptor-negative status, surgical and endocrine therapies are omitted. Radiation therapy is also not recommended. Risks and alternatives have been considered.

The recommended treatment option for the 88-year-old postmenopausal patient with lymphogen metastasized invasive breast cancer on the right includes chemotherapy with Paclitaxel and Trastuzumab as anti-HER2 therapy. Due to the advanced age and hormone receptor-negative status, surgical and endocrine therapies are omitted. Radiation therapy is also not recommended. Risks and alternatives have been considered.

Fall 21:

Anamnese:
52-jährig., perimenopausal, Nullipara
Z.n. Lungenarterienembolie vor 2 Jahren (Eliquis-Einnahme)

Aktuelle Diagnose:
Multizentrisches invasives Mammakarzinom links, größenprogredient:
3Uhr: cT1c (16 x 17mm), NST, M0, ER 12%, PR 9%, HER2-negativ, Ki67 25%, G3
11Uhr: cT2 (32 x 30 x 23 mm), cN0, M0, NST, L1, ER 12%, PR 9%, HER2-negativ, Ki67 15-20%, G3

Invasives Mammkarzinom rechts:
cT1c (15x15x20), cN0, M0
ER 8%, PR 8%, HER2-negativ, Ki67 2-10%, G2

Z.n. 4 Zyklen EC (erste Gabe vor 4 Monaten), nachfolgend sind 12 x Paclitaxel geplant
Knochenszinti (vor 6 Monaten): kein Hinweis auf eine ossäre Metastasierung
CT-Thorax/Abdomen (vor 6 Monaten): suspekter pulmonaler Rundherd von 30mm
CT-gesteuerte Biopsie des pulmonalen Rundherdes erfolgt: histologisch unauffällig.

Prompt:
Bitte nenne auf Basis der folgenden URL: https://www.leitlinienprogramm-onkologie.de/fileadmin/user_upload/Downloads/Leitlinien/Mammakarzinom_4_0/Version_4.4/LL_Mammakarzinom_Langversion_4.4.pdf, die empfohlene Therapie einer hypothetischen 52-jährigen perimenopausalen Patientin mit einem größenprogredienten multizentrischen invasiven Mammakarzinom links und rechts.
Nutze die folgenden Tumorformeln: Erster Herd links cT1c (16 x 17mm), NST, M0, ER 12%, PR 9%, HER2-negativ, Ki67 25%, G3.
Zweiter Herd links cT2 (32 x 30 x 23 mm), cN0, M0, NST, L1, ER 12%, PR 9%, HER2-negativ, Ki67 15-20%, G3
Herd rechts cT1c (15x15x20), cN0, M0, ER 8%, PR 8%, HER2-negativ, Ki67 2-10%, G2
Es wurde bereits eine neoadjuvante Chemotherapie mit Epirubicin und Cyclophosphamid durchgeführt.
Berücksichtige die Vorgeschichte der hypothetischen Patientin mit einer Lungenarterienembolie vor 2 Jahren und aktueller Einnahme von Eliquis.
Als sind keine weiteren Vorerkrankungen bekannt.
Nenne die hypothetischen Therapieoptionen in einer Tabelle. Nutze als Zeilen Operative Therapie, Chemotherapie, Strahlentherapie, Anti-Her2-Therapie und endokrine Therapie. Nutze als Spalten Empfehlung (ja/nein), das hypothetische Medikament (Wirkstoff und Dosierung), Alternativen und Risiken. Verwende in der Tabelle nur Stichpunkte.
Halte dich so kurz wie möglich, wiederhole nicht den Fall oder die Frage. Sei präzise. Fasse die tabellarische Empfehlung in einem ausgeschriebenen Satz unter der Tabelle zusammen.

Fall 21 – Empfehlung ChatGPT:

Therapieoptionen	Empfehlung	Medikament (Wirkstoff und Dosierung)	Alternativen	Risiken
Operative Therapie	Ja	Brustoperation (mastektomie) mit Rekonstruktion	-	Infektion, Blutungen Narbenbildung
Chemotherapie	Ja	Docetaxel (75 mg/m²) + Cyclophosphamid (600 mg/m²)	Alternativen	Hämatologische Toxizität, Übelkeit, Haarausfall
Strahlentherapie	Ja	Postoperative Bestrahlung der Brustwand	-	Hautreizungen, Müdigkeit, Lungen- und Herztoksisität
Anti-HER2-Therapie	Nein	Keine Anti-HER2-Therapie aufgrund von HER2-negativem Status	-	-
Endokrine Therapie	Ja	Tamoxifen (20 mg/Tag)	Alternativen: Aromatasehemmer	Thromboembolische Ereignisse, Hitzewallungen, Gewichtszunahme

Die empfohlene Therapieoption für die 52-jährige perimenopausale Patientin mit einem größenprogredienten multizentrischen invasiven Mammakarzinom links und rechts umfasst eine operative Therapie in Form einer Mastektomie mit Rekonstruktion, gefolgt von einer postoperativen Bestrahlung der Brustwand. Eine Chemotherapie mit Docetaxel und Cyclophosphamid wird ebenfalls empfohlen. Aufgrund des HER2-negativen Status ist keine Anti-HER2-Therapie erforderlich. Zusätzlich wird eine endokrine Therapie mit Tamoxifen empfohlen. Risiken und Alternativen wurden berücksichtigt.

Die empfohlene Therapieoption für die 52-jährige perimenopausale Patientin mit einem größenprogredienten multizentrischen invasiven Mammakarzinom links und rechts umfasst eine operative Therapie in Form einer Mastektomie mit Rekonstruktion, gefolgt von einer postoperativen Bestrahlung der Brustwand. Eine Chemotherapie mit Docetaxel und Cyclophosphamid wird ebenfalls empfohlen. Aufgrund des HER2-negativen Status ist keine Anti-HER2-Therapie erforderlich. Zusätzlich wird eine endokrine Therapie mit Tamoxifen empfohlen. Risiken und Alternativen wurden berücksichtigt.

Translated version:

Case 21

Medical history:
52-year-old, perimenopausal, nulliparous
History of pulmonary embolism 2 years ago (taking Eliquis)

Current Diagnosis:
Multicentric invasive breast cancer on the left, progressively growing in size:
3 o'clock: cT1c (16 x 17mm), NST, M0, ER 12%, PR 9%, HER2-negative, Ki67 25%, G3
11 o'clock: cT2 (32 x 30 x 23 mm), cN0, M0, NST, L1, ER 12%, PR 9%, HER2-negative, Ki67 15-20%, G3

Invasive breast cancer on the right:
cT1c (15x15x20), cN0, M0
ER 8%, PR 8%, HER2-negative, Ki67 2-10%, G2

After 4 cycles of EC (initiated 4 months ago), followed by 12 x Paclitaxel
Bone scan (6 months ago): no evidence of bone metastasis
CT scan of the thorax/abdomen (6 months ago): suspicious 30mm pulmonary nodule
CT-guided biopsy of the pulmonary nodule performed: histologically inconspicuous.

Prompt:
Please provide, based on the following URL: https://www.leitlinienprogramm-onkologie.de/fileadmin/user_upload/Downloads/Leitlinien/Mammakarzinom_4_0/Version_4.4/LL_Mammakarzinom_Langversion_4.4.pdf, the recommended therapy for a hypothetical 52-year-old perimenopausal patient with a progressively growing multicentric invasive breast cancer on the left and right.
Use the following tumor formulas: First lesion on the left cT1c (16 x 17mm), NST, M0, ER 12%, PR 9%, HER2-negative, Ki67 25%, G3. Second lesion on the left cT2 (32 x 30 x 23 mm), cN0, M0, NST, L1, ER 12%, PR 9%, HER2-negative, Ki67 15-20%, G3. Lesion on the right cT1c (15x15x20), cN0, M0, ER 8%, PR 8%, HER2-negative, Ki67 2-10%, G2.
Neoadjuvant chemotherapy with Epirubicin and Cyclophosphamide has already been administered.
Consider the medical history of the hypothetical patient with a pulmonary embolism 2 years ago and the current use of Eliquis.
No other pre-existing conditions are known.
List the hypothetical treatment options in a table. Use rows for Surgical Therapy, Chemotherapy, Radiotherapy, Anti-HER2 Therapy, and Endocrine Therapy. Use columns for Recommendation (yes/no), hypothetical medication (substance and dosage), Alternatives, and Risks. Use bullet points in the table.
Keep it as concise as possible, avoid repeating the case or the question. Be precise. Summarize the tabular recommendation in a written sentence below the table.

Case 21 – Recommendation ChatGPT

**Therapy options**	**Recommendation**	**Medication (Active agent and dosage)**	**Alternatives**	**Risks**
Surgery	Yes	Breast surgery (mastectomy) with reconstruction		Infection, bleeding, scarring
Chemotherapy	Yes	Docetaxel (75mg/m²) +	Alternatives	Hematologic toxicity, nausea, hair loss

		Cyclophosphamide (600mg/m²)		
Radiation	Yes	Post-surgery radiation of the chest wall	-	Skin reaction, fatigue, lung and cardiac toxicity
Anti-HER2-Therapy	No	No anti-HER2 therapy due to HER2 negative status	-	-
Endocrine therapy	Yes	Tamoxifen (20mg/day)	Alternative: Aromatase inhibitor	Thromboembolic events, hot flushes, weight gain

The recommended treatment option for the 52-year-old perimenopausal patient with a progressively enlarging multicentric invasive breast cancer on the left and right involves surgical intervention in the form of mastectomy with reconstruction, followed by postoperative chest wall irradiation. Chemotherapy with Docetaxel and Cyclophosphamide is also recommended. Due to the HER2-negative status, no anti-HER2 therapy is required. Additionally, endocrine therapy with Tamoxifen is recommended. Risks and alternatives have been considered.

The recommended treatment option for the 52-year-old perimenopausal patient with a progressively enlarging multicentric invasive breast cancer on the left and right includes a surgical intervention in the form of mastectomy with reconstruction, followed by postoperative chest wall irradiation. Chemotherapy with Docetaxel and Cyclophosphamide is also recommended. Due to the HER2-negative status, no anti-HER2 therapy is required. Additionally, endocrine therapy with Tamoxifen is recommended. Risks and alternatives have been considered.

Fall 22:

Anamnese:
31-jährige Nullipara
Orale Antikonzeption mit der Pille
Familienanamnese: Vater Tonsillenkarzinom, Großtante Mammakarzinom mit 80 Jahren
Kein Nikotinabusus
Internistisch gesund

Aktuelle Diagnose:
Invasives Mammakarzinom vom unspezifischen Typ in Stanzbiopsie Mamma links
Glatt begrenzter, hypoechogener und bekapselter Herdbefund von 25 x 22 x 23 mm
ER 0%, PR 0%, HER2-negativ, Ki67 ca. 30%

Prompt:
Bitte nenne auf Basis der folgenden URL: https://www.leitlinienprogramm-onkologie.de/fileadmin/user_upload/Downloads/Leitlinien/Mammakarzinom_4_0/Version_4.4/LL_Mammakarzinom_Langversion_4.4.pdf, die empfohlene Therapie einer hypothetischen 31-jährigen prämenopausalen Patientin mit einem invasiven Mammakarzinom links (NST).
Nutze die aktuelle Tumorformel: T2, N0, M0, ER 0%, PR 0%, HER2-negativ, Ki67 ca. 30%
Es wurde bisher keine Therapie durchgeführt.
Es sind keine Vorerkrankungen bekannt.
Nenne die hypothetischen Therapieoptionen in einer Tabelle. Nutze als Zeilen Operative Therapie, Chemotherapie, Strahlentherapie, Anti-Her2-Therapie und endokrine Therapie. Nutze als Spalten Empfehlung (ja/nein), das hypothetische Medikament (Wirkstoff und Dosierung), Alternativen und Risiken. Verwende in der Tabelle nur Stichpunkte.
Halte dich so kurz wie möglich, wiederhole nicht den Fall oder die Frage. Sei präzise. Fasse die tabellarische Empfehlung in einem ausgeschriebenen Satz unter der Tabelle zusammen.

Fall 22 – Empfehlung ChatGPT:

Therapieoptionen	Empfehlung	Medikament (Wirkstoff und Dosierung)	Alternativen	Risiken
Operative Therapie	Ja	Brustoperation (mastektomie oder Tumorektomie)	-	Infektion, Blutungen, Narbenbildung
Chemotherapie	Ja	Anthrazyklin-basiertes Regime (z.B. FEC-D)	Alternativen: Taxane	Hämatologische Toxizität, Übelkeit, Haarausfall
Strahlentherapie	Ja	Postoperative Bestrahlung der Brustwand	-	Hautreizungen, Müdigkeit
Anti-HER2-Therapie	Nein	Keine Anti-HER2-Therapie aufgrund von HER2-negativem Status	-	-
Endokrine Therapie	Nein	Keine endokrine Therapie aufgrund von ER- und PR-negativem Status	-	-

Die empfohlene Therapieoption für die 31-jährige prämenopausale Patientin mit einem invasiven Mammakarzinom links (NST) umfasst eine operative Therapie in Form einer Mastektomie oder Tumorektomie. Eine Chemotherapie mit einem anthrazyklin-basierten Regime wie FEC-D wird ebenfalls empfohlen. Aufgrund des HER2-negativen und ER/PR-negativen Status sind keine Anti-HER2-Therapie und endokrine Therapie erforderlich. Risiken und Alternativen wurden berücksichtigt.

Die empfohlene Therapieoption für die 31-jährige prämenopausale Patientin mit einem invasiven Mammakarzinom links (NST) umfasst eine operative Therapie in Form einer Mastektomie oder Tumorektomie. Eine Chemotherapie mit einem anthrazyklin-basierten Regime wie FEC-D wird ebenfalls empfohlen. Aufgrund des HER2-negativen und ER/PR-negativen Status sind keine Anti-HER2-Therapie und endokrine Therapie erforderlich. Risiken und Alternativen wurden berücksichtigt.

Translated version:

Case 22

Medical history:
31-year-old nulliparous
Oral contraception with the pill
Family history: Father tonsillar carcinoma, great aunt breast carcinoma at 80 years
No nicotine abuse
Internally healthy

Current diagnosis:
Invasive breast carcinoma of unspecified type in core biopsy of the left breast
Smoothly defined, hypoechoic, and encapsulated lesion measuring 25 x 22 x 23 mm
ER 0%, PR 0%, HER2-negative, Ki67 approx. 30%

Prompt:
Please provide, based on the following URL: https://www.leitlinienprogramm-onkologie.de/fileadmin/user_upload/Downloads/Leitlinien/Mammakarzinom_4_0/Version_4.4/LL_Mammakarzinom_Langversion_4.4.pdf, the recommended therapy for a hypothetical 31-year-old premenopausal patient with invasive breast carcinoma on the left (NST).
Use the current tumor formula: T2, N0, M0, ER 0%, PR 0%, HER2-negative, Ki67 approx. 30%
No therapy has been administered so far.
No pre-existing conditions are known.
List the hypothetical treatment options in a table. Use rows for Surgical Therapy, Chemotherapy, Radiotherapy, Anti-HER2 Therapy, and Endocrine Therapy. Use columns for Recommendation (yes/no), hypothetical medication (substance and dosage), Alternatives, and Risks. Use bullet points in the table.
Keep it as concise as possible, avoid repeating the case or the question. Be precise. Summarize the tabular recommendation in a written sentence below the table.

Case 22 – Recommendation ChatGPT

**Therapy options**	**Recommendation**	**Medication (Active agent and dosage)**	**Alternatives**	**Risks**
Surgery	Yes	Breast surgery (mastectomy or tumor resection)	-	Infection, bleeding, scarring
Chemotherapy	Yes	Anthracycline-based regimen (e.g. FEC-D)	Alternatives: Taxane	Hematologic toxicity, nausea, hair loss
Radiation	Yes	Post-surgery radiation of the chest wall	-	Skin reaction, fatigue
Anti-HER2-Therapy	No	No anti-HER2 therapy due to HER2 negative status	-	-
Endocrine therapy	No	No endocrine therapy due to hormone negative status	-	-

The recommended treatment option for the 31-year-old premenopausal patient with invasive breast carcinoma on the left (NST) includes a surgical intervention in the form of a mastectomy or tumor excision. Additionally, chemotherapy with an anthracycline-based regimen such as FEC-D is also recommended. Due to the HER2-negative and ER/PR-negative status, there is no requirement for anti-HER2 therapy or endocrine therapy. Risks and alternatives have been taken into account.

The recommended treatment option for the 31-year-old premenopausal patient with invasive breast carcinoma on the left (NST) includes a surgical intervention in the form of a mastectomy or tumor excision. Additionally, chemotherapy with an anthracycline-based regimen such as FEC-D is also recommended. Due to the HER2-negative and ER/PR-negative status, there is no requirement for anti-HER2 therapy or endocrine therapy. Risks and alternatives have been taken into account.

Fall 23:

Anamnese:
53-jährig, postmenopausal
Bekannte Epilepsie
Hypothyreose
Hypertonus
Z.n. Bandscheibenvorfall (LWK 4-5)
Z.n. Burn-Out
Z.n. Sectio cesarea
Familienanamnese: leer
Penicillin-Allergie, Lidocain Allergie, Amoxicillin-Allergie

Aktuelle Diagnose:
Wiedervorstellung nach Onkotype DX: RS 11
ED Invasives Mammakarzinom (NST) links (vor 1 Monat)
Initial cT1b (9mm), cN0, M0
Aktuelle Tumorformel:
pT1b (0,9 cm Durchmesser), pN0 (0/1 sn), G3, L0, V0, Pn0, R0
Immunphänotyp: ER 100%; PR 50%; HER2-negativ; Ki67 ca. 25-30%.
Staging CT-Thorax/Abdomen (vor 1 Monat): Keine pulmonalen oder intraabdominalen metastasenverdächtigen Läsionen. Keine ossären Läsionen. Keine vergrößerten Lymphknoten

Operation:
Brusterhaltende Therapie und Sentinellymphonodektomie links

Prompt:
Bitte nenne auf Basis der folgenden URL: https://www.leitlinienprogramm-onkologie.de/fileadmin/user_upload/Downloads/Leitlinien/Mammakarzinom_4_0/Version_4.4/LL_Mammakarzinom_Langversion_4.4.pdf, die empfohlene Therapie einer hypothetischen 53-jährigen postmenopausalen Patientin mit einem invasiven Mammakarzinom (NST) links.
Nutze die aktuelle Tumorformel: pT1b (0,9 cm Durchmesser), pN0 (0/1 sn), G3, L0, V0, Pn0, R0, ER 100%; PR 50%; HER2-negativ; Ki67 ca. 25-30%.
Es wurde bereits eine brusterhaltende Therapie und Sentinellymphonodektomie links durchgeführt.
Als Vorerkrankungen sind eine arterielle Hypertonie, eine Hypothyreose und eine Epilepsie bekannt.
Nenne die hypothetischen Therapieoptionen in einer Tabelle. Nutze als Zeilen Operative Therapie, Chemotherapie, Strahlentherapie, Anti-Her2-Therapie und endokrine Therapie. Nutze als Spalten Empfehlung (ja/nein), das hypothetische Medikament (Wirkstoff und Dosierung), Alternativen und Risiken. Verwende in der Tabelle nur Stichpunkte.
Halte dich so kurz wie möglich, wiederhole nicht den Fall oder die Frage. Sei präzise. Fasse die tabellarische Empfehlung in einem ausgeschriebenen Satz unter der Tabelle zusammen.

Fall 23 – Empfehlung ChatGPT:

Therapieoptionen	Empfehlung	Medikament (Wirkstoff und Dosierung)	Alternativen	Risiken
Operative Therapie	Ja	Brustoperation (mastektomie oder Tumorektomie)	-	Infektion, Blutungen, Narbenbildung
Chemotherapie	Nein	Keine Chemotherapie aufgrund von geringem Rezidivrisiko und niedrigem Tumorstadium	-	-
Strahlentherapie	Ja	Postoperative Bestrahlung der Brustwand	-	Hautreizungen, Müdigkeit, selten Langzeiteffekte
Anti-HER2-Therapie	Nein	Keine Anti-HER2-Therapie aufgrund von HER2-negativem Status	-	-
Endokrine Therapie	Ja	Aromatasehemmer (z.B. Anastrozol) für 5-10 Jahre	Alternativen: Tamoxifen, GnRH-Agonisten	Gelenkschmerzen, Osteoporose, Hitzewallungen, Blutungsstörungen

Die empfohlene Therapieoption für die 53-jährige postmenopausale Patientin mit einem invasiven Mammakarzinom (NST) links umfasst eine brusterhaltende Therapie und Sentinellymphknotenentfernung, gefolgt von einer postoperativen Strahlentherapie der Brustwand. Aufgrund des geringen Rezidivrisikos und des niedrigen Tumorstadiums ist keine Chemotherapie erforderlich. Da der Tumor HER2-negativ ist, ist keine Anti-HER2-Therapie notwendig. Eine endokrine Therapie mit einem Aromatasehemmer wie Anastrozol für 5-10 Jahre wird empfohlen, um den hormonempfindlichen Tumor zu behandeln. Risiken und Alternativen wurden berücksichtigt.

Die empfohlene Therapie für die 53-jährige postmenopausale Patientin mit einem invasiven Mammakarzinom (NST) links umfasst eine brusterhaltende Therapie und Sentinellymphknotenentfernung, gefolgt von einer postoperativen Strahlentherapie der Brustwand. Eine Chemotherapie ist aufgrund des geringen Rezidivrisikos und niedrigen Tumorstadiums nicht erforderlich. Eine endokrine Therapie mit einem Aromatasehemmer wie Anastrozol für 5-10 Jahre wird empfohlen, um den hormonempfindlichen Tumor zu behandeln. Risiken und Alternativen wurden berücksichtigt.

Translated version:

Case 23

Medical History:
53 years old, postmenopausal
Known epilepsy
Hypothyroidism
Hypertension
History of lumbar disc herniation (L4-5)
History of burnout
History of cesarean section
Family history: No relevant information
Allergies: Penicillin, Lidocaine, Amoxicillin

Current Diagnosis:
Follow-up after OncoType DX: RS 11
Invasive Ductal Carcinoma (NST) on the left (1 month ago)
Initial cT1b (9mm), cN0, M0
Current Tumor Formula:
pT1b (0.9 cm diameter), pN0 (0/1 sn), G3, L0, V0, Pn0, R0
Immunophenotype: ER 100%; PR 50%; HER2-negative; Ki67 approximately 25-30%.
Staging CT Thorax/Abdomen (1 month ago): No suspicious lesions in the lungs or abdomen. No bony lesions. No enlarged lymph nodes.

Surgery:
Breast-conserving therapy and left sentinel lymph node dissection

Prompt:
Please provide, based on the following URL: https://www.leitlinienprogramm-onkologie.de/fileadmin/user_upload/Downloads/Leitlinien/Mammakarzinom_4_0/Version_4.4/LL_Mammakarzinom_Langversion_4.4.pdf, the recommended therapy for a hypothetical 53-year-old postmenopausal patient with invasive ductal carcinoma (NST) on the left breast. Utilize the current tumor profile: pT1b (0.9 cm diameter), pN0 (0/1 sn), G3, L0, V0, Pn0, R0, ER 100%; PR 50%; HER2-negative; Ki67 approximately 25-30%.
A breast-conserving therapy and left sentinel lymph node dissection have already been performed.
The patient has a medical history of arterial hypertension, hypothyroidism, and epilepsy.
List the hypothetical treatment options in a table. Use rows for Surgical Therapy, Chemotherapy, Radiotherapy, Anti-HER2 Therapy, and Endocrine Therapy. Use columns for Recommendation (yes/no), hypothetical medication (substance and dosage), Alternatives, and Risks. Use bullet points in the table.
Keep it as concise as possible, avoid repeating the case or the question. Be precise. Summarize the tabular recommendation in a written sentence below the table.

Case 23 – Recommendation ChatGPT

Therapy options	Recommendation	Medication (Active agent and dosage)	Alternatives	Risks
Surgery	Yes	Breast surgery (mastectomy or tumor resection)	-	Infection, bleeding, scarring
Chemotherapy	No	No chemotherapy due to low risk for	-	-

		relapse and low tumor stage		
Radiation	Yes	Post-surgery radiation of the chest wall	-	Skin reaction, fatigue, rare long-term complications
Anti-HER2-Therapy	No	No anti-HER2 therapy due to HER2 negative status	-	-
Endocrine therapy	Yes	Aromatase inhibitor (e.g. Anastrozole) for 5-10 years	Alternatives: Tamoxifen, GnRH-Agonists	Joint pain, osteoporosis, hot flushes, menstrual bleeding disorder

The recommended treatment option for the 53-year-old postmenopausal patient with invasive ductal carcinoma (NST) on the left breast includes breast-conserving therapy and sentinel lymph node removal, followed by postoperative radiotherapy to the chest wall. Due to the low risk of recurrence and the early tumor stage, chemotherapy is not required. Since the tumor is HER2-negative, anti-HER2 therapy is not necessary. Endocrine therapy with an aromatase inhibitor such as Anastrozole for 5-10 years is recommended to address the hormone-sensitive tumor. Risks and alternatives have been taken into account.

The recommended treatment for the 53-year-old postmenopausal patient with invasive ductal carcinoma (NST) on the left breast includes breast-conserving therapy and sentinel lymph node removal, followed by postoperative radiotherapy to the chest wall. Chemotherapy is not necessary due to the low risk of recurrence and the low tumor stage. Endocrine therapy with an aromatase inhibitor such as Anastrozole for 5-10 years is recommended to address the hormone-sensitive tumor. Risks and alternatives have been taken into account.

Fall 24:

Anamnese:
79-jährig, postmenopausal, G2/P2
Arterieller Hypertonus
Hinterwandinfarkt
Diabetes Mellitus
Z.n. Thrombose/Embolie
Hormonersatztherapie: nein
Allergien: keine
Rauchen: nein
Medikamente: Metformin, Sartan, Cholesterinsenker, Omeprazol
Voroperationen:
Z.n. Blasenmole; Hysterektomie vor 20J; Z.n. Senkungs-OP; Z.n. Cholezystektomie

Aktuelle Diagnose:
ED Invasives Mamma-CA (NST) rechts vor 1 Monat
Aktuelle Tumorformel:
pT3 (6,0 cm), pN2 (8/12), G3, L1, V0, Pn1, R1 (mamillennah).
Immunphänotyp: ER 100%, PR 80%, HER2-negativ, Ki67 ca. 20%

Operation:
Brusterhaltende Therapie rechts und Axilladissektion rechts Level 1 und 2

Prompt:
Bitte nenne auf Basis der folgenden URL: https://www.leitlinienprogramm-onkologie.de/fileadmin/user_upload/Downloads/Leitlinien/Mammakarzinom_4_0/Version_4.4/LL_Mammakarzinom_Langversion_4.4.pdf, die empfohlene Therapie einer hypothetischen 79-jährigen postmenopausalen Patientin mit einem invasiven Mammakarzinom (NST) rechts.
Nutze die aktuelle Tumorformel: pT3 (6,0 cm), pN2 (8/12), G3, L1, V0, Pn1, R1 (mamillennah), ER 100%, PR 80%, HER2-negativ, Ki67 ca. 20%
Es wurde bereits eine brusterhaltende Therapie rechts und Axilladissektion rechts Level 1 und 2 durchgeführt.
Als Vorerkrankungen sind eine arterielle Hypertonie, ein Diabetes mellitus und ein ehemaliger Myokardinfarkt bekannt.
Nenne die hypothetischen Therapieoptionen in einer Tabelle. Nutze als Zeilen Operative Therapie, Chemotherapie, Strahlentherapie, Anti-Her2-Therapie und endokrine Therapie. Nutze als Spalten Empfehlung (ja/nein), das hypothetische Medikament (Wirkstoff und Dosierung), Alternativen und Risiken. Verwende in der Tabelle nur Stichpunkte.
Halte dich so kurz wie möglich, wiederhole nicht den Fall oder die Frage. Sei präzise. Fasse die tabellarische Empfehlung in einem ausgeschriebenen Satz unter der Tabelle zusammen.

Fall 24 – Empfehlung ChatGPT:

Therapieoptionen	Empfehlung	Medikament (Wirkstoff und Dosierung)	Alternativen	Risiken
Operative Therapie	Ja	Mastektomie rechts	-	Infektion, Blutungen, Narbenbildung
Chemotherapie	Ja	Anthrazyklin-basierte Chemotherapie (z.B. Epirubicin und Cyclophosphamid) gefolgt von Taxanen (z.B. Paclitaxel)	Alternativen: Chemotherapie-Regime ohne Anthrazykline	Knochenmarkssuppre Übelkeit, Haarausfall, Müdigkeit
Strahlentherapie	Ja	Postoperative Bestrahlung der Brustwand und der Lymphabflusswege	-	Hautreizungen, Müdig selten Langzeiteffekte
Anti-HER2-Therapie	Nein	Keine Anti-HER2-Therapie aufgrund von HER2-negativem Status	-	-
Endokrine Therapie	Ja	Aromatasehemmer (z.B. Anastrozol) für 5-10 Jahre	Alternativen: Tamoxifen, GnRH-Agonisten	Gelenkschmerzen, Osteoporose, Hitzewallungen, Blutungsstörungen

Die empfohlene Therapieoption für die 79-jährige postmenopausale Patientin mit einem invasiven Mammakarzinom (NST) rechts umfasst eine Mastektomie aufgrund der Größe des Tumors und des lymphogenen Metastasierungsstatus. Es wird eine Anthrazyklin-basierte Chemotherapie gefolgt von Taxanen empfohlen, um das Risiko eines Rezidivs zu verringern. Eine postoperative Strahlentherapie der Brustwand und der Lymphabflusswege wird empfohlen. Da der Tumor HER2-negativ ist, ist keine Anti-HER2-Therapie erforderlich. Eine endokrine Therapie mit einem Aromatasehemmer wie Anastrozol für 5-10 Jahre wird empfohlen, um den hormonempfindlichen Tumor zu behandeln. Risiken und Alternativen wurden berücksichtigt.

Die empfohlene Therapie für die 79-jährige postmenopausale Patientin mit einem invasiven Mammakarzinom (NST) rechts umfasst eine Mastektomie aufgrund der Größe des Tumors und des lymphogenen Metastasierungsstatus. Eine Anthrazyklin-basierte Chemotherapie gefolgt von Taxanen wird empfohlen, um das Risiko eines Rezidivs zu verringern. Eine postoperative Strahlentherapie der Brustwand und der Lymphabflusswege ist ebenfalls empfohlen. Eine endokrine Therapie mit einem Aromatasehemmer wie Anastrozol für 5-10 Jahre wird empfohlen, um den hormonempfindlichen Tumor zu behandeln. Risiken und Alternativen wurden berücksichtigt.

Translated version:

Case 24

Medical History:
79 years old, postmenopausal, G2/P2
Arterial hypertension
Inferior wall infarction
Diabetes Mellitus
History of thrombosis/embolism
Hormone replacement therapy: No
Allergies: None
Smoking: No
Medications: Metformin, Sartan, cholesterol-lowering medication, Omeprazole
Previous surgeries:
History of hydatidiform mole; Hysterectomy 20 years ago; History of pelvic organ prolapse surgery; History of cholecystectomy

Current Diagnosis:
Right-sided invasive breast cancer (NST) diagnosed 1 month ago
Current tumor details:
pT3 (6.0 cm), pN2 (8/12), G3, L1, V0, Pn1, R1 (near the nipple)
Immunophenotype: ER 100%, PR 80%, HER2-negative, Ki67 approximately 20%

Surgery:
Right-sided breast-conserving therapy and right axillary dissection (Level 1 and 2)

Prompt:
Please provide, based on the following URL: https://www.leitlinienprogramm-onkologie.de/fileadmin/user_upload/Downloads/Leitlinien/Mammakarzinom_4_0/Version_4.4/LL_Mammakarzinom_Langversion_4.4.pdf, the recommended therapy for a hypothetical 79-year-old postmenopausal patient with right-sided invasive breast cancer (NST).
Use the current tumor details: pT3 (6.0 cm), pN2 (8/12), G3, L1, V0, Pn1, R1 (near the areola), ER 100%, PR 80%, HER2-negative, Ki67 approximately 20%
Breast-conserving therapy on the right side and right axillary dissection (Level 1 and 2) have already been performed.
Known pre-existing conditions include arterial hypertension, diabetes mellitus, and a past myocardial infarction.
List the hypothetical treatment options in a table. Use rows for Surgical Therapy, Chemotherapy, Radiotherapy, Anti-HER2 Therapy, and Endocrine Therapy. Use columns for Recommendation (yes/no), hypothetical medication (substance and dosage), Alternatives, and Risks. Use bullet points in the table.
Keep it as concise as possible, avoid repeating the case or the question. Be precise. Summarize the tabular recommendation in a written sentence below the table.

Case 24 – Recommendation ChatGPT

**Therapy options**	**Recommendation**	**Medication (Active agent and dosage)**	**Alternatives**	**Risks**
Surgery	Yes	Mastectomy of the right side	-	Infection, bleeding, scarring

Chemotherapy	Yes	Anthracycline-based chemotherapy (e.g. Epirubicin and Cyclophosphamide) followed by taxanes (e.g. Paclitaxel)	Alternatives: Chemotherapy without antracycline	Hematologic toxicity, nausea, hair loss, fatigue
Radiation	Yes	Post-surgery radiation of the chest wall and lymph pathways	-	Skin reaction, fatigue, rare long-term complications
Anti-HER2-Therapy	No	No anti-HER2 therapy due to HER2 negative status	-	-
Endocrine therapy	Yes	Aromatase inhibitor (e.g. Anastrozole) for 5-10 years	Alternatives: Tamoxifen, GnRH-Agonists	Joint pain, osteoporosis, hot flushes, menstrual bleeding disorder

The recommended treatment option for the 79-year-old postmenopausal patient with right-sided invasive breast cancer (NST) involves a mastectomy due to the tumor's size and lymphogenic metastatic status. An anthracycline-based chemotherapy, followed by taxanes, is advised to reduce the risk of recurrence. Postoperative radiation therapy to the chest wall and lymphatic pathways is recommended. As the tumor is HER2-negative, no anti-HER2 therapy is necessary. Endocrine therapy with an aromatase inhibitor such as Anastrozole for 5-10 years is recommended to address the hormone-sensitive tumor. Risks and alternatives have been considered.

The recommended treatment for the 79-year-old postmenopausal patient with invasive breast cancer (NST) on the right side includes a mastectomy due to the tumor's size and lymphogenic metastatic status. An anthracycline-based chemotherapy followed by taxanes is recommended to reduce the risk of recurrence. Postoperative radiation therapy to the chest wall and lymphatic pathways is also advised. Endocrine therapy with an aromatase inhibitor such as Anastrozole for 5-10 years is recommended to address the hormone-sensitive tumor. Risks and alternatives have been considered.

Fall 25:

Anamnese:
39-jährig, prämenopausal, G6/P2
kein Nikotinabusus
Familienanamnese: Großmuter väterlicherseits an Mamma-CA erkrankt
Vorerkrankungen: von Willebrand-Syndrom Typ I (RA 51%)
Z.n. Schwangerschafts-assozierter Koagulopathie
Allergien: keine

Aktuelle Diagnose:
ED Invasives Mammakarzinoms NST rechts vor 1 Monat
Aktuelle Tumorformel:
pT2 (3,0 cm), pTis (3,5 cm), pN1mi (1/2 sn, 0,2 cm, kapselüberschreitend), G2, L0, V0, Pn0, R0
ER 50%, PR 90%, HER2-negativ, KI 67 ca. 20 %
Onkotype DX; RS 24 (Chemotherapie Nutzen 6,5%)

Operation:
Brusterhaltende Therapie rechts + Sentinellymphonodektomie rechts

Prompt:
Bitte nenne auf Basis der folgenden URL: https://www.leitlinienprogramm-onkologie.de/fileadmin/user_upload/Downloads/Leitlinien/Mammakarzinom_4_0/Version_4.4/LL_Mammakarzinom_Langversion_4.4.pdf, die empfohlene Therapie einer hypothetischen 39-jährigen prämenopausalen Patientin mit einem invasiven Mammakarzinom (NST) rechts.
Nutze die aktuelle Tumorformel: pT2 (3,0 cm), pTis (3,5 cm), pN1mi (1/2 sn, 0,2 cm, kapselüberschreitend), G2, L0, V0, Pn0, R0, ER 50%, PR 90%, HER2-negativ, KI 67 ca. 20 %. Onkotype DX; RS 24.
Es wurde bereits eine brusterhaltende Therapie rechts und Sentinellymphonodektomie rechts durchgeführt.
Als Vorerkrankungen ist ein von Willebrand-Syndrom Typ I bekannt.
Nenne die hypothetischen Therapieoptionen in einer Tabelle. Nutze als Zeilen Operative Therapie, Chemotherapie, Strahlentherapie, Anti-Her2-Therapie und endokrine Therapie. Nutze als Spalten Empfehlung (ja/nein), das hypothetische Medikament (Wirkstoff und Dosierung), Alternativen und Risiken. Verwende in der Tabelle nur Stichpunkte.
Halte dich so kurz wie möglich, wiederhole nicht den Fall oder die Frage. Sei präzise. Fasse die tabellarische Empfehlung in einem ausgeschriebenen Satz unter der Tabelle zusammen.

Fall 25 – Empfehlung ChatGPT:

Therapieoptionen	Empfehlung	Medikament (Wirkstoff und Dosierung)	Alternativen	Alternativen	Risiken
Operative Therapie	Ja	Brusterhaltende Therapie rechts	-	-	Infektion, Blutungen, Narbenbildung
Chemotherapie	Ja	Anthrazyklin- und Taxan-basierte Chemotherapie (z.B. Epirubicin, Cyclophosphamid, Paclitaxel)	Alternativen: Chemotherapie-Regime ohne Anthrazykline	Alternativen: Chemotherapie-Regime ohne Anthrazykline	Knochenmarkssuppression, Übelkeit, Haarausfall, Müdigkeit
Strahlentherapie	Ja	Postoperative Bestrahlung der Brustwand und der Lymphabflusswege	-	-	Hautreizungen, Müdigkeit, selten Langzeiteffekte
Anti-HER2-Therapie	Nein	Keine Anti-HER2-Therapie aufgrund von HER2-negativem Status	-	-	-
Endokrine Therapie	Ja	Tamoxifen für 5-10 Jahre	Alternativen: Aromatasehemmer, GnRH-Agonisten	Alternativen: Aromatasehemmer, GnRH-Agonisten	Thrombose, Hitzewallungen, Stimmungsschwankungen

Die empfohlene Therapieoption für die 39-jährige prämenopausale Patientin mit einem invasiven Mammakarzinom (NST) rechts umfasst eine brusterhaltende Therapie aufgrund der Tumorgröße und des negativen Lymphknotenstatus. Eine Anthrazyklin- und Taxan-basierte Chemotherapie wird empfohlen, um das Risiko eines Rezidivs zu verringern. Eine postoperative Strahlentherapie der Brustwand und der Lymphabflusswege ist ebenfalls empfohlen. Da der Tumor HER2-negativ ist, ist keine Anti-HER2-Therapie erforderlich. Eine endokrine Therapie mit Tamoxifen für 5-10 Jahre wird empfohlen, um den hormonempfindlichen Tumor zu behandeln. Risiken und Alternativen wurden berücksichtigt.

Die empfohlene Therapie für die 39-jährige prämenopausale Patientin mit einem invasiven Mammakarzinom (NST) rechts umfasst eine brusterhaltende Therapie, gefolgt von einer Anthrazyklin- und Taxan-basierten Chemotherapie, um das Risiko eines Rezidivs zu verringern. Eine postoperative Strahlentherapie der Brustwand und der Lymphabflusswege wird ebenfalls empfohlen. Da der Tumor HER2-negativ ist, ist keine Anti-HER2-Therapie erforderlich. Eine endokrine Therapie mit Tamoxifen für 5-10 Jahre wird empfohlen, um den hormonempfindlichen Tumor zu behandeln. Risiken und Alternativen wurden berücksichtigt.

Translated version:

Case 25

Medical History:
39 years old, premenopausal, G6/P2
No nicotine use
Family history: Grandmother on the paternal side diagnosed with breast cancer
Medical conditions: von Willebrand syndrome type I (RA 51%)
History of pregnancy-associated coagulopathy
Allergies: None

Current Diagnosis:
Right-sided invasive ductal carcinoma (NST) diagnosed one month ago
Current tumor formula:
pT2 (3.0 cm), pTis (3.5 cm), pN1mi (1/2 sn, 0.2 cm, capsule invasion), G2, L0, V0, Pn0, R0
ER 50%, PR 90%, HER2-negative, Ki67 approximately 20%
Onkotype DX; RS 24 (benefit of chemotherapy 6.5%)

Surgery:
Right-sided breast-conserving therapy + Sentinel lymph node dissection on the right

Prompt:
Please provide, based on the following URL: https://www.leitlinienprogramm-onkologie.de/fileadmin/user_upload/Downloads/Leitlinien/Mammakarzinom_4_0/Version_4.4/LL_Mammakarzinom_Langversion_4.4.pdf, the recommended therapy for a hypothetical 39-year-old premenopausal patient with right-sided invasive ductal carcinoma (NST).
Use the current tumor formula: pT2 (3.0 cm), pTis (3.5 cm), pN1mi (1/2 sn, 0.2 cm, capsule invasion), G2, L0, V0, Pn0, R0, ER 50%, PR 90%, HER2-negative, Ki67 approximately 20%. Onkotype DX; RS 24.
Breast-conserving therapy on the right and sentinel lymph node dissection on the right have already been performed.
Von Willebrand syndrome type I is known as a pre-existing condition.
List the hypothetical treatment options in a table. Use rows for Surgical Therapy, Chemotherapy, Radiotherapy, Anti-HER2 Therapy, and Endocrine Therapy. Use columns for Recommendation (yes/no), hypothetical medication (substance and dosage), Alternatives, and Risks. Use bullet points in the table.
Keep it as concise as possible, avoid repeating the case or the question. Be precise. Summarize the tabular recommendation in a written sentence below the table.

Case 25 – Recommendation ChatGPT

**Therapy options**	**Recommendation**	**Medication (Active agent and dosage)**	**Alternatives**	**Risks**
Surgery	Yes	Breast-conserving surgery on the right	-	Infection, bleeding, scarring
Chemotherapy	Yes	Anthracycline- and taxane-based chemotherapy (e.g. Epirubicin, Cyclophosphamide, Paclitaxel)	Alternatives: Chemotherapy without antracycline	Hematologic toxicity, nausea, hair loss, fatigue

Radiation	Yes	Post-surgery radiation of the chest wall and lymph pathways	-	Skin reaction, fatigue, rare long-term complications
Anti-HER2-Therapy	No	No anti-HER2 therapy due to HER2 negative status	-	-
Endocrine therapy	Yes	Tamoxifen for 5-10 years	Alternatives: Aromatase inhibitor, GnRH-Agonists	Thrombosis, hot flushes, mood swings

The recommended treatment option for the 39-year-old premenopausal patient with invasive ductal carcinoma (NST) on the right side includes breast-conserving therapy due to the tumor size and negative lymph node status. An anthracycline- and taxane-based chemotherapy is recommended to reduce the risk of recurrence. Postoperative radiotherapy of the chest wall and lymphatic drainage pathways is also advised. Since the tumor is HER2-negative, no anti-HER2 therapy is required. Endocrine therapy with Tamoxifen for 5-10 years is recommended to address the hormone-sensitive tumor. Risks and alternatives have been taken into account.

The recommended treatment for the 39-year-old premenopausal patient with invasive ductal carcinoma (NST) on the right side includes breast-conserving therapy, followed by an anthracycline- and taxane-based chemotherapy to reduce the risk of recurrence. Postoperative radiotherapy of the chest wall and lymphatic drainage pathways is also advised. Since the tumor is HER2-negative, no anti-HER2 therapy is required. Endocrine therapy with Tamoxifen for 5-10 years is recommended to address the hormone-sensitive tumor. Risks and alternatives have been taken into account.

Fall 26:

Anamnese:
77-jährig, postmenopausal, G3/P2
Vorerkrankungen: Diabetes mellitus
Medikamenteneinnahme: Metformin
Allergien: Amoxicillin
kein Nikotinabusus
Familienanamnese: leer

Aktuelle Diagnose:
Invasiv lobuläres Mammakarzinom rechts (ED vor 1 Monat)
Aktuelle Tumorformel:
pT2, pN1a (1/9, 0,6 cm), G2, L0, V0, Pn1, R1
ER 100%; PR 60%; HER2-negativ, Ki67 20%

Operation:
Mammasegmentresektion rechts und Sentinellymphonodektomie rechts

Prompt:
Bitte nenne auf Basis der folgenden URL: https://www.leitlinienprogramm-onkologie.de/fileadmin/user_upload/Downloads/Leitlinien/Mammakarzinom_4_0/Version_4.4/LL_Mammakarzinom_Langversion_4.4.pdf, die empfohlene Therapie einer hypothetischen 77-jährigen postmenopausalen Patientin mit einem invasiv lobulären Mammakarzinom rechts.
Nutze die aktuelle Tumorformel: pT2, pN1a (1/9, 0,6 cm), G2, L0, V0, Pn1, R1, ER 100%; PR 60%; HER2-negativ, Ki67 20%
Es wurde bereits eine Mammasegmentresektion und Sentinellymphonodektomie rechts durchgeführt.
Als Vorerkrankungen ist ein Diabetes mellitus bekannt.
Nenne die hypothetischen Therapieoptionen in einer Tabelle. Nutze als Zeilen Operative Therapie, Chemotherapie, Strahlentherapie, Anti-Her2-Therapie und endokrine Therapie. Nutze als Spalten Empfehlung (ja/nein), das hypothetische Medikament (Wirkstoff und Dosierung), Alternativen und Risiken. Verwende in der Tabelle nur Stichpunkte.
Halte dich so kurz wie möglich, wiederhole nicht den Fall oder die Frage. Sei präzise. Fasse die tabellarische Empfehlung in einem ausgeschriebenen Satz unter der Tabelle zusammen.

Fall 26 – Empfehlung ChatGPT:

Therapieoptionen	Empfehlung	Medikament (Wirkstoff und Dosierung)	Alternativen	Risiken
Operative Therapie	Ja	Mammasegmentresektion rechts	-	Infektion, Blutungen, Narbenbildung
Chemotherapie	Nein	Keine Chemotherapie aufgrund des Tumorbiologies (lobuläres Karzinom, ER-positiv, HER2-negativ)	-	-
Strahlentherapie	Ja	Postoperative Bestrahlung der Brustwand und der Lymphabflusswege	-	Hautreizungen, Müdigkeit, selten Langzeiteffekte
Anti-HER2-Therapie	Nein	Keine Anti-HER2-Therapie aufgrund von HER2-negativem Status	-	-
Endokrine Therapie	Ja	Aromatasehemmer (z.B. Anastrozol) für 5-10 Jahre	Alternativen: Tamoxifen	Gelenkschmerzen, Osteoporose, Hitzewallungen

Die empfohlene Therapieoption für die 77-jährige postmenopausale Patientin mit einem invasiven lobulären Mammakarzinom rechts umfasst eine Mammasegmentresektion rechts, um den Tumor zu entfernen. Aufgrund des Tumorbiologies (lobuläres Karzinom, ER-positiv, HER2-negativ) wird keine Chemotherapie empfohlen. Eine postoperative Strahlentherapie der Brustwand und der Lymphabflusswege wird empfohlen. Aufgrund des HER2-negativen Status ist keine Anti-HER2-Therapie erforderlich. Eine endokrine Therapie mit einem Aromatasehemmer wie Anastrozol für 5-10 Jahre wird empfohlen, um den hormonempfindlichen Tumor zu behandeln. Risiken und Alternativen wurden berücksichtigt.

Die empfohlene Therapie für die 77-jährige postmenopausale Patientin mit einem invasiven lobulären Mammakarzinom rechts umfasst eine Mammasegmentresektion rechts, gefolgt von einer postoperativen Strahlentherapie der Brustwand und der Lymphabflusswege. Aufgrund des Tumorbiologies (lobuläres Karzinom, ER-positiv, HER2-negativ) wird keine Chemotherapie empfohlen. Da der Tumor HER2-negativ ist, ist auch keine Anti-HER2-Therapie erforderlich. Eine endokrine Therapie mit einem Aromatasehemmer wie Anastrozol für 5-10 Jahre wird empfohlen, um den hormonempfindlichen Tumor zu behandeln. Risiken und Alternativen wurden berücksichtigt.

Translated version:

Case 26

Medical History:
77 years old, postmenopausal, G3/P2
Medical history: Diabetes mellitus
Medication: Metformin
Allergies: Amoxicillin
No nicotine use
Family history: None

Current Diagnosis:
Invasive lobular carcinoma on the right side (diagnosed one month ago)
Current tumor formula: pT2, pN1a (1/9, 0.6 cm), G2, L0, V0, Pn1, R1
ER 100%, PR 60%, HER2-negative, Ki67 20%

Procedure:
Right-sided breast segment resection and right sentinel lymph node dissection

Prompt:
Please, based on the following URL: https://www.leitlinienprogramm-onkologie.de/fileadmin/user_upload/Downloads/Leitlinien/Mammakarzinom_4_0/Version_4.4/LL_Mammakarzinom_Langversion_4.4.pdf, provide the recommended therapy for a hypothetical 77-year-old postmenopausal patient with invasive lobular carcinoma on the right side. Utilize the current tumor formula: pT2, pN1a (1/9, 0.6 cm), G2, L0, V0, Pn1, R1, ER 100%; PR 60%; HER2-negative, Ki67 20%. A right-sided breast segment resection and sentinel lymph node dissection have already been performed. Diabetes mellitus is known as a pre-existing condition.
List the hypothetical treatment options in a table. Use rows for Surgical Therapy, Chemotherapy, Radiotherapy, Anti-HER2 Therapy, and Endocrine Therapy. Use columns for Recommendation (yes/no), hypothetical medication (substance and dosage), Alternatives, and Risks. Use bullet points in the table.
Keep it as concise as possible, avoid repeating the case or the question. Be precise. Summarize the tabular recommendation in a written sentence below the table.

Case 26 – Recommendation ChatGPT

Therapy options	Recommendation	Medication (Active agent and dosage)	Alternatives	Risks
Surgery	Yes	Breast segment resection of the right side	-	Infection, bleeding, scarring
Chemotherapy	No	No chemotherapy due to tumor biology (Lobular carcinoma, ER-positive, HER2-negative)	-	-
Radiation	Yes	Post-surgery radiation of the chest wall and lymph pathways	-	Skin reaction, fatigue, rare long-term complications

Anti-HER2-Therapy	No	No anti-HER2 therapy due to HER2 negative status	-	-
Endocrine therapy	Yes	Aromatase inhibitor (e.g. anastrozole) for 5-10 years	Alternatives: Tamoxifen	Joint pain, osteoporosis, hot flushes

The recommended treatment option for the 77-year-old postmenopausal patient with invasive lobular carcinoma in the right breast includes a right breast segmental resection to remove the tumor. Due to the tumor biology (lobular carcinoma, ER-positive, HER2-negative), chemotherapy is not recommended. Postoperative radiation therapy of the chest wall and lymphatic pathways is advised. Anti-HER2 therapy is not required due to the HER2-negative status. Endocrine therapy with an aromatase inhibitor like Anastrozole for 5-10 years is recommended to address the hormone-sensitive tumor. Risks and alternatives have been taken into consideration.

The recommended treatment for the 77-year-old postmenopausal patient with invasive lobular carcinoma in the right breast involves a right breast segmental resection, followed by postoperative radiation therapy of the chest wall and lymphatic pathways. Due to the tumor biology (lobular carcinoma, ER-positive, HER2-negative), chemotherapy is not recommended. Since the tumor is HER2-negative, anti-HER2 therapy is also not required. Endocrine therapy with an aromatase inhibitor like Anastrozole for 5-10 years is recommended to address the hormone-sensitive tumor. Risks and alternatives have been taken into consideration.

Fall 27:

Anamnese:
69-jährig, postmenopausal, G2/P2
Z.n. Rippenserienfraktur rechts vor 1J
Z.n. Zahnwurzelextraktion vor 1 Monat
Familienanamnese: Magen-Karzinom
Arterielle Hypertonie, Diabetes mellitus, Z.n. Thrombose
Von-Willebrand-Syndrom Typ 1

Aktuelle Diagnose:
2. Mammakarzinomrezidiv im Bereich der Ablationarbe links
rcT1b (10mm), cN0, Mx (V.a. ossäre Metastase des Corpus sterni in Skelett-Szintigrafie)
ER 100%; PR 35%; HER2-negativ; Ki67 ca. 15%, G2
Nebenbefundlich Fettgewebsnekrose der Mamma rechts sowie mehrere Zysten

Epikrise:
Vor 9J ED DCIS links; Brusterhaltende Therapie, anschließend Radiatio
Vor 6J Mammakarzinom links
-pT1b (8 mm) pN0 (0/14, 6 sn); G2, R0, pTis (5 cm); ER 100%, PR 100%, HER2-negativ, Ki67 15 %
-Operation (vor 6J): Ablatio mamma links mit Sentinel-Lymphonodektomie der Axilla links
Therapie mit Tamoxifen für 1J, anschließend für 3J Letrozol
-Unter Tamoxifen Nekrose des Sprunggelenkes links
-unter Letrozol Osteoporose mit Brüchen im Bereich des rechten Fußes

1. Mammakarzinomrezidiv im Bereich der Ablationarbe (vor 1J)
-Staging unauffällig
-Operation: Lokalexzision des Rezidivs an der linken Thoraxwand
-rpT1a (m, 0,4 cm und 0,3 cm Durchmesser), rpNx, G2, L0, V0, Pn0, R0,
-ER 100%, PR100%, HER2-negativ, Ki67 ca. 15%
-Tumorkonferenz-Empfehlung zur endokrinen Therapie, von Patientin abgelehnt bei Osteoporose

Prompt:
Bitte nenne auf Basis der folgenden URL: https://www.leitlinienprogramm-onkologie.de/fileadmin/user_upload/Downloads/Leitlinien/Mammakarzinom_4_0/Version_4.4/LL_Mammakarzinom_Langversion_4.4.pdf, die empfohlene Therapie einer hypothetischen 69-jährigen postmenopausalen Patientin mit dem zweiten Rezidiv eines Mammakarzinoms der Ablationarbe links.
Nutze die aktuelle Tumorformel: rcT1b (10mm), cN0, Mx (Verdacht auf ossäre Metastase des Corpus sterni in Skelett-Szintigrafie), ER 100%; PR 35%; HER2-negativ; Ki67 ca. 15%, G2
Berücksichtige die Vorgeschichte der hypothetischen Patientin mit einem Mammakarzinom vor 6 Jahren links, dass mittels Ablatio und Sentinellymphonodektomie links, sowie endokriner Therapie ausbehandelt wurde. Im Anschluss kam es bereits zu einem ersten Rezidiv, dass mittels Exzision entfernt wurde.
Als Vorerkrankungen sind eine arterielle Hypertonie, ein Diabetes mellitus und mehrere Frakturen bekannt.
Nenne die hypothetischen Therapieoptionen in einer Tabelle. Nutze als Zeilen Operative Therapie, Chemotherapie, Strahlentherapie, Anti-Her2-Therapie und endokrine Therapie. Nutze als Spalten Empfehlung (ja/nein), das hypothetische Medikament (Wirkstoff und Dosierung), Alternativen und Risiken. Verwende in der Tabelle nur Stichpunkte.
Halte dich so kurz wie möglich, wiederhole nicht den Fall oder die Frage. Sei präzise. Fasse die tabellarische Empfehlung in einem ausgeschriebenen Satz unter der Tabelle zusammen.

Fall 27 – Empfehlung ChatGPT:

Therapieoptionen	Empfehlung	Medikament (Wirkstoff und Dosierung)	Alternativen	Risiken
Operative Therapie	Ja	Resektion des Rezidivs	-	Infektion, Blutungen, Narbenbildung
Chemotherapie	Ja	Taxan-basierte Chemotherapie	Anthrazyklin-basierte Chemotherapie	Knochenmarksuppress Übelkeit, Haarausfall
Strahlentherapie	Ja	Lokale Strahlentherapie des Rezidivs	-	Hautreizungen, Müdigk selten Langzeiteffekte
Anti-HER2-Therapie	Nein	Keine Anti-HER2-Therapie aufgrund von HER2-negativem Status	-	-
Endokrine Therapie	Ja	Aromatasehemmer (z.B. Anastrozol) für 5-10 Jahre	Tamoxifen, GNRH-Analoga	Gelenkschmerzen, Osteoporose, Hitzewallungen

Die empfohlene Therapieoption für die 69-jährige postmenopausale Patientin mit dem zweiten Rezidiv eines Mammakarzinoms der Ablationarbe links umfasst die Resektion des Rezidivs. Aufgrund der Tumorbiologie (ER-positiv, HER2-negativ) wird eine Chemotherapie mit einem Taxan-basierten Regime empfohlen, wobei eine Anthrazyklin-basierte Chemotherapie eine alternative Option sein kann. Eine lokale Strahlentherapie des Rezidivs wird ebenfalls empfohlen. Da der Tumor HER2-negativ ist, ist keine Anti-HER2-Therapie erforderlich. Eine endokrine Therapie mit einem Aromatasehemmer wie Anastrozol für 5-10 Jahre wird empfohlen, um den hormonempfindlichen Tumor zu behandeln. Risiken und Alternativen wurden berücksichtigt.

Die empfohlene Therapie für die 69-jährige postmenopausale Patientin mit dem zweiten Rezidiv eines Mammakarzinoms der Ablationarbe links umfasst die Resektion des Rezidivs, gefolgt von einer Chemotherapie mit einem Taxan-basierten Regime, um mögliche Metastasen zu behandeln. Eine lokale Strahlentherapie des Rezidivs wird ebenfalls empfohlen. Eine endokrine Therapie mit einem Aromatasehemmer wie Anastrozol für 5-10 Jahre wird empfohlen, um den hormonempfindlichen Tumor zu behandeln. Risiken und Alternativen wurden berücksichtigt.

Translated version:

Case 27

Medical History:
69 years old, postmenopausal, G2/P2
History of rib series fracture on the right side 1 year ago
History of dental root extraction 1 month ago
Family history: Gastric carcinoma
Arterial hypertension, diabetes mellitus, history of thrombosis
Von Willebrand Syndrome Type 1

Current Diagnosis:
2nd recurrence of breast carcinoma in the area of the left ablation scar
rcT1b (10mm), cN0, Mx (Suspicion of osseous metastasis of the Corpus sterni in Skeletal Scintigraphy)
ER 100%; PR 35%; HER2-negative; Ki67 approximately 15%, G2
Incidentally, fat necrosis of the right breast and several cysts

Clinical Course:
9 years ago: Ductal Carcinoma In Situ (DCIS) on the left; Breast-conserving therapy, followed by radiation
6 years ago: Breast carcinoma on the left
-pT1b (8 mm) pN0 (0/14, 6 sn); G2, R0, pTis (5 cm); ER 100%, PR 100%, HER2-negative, Ki67 15%
-Surgery (6 years ago): Left mastectomy with sentinel lymph node dissection of the left axilla
Therapy with Tamoxifen for 1 year, followed by Letrozole for 3 years
-Under Tamoxifen, necrosis of the left ankle
-Under Letrozole, osteoporosis with fractures in the area of the right foot

1st Recurrence of breast carcinoma in the area of the ablation scar (1 year ago)
-Staging inconspicuous
-Surgery: Local excision of the recurrence on the left thoracic wall
-rpT1a (m, 0.4 cm and 0.3 cm diameter), rpNx, G2, L0, V0, Pn0, R0,
-ER 100%, PR100%, HER2-negative, Ki67 approximately 15%
-Tumor board recommendation for endocrine therapy, declined by the patient due to osteoporosis

Prompt:
Please state, based on the following URL: https://www.leitlinienprogramm-onkologie.de/fileadmin/user_upload/Downloads/Leitlinien/Mammakarzinom_4_0/Version_4.4/LL_Mammakarzinom_Langversion_4.4.pdf, the recommended therapy for a hypothetical 69-year-old postmenopausal patient with the second recurrence of breast cancer in the area of the left ablation scar.
Use the current tumor formula: rcT1b (10mm), cN0, Mx (suspicion of osseous metastasis of the Corpus sterni in skeletal scintigraphy), ER 100%; PR 35%; HER2-negative; Ki67 approximately 15%, G2.
Consider the medical history of the hypothetical patient with breast cancer six years ago, treated with left mastectomy, sentinel lymph node dissection, and subsequent endocrine therapy. There has already been a first recurrence that was removed by excision.
Known pre-existing conditions include arterial hypertension, diabetes mellitus, and several fractures.
List the hypothetical treatment options in a table. Use rows for Surgical Therapy, Chemotherapy, Radiotherapy, Anti-HER2 Therapy, and Endocrine Therapy. Use columns for Recommendation (yes/no), hypothetical medication (substance and dosage), Alternatives, and Risks. Use bullet points in the table.
Keep it as concise as possible, avoid repeating the case or the question. Be precise. Summarize the tabular recommendation in a written sentence below the table.

Case 27 – Recommendation ChatGPT

**Therapy options**	**Recommendation**	**Medication (Active agent and dosage)**	**Alternatives**	**Risks**
Surgery	Yes	Resection of the relapse cancer	-	Infection, bleeding, scarring
Chemotherapy	Yes	Taxane-based chemotherapy	Anthracycline-based chemotherapy	Hematologic toxicity, nausea, hair loss
Radiation	Yes	Local radiation of the relapse	-	Skin reaction, fatigue, rare long-term complications
Anti-HER2-Therapy	No	No anti-HER2 therapy due to HER2 negative status	-	-
Endocrine therapy	Yes	Aromatase inhibitor (e.g. Anastrozole) for 5-10 years	Tamoxifen, GnRH-Agonists	Joint pain, osteoporosis, hot flushes

The recommended treatment option for the 69-year-old postmenopausal patient with the second recurrence of breast carcinoma in the left ablation area involves the resection of the recurrence. Due to the tumor biology (ER-positive, HER2-negative), chemotherapy with a taxane-based regimen is recommended, with an anthracycline-based chemotherapy being an alternative option. Local radiotherapy for the recurrence is also advised. As the tumor is HER2-negative, no anti-HER2 therapy is required. Endocrine therapy with an aromatase inhibitor such as Anastrozole for 5-10 years is recommended to address the hormone-sensitive tumor. Risks and alternatives have been taken into account.

The recommended treatment for the 69-year-old postmenopausal patient with the second recurrence of breast carcinoma in the left ablation area includes the resection of the recurrence, followed by chemotherapy with a taxane-based regimen to address potential metastases. Local radiotherapy for the recurrence is also advised. Endocrine therapy with an aromatase inhibitor such as Anastrozole for 5-10 years is recommended to treat the hormone-sensitive tumor. Risks and alternatives have been taken into account.

Fall 28:

Anamnese:
72-jährig, postmenopausal, G3/P3
Keine Hormoneinnahme
Vorerkrankungen:
-Art. Hypertonie
-Hypothyreose
-Z.n. Appendektomie
Keine Allergien
Medikation: L-Thyroxin, Antihypertensivum
Familienanamnese leer bzgl. Malignomen

Aktuelle Diagnose:
Vor 1 Monat ED Invasives Mammakarzinom rechts
Initial: cT2 (sonographisch 22mm, mammographisch 35mm Mikrokalk), cN0 (histologisch unauffälliger Lymphknoten)
Aktuelle Tumorformel:
pT2 (2,1 cm), pTis (1,5 cm), pN0 (0/3 sn), G2, L0, V0, Pn0, R0 (mit Nachresektaten)
ER 100 %, PR 95 %, HER2-negativ, Ki67 ca. 10-15 %

Operation:
Brusterhaltende Therapie und Sentinellymphonodektomie rechts

Prompt:
Bitte nenne auf Basis der folgenden URL: https://www.leitlinienprogramm-onkologie.de/fileadmin/user_upload/Downloads/Leitlinien/Mammakarzinom_4_0/Version_4.4/LL_Mammakarzinom_Langversion_4.4.pdf, die empfohlene Therapie einer hypothetischen 72-jährigen Patientin mit einem invasiven Mammakarzinom rechts.
Nutze die aktuelle Tumorformel: pT2 (2,1 cm), pTis (1,5 cm), pN0 (0/3 sn), G2, L0, V0, Pn0, R0, ER 100 %, PR 95 %, HER2-negativ, Ki67 ca. 10-15 %
Es wurde bereits eine brusterhaltende Therapie und Sentinellymphonodektomie rechts durchgeführt.
Als Vorerkrankungen sind eine arterielle Hypertonie und eine Hypothyreose bekannt.
Nenne die hypothetischen Therapieoptionen in einer Tabelle. Nutze als Zeilen Operative Therapie, Chemotherapie, Strahlentherapie, Anti-Her2-Therapie und endokrine Therapie. Nutze als Spalten Empfehlung (ja/nein), das hypothetische Medikament (Wirkstoff und Dosierung), Alternativen und Risiken. Verwende in der Tabelle nur Stichpunkte.
Halte dich so kurz wie möglich, wiederhole nicht den Fall oder die Frage. Sei präzise. Fasse die tabellarische Empfehlung in einem ausgeschriebenen Satz unter der Tabelle zusammen.

Fall 28 – Empfehlung ChatGPT:

Therapieoptionen	Empfehlung	Medikament (Wirkstoff und Dosierung)	Alternativen	Risiken
Operative Therapie	Ja	Resektion des Tumors	-	Infektion, Blutungen, Narbenbildung
Chemotherapie	Nein	Keine Chemotherapie aufgrund der Tumorbiologie (ER- und PR-positiv, HER2-negativ)	-	-
Strahlentherapie	Ja	Lokale Strahlentherapie nach brusterhaltender Therapie	-	Hautreizungen, Müdigkeit, selten Langzeiteffekte
Anti-HER2-Therapie	Nein	Keine Anti-HER2-Therapie aufgrund von HER2-negativem Status	-	-
Endokrine Therapie	Ja	Aromatasehemmer (z.B. Anastrozol) für 5-10 Jahre	Tamoxifen, GNRH-Analoga	Gelenkschmerzen, Osteoporose, Hitzewallungen

Die empfohlene Therapieoption für die 72-jährige Patientin mit einem invasiven Mammakarzinom rechts umfasst die Resektion des Tumors. Aufgrund der Tumorbiologie (ER- und PR-positiv, HER2-negativ) wird keine Chemotherapie empfohlen. Eine lokale Strahlentherapie nach brusterhaltender Therapie wird empfohlen, um das Risiko eines lokalen Rezidivs zu reduzieren. Aufgrund des HER2-negativen Status ist keine Anti-HER2-Therapie erforderlich. Eine endokrine Therapie mit einem Aromatasehemmer wie Anastrozol für 5-10 Jahre wird empfohlen, um den hormonempfindlichen Tumor zu behandeln. Risiken und Alternativen wurden berücksichtigt.

Die empfohlene Therapie für die 72-jährige Patientin mit einem invasiven Mammakarzinom rechts umfasst die Resektion des Tumors, gefolgt von einer lokalen Strahlentherapie nach brusterhaltender Therapie. Eine endokrine Therapie mit einem Aromatasehemmer wie Anastrozol wird empfohlen, um den hormonempfindlichen Tumor zu behandeln. Risiken und Alternativen wurden berücksichtigt. Chemotherapie und Anti-HER2-Therapie sind aufgrund der Tumorbiologie (ER- und PR-positiv, HER2-negativ) nicht erforderlich.

Translated version:

Case 28

Medical History:
72 years old, postmenopausal, G3/P3
No hormone intake
Pre-existing conditions:
- Arterial hypertension
- Hypothyroidism
- History of appendectomy
No allergies
Medication: L-Thyroxine, Antihypertensive
Family history: No known malignancies

Current Diagnosis:
1 month ago, diagnosed with invasive breast carcinoma on the right side
Initial assessment: cT2 (sonographically 22mm, mammographically 35mm microcalcifications), cN0 (histologically unremarkable lymph nodes)
Current Tumor Formula:
pT2 (2.1 cm), pTis (1.5 cm), pN0 (0/3 sentinel nodes), G2, L0, V0, Pn0, R0 (with resection margins)
ER 100%, PR 95%, HER2-negative, Ki67 approximately 10-15%

Surgery:
Underwent breast-conserving therapy and sentinel lymph node dissection on the right side.

Prompt:
Please provide, based on the following URL: https://www.leitlinienprogramm-onkologie.de/fileadmin/user_upload/Downloads/Leitlinien/Mammakarzinom_4_0/Version_4.4/LL_Mammakarzinom_Langversion_4.4.pdf, the recommended therapy for a hypothetical 72-year-old patient with invasive breast carcinoma on the right side.
Utilize the current tumor formula: pT2 (2.1 cm), pTis (1.5 cm), pN0 (0/3 sn), G2, L0, V0, Pn0, R0, ER 100%, PR 95%, HER2-negative, Ki67 approximately 10-15%.
A breast-conserving therapy and sentinel lymph node dissection on the right side have already been performed.
Known pre-existing conditions include arterial hypertension and hypothyroidism.
List the hypothetical treatment options in a table. Use rows for Surgical Therapy, Chemotherapy, Radiotherapy, Anti-HER2 Therapy, and Endocrine Therapy. Use columns for Recommendation (yes/no), hypothetical medication (substance and dosage), Alternatives, and Risks. Use bullet points in the table.
Keep it as concise as possible, avoid repeating the case or the question. Be precise. Summarize the tabular recommendation in a written sentence below the table.

Case 28 – Recommendation ChatGPT

Therapy options	Recommendation	Medication (Active agent and dosage)	Alternatives	Risks
Surgery	Yes	Resection of the tumor	-	Infection, bleeding, scarring
Chemotherapy	No	No chemotherapy due to tumor biology (ER- and	-	-

		PR-positive, HER2-negative)		
Radiation	Yes	Local radiation therapy after breast-conserving therapy	-	Skin reaction, fatigue, rare long-term complications
Anti-HER2-Therapy	No	No anti-HER2 therapy due to HER2 negative status	-	-
Endocrine therapy	Yes	Aromatase inhibitor (e.g. Anastrozole) for 5-10 years	Tamoxifen, GnRH-Agonists	Joint pain, osteoporosis, hot flushes

The recommended treatment option for the 72-year-old patient with invasive breast carcinoma on the right side involves the resection of the tumor. Due to the tumor biology (ER- and PR-positive, HER2-negative), chemotherapy is not recommended. Local radiotherapy following breast-conserving therapy is advised to reduce the risk of local recurrence. Since the tumor is HER2-negative, no anti-HER2 therapy is required. Endocrine therapy with an aromatase inhibitor such as Anastrozole for 5-10 years is recommended to treat the hormone-sensitive tumor. Risks and alternatives have been taken into account.

The recommended treatment for the 72-year-old patient with invasive breast carcinoma on the right side includes the resection of the tumor, followed by local radiotherapy after breast-conserving therapy. Endocrine therapy with an aromatase inhibitor such as Anastrozole is recommended to treat the hormone-sensitive tumor. Risks and alternatives have been taken into account. Chemotherapy and anti-HER2 therapy are not necessary due to the tumor biology (ER- and PR-positive, HER2-negative).

Fall 29:

Anamnese:
60-jährig, G2/P1, postmenopausal
keine Vorerkrankungen
keine Voroperationen
Allergie: Nickel
Medikamente: keine
Positive FA: Schwester mit 39 J. an Mammacarcinom erkrankt, mit 45 an Rezidiv

Aktuelle Diagnose:
Vor 1 Monat ED Invasiv lobuläres Mammacarcinom rechts
Initial cT1b, cN1, cMx, G2; ER 100%, PR <10 %, HER2-negativ, Ki67 25%
Zudem DCIS rechts
Aktuelle Tumorformel:
pT1c (2,0 cm Durchmesser), pTis (DCIS, 1,5 cm Durchmesser), pN2a (4/16), G2, L0, V0, Pn0, R1 (ventral, invasives Karzinom), Rx (lateral, DCIS)
Immunphänotyp: ER 90%; PR 15%; HER2-negativ; Ki67 ca. 15%.

Neu: Suspekter Herd mammogarpahisch Mamma rechts (außerhalb der Segmentresektion)

Operation:
Mammasegmentresektion rechts nach Drahtmarkierung, Axilladissektion rechts

Prompt:
Bitte nenne auf Basis der folgenden URL: https://www.leitlinienprogramm-onkologie.de/fileadmin/user_upload/Downloads/Leitlinien/Mammakarzinom_4_0/Version_4.4/LL_Mammakarzinom_Langversion_4.4.pdf, die empfohlene Therapie einer hypothetischen 60-jährigen postmenopausale Patientin mit einem invasiv lobulären Mammakarzinom rechts, sowie einem DCIS rechts.
Nutze die aktuelle Tumorformel: pT1c (2,0 cm Durchmesser), pTis (DCIS, 1,5 cm Durchmesser), pN2a (4/16), G2, L0, V0, Pn0, R1 (ventral, invasives Karzinom), Rx (lateral, DCIS), ER 90%; PR 15%; HER2-negativ; Ki67 ca. 15%.
Es wurde bereits eine Mammasegmentresektion rechts und eine Axilladissektion rechts durchgeführt.
Berücksichtige die Vorgeschichte der hypothetischen Patientin mit einer positiven Familienanamnese für Mammakarzinome.
Es sind keine Vorerkrankungen bekannt.
Nenne die hypothetischen Therapieoptionen in einer Tabelle. Nutze als Zeilen Operative Therapie, Chemotherapie, Strahlentherapie, Anti-Her2-Therapie und endokrine Therapie. Nutze als Spalten Empfehlung (ja/nein), das hypothetische Medikament (Wirkstoff und Dosierung), Alternativen und Risiken. Verwende in der Tabelle nur Stichpunkte.
Halte dich so kurz wie möglich, wiederhole nicht den Fall oder die Frage. Sei präzise. Fasse die tabellarische Empfehlung in einem ausgeschriebenen Satz unter der Tabelle zusammen.

Fall 29 – Empfehlung ChatGPT:

Therapieoptionen	Empfehlung	Medikament (Wirkstoff und Dosierung)	Alternativen	Risiken
Operative Therapie	Ja	Mastektomie oder brusterhaltende Therapie mit adjuvanter Radiotherapie	-	Infektion, Blutungen, Narbenbildung
Chemotherapie	Ja	Gemcitabin plus Docetaxel	Anthrazykline, Taxane	Übelkeit, Haarausfall, Immunsuppression
Strahlentherapie	Ja	Adjuvante Radiotherapie nach brusterhaltender Therapie	-	Hautreizungen, Müdigkeit, selten Langzeiteffekte
Anti-HER2-Therapie	Nein	Keine Anti-HER2-Therapie aufgrund von HER2-negativem Status	-	-
Endokrine Therapie	Ja	Aromatasehemmer (z.B. Anastrozol) für 5-10 Jahre	Tamoxifen, GNRH-Analoga	Gelenkschmerzen, Osteoporose, Hitzewallungen

Die empfohlene Therapieoption für die 60-jährige postmenopausale Patientin mit einem invasiv lobulären Mammakarzinom rechts und einem DCIS rechts umfasst entweder eine Mastektomie oder eine brusterhaltende Therapie mit adjuvanter Radiotherapie. Eine Chemotherapie mit Gemcitabin plus Docetaxel wird empfohlen, um das Risiko eines Rezidivs zu reduzieren. Eine endokrine Therapie mit einem Aromatasehemmer wie Anastrozol für 5-10 Jahre wird empfohlen, um den hormonempfindlichen Tumor zu behandeln. Risiken und Alternativen wurden berücksichtigt. Eine Anti-HER2-Therapie ist aufgrund des HER2-negativen Status nicht erforderlich.

Die empfohlene Therapie für die 60-jährige postmenopausale Patientin mit einem invasiv lobulären Mammakarzinom rechts und einem DCIS rechts umfasst entweder eine Mastektomie oder eine brusterhaltende Therapie mit adjuvanter Radiotherapie. Eine Chemotherapie mit Gemcitabin plus Docetaxel wird empfohlen, um das Risiko eines Rezidivs zu reduzieren. Eine endokrine Therapie mit einem Aromatasehemmer wie Anastrozol für 5-10 Jahre wird empfohlen, um den hormonempfindlichen Tumor zu behandeln. Risiken und Alternativen wurden berücksichtigt. Eine Anti-HER2-Therapie ist aufgrund des HER2-negativen Status nicht erforderlich.

Translated version:

Case 29

Medical History:
60 years old, G2/P1, postmenopausal
No previous illnesses
No prior surgeries
Allergy: Nickel
Medications: None
Family History: Sister diagnosed with breast carcinoma at 39 years old, with recurrence at 45

Current Diagnosis:
1 month ago, invasive lobular carcinoma in the right breast
Initial staging: cT1b, cN1, cMx, G2; ER 100%, PR <10%, HER2-negative, Ki67 25%
Additionally, DCIS in the right breast
Current tumor formula:
pT1c (2.0 cm diameter), pTis (DCIS, 1.5 cm diameter), pN2a (4/16), G2, L0, V0, Pn0, R1 (ventral, invasive carcinoma), Rx (lateral, DCIS)
Immunophenotype: ER 90%; PR 15%; HER2-negative; Ki67 approximately 15%.

New: Suspicious lesion mammographically in the right breast (outside the segmental resection)

Surgery:
Right breast segmental resection after wire marking, right axillary dissection

Prompt:
Please provide, based on the following URL: https://www.leitlinienprogramm-onkologie.de/fileadmin/user_upload/Downloads/Leitlinien/Mammakarzinom_4_0/Version_4.4/LL_Mammakarzinom_Langversion_4.4.pdf, the recommended therapy for a hypothetical 60-year-old postmenopausal patient with invasive lobular carcinoma in the right breast and DCIS in the right breast.
Use the current tumor formula: pT1c (2.0 cm diameter), pTis (DCIS, 1.5 cm diameter), pN2a (4/16), G2, L0, V0, Pn0, R1 (ventral, invasive carcinoma), Rx (lateral, DCIS), ER 90%; PR 15%; HER2-negative; Ki67 approximately 15%.
A right breast segmental resection and right axillary dissection have already been performed.
Consider the patient's history with a positive family history of breast carcinomas.
No other pre-existing conditions are known.
List the hypothetical treatment options in a table. Use rows for Surgical Therapy, Chemotherapy, Radiotherapy, Anti-HER2 Therapy, and Endocrine Therapy. Use columns for Recommendation (yes/no), hypothetical medication (substance and dosage), Alternatives, and Risks. Use bullet points in the table.
Keep it as concise as possible, avoid repeating the case or the question. Be precise. Summarize the tabular recommendation in a written sentence below the table.

Case 29 – Recommendation ChatGPT

**Therapy options**	**Recommendation**	**Medication (Active agent and dosage)**	**Alternatives**	**Risks**
Surgery	Yes	Mastectomy or breast-conserving surgery with	-	Infection, bleeding, scarring

		adjuvant radiotherapy		
Chemotherapy	Yes	Gemcitabine and docetaxel	Anthracycline, taxanes	Nausea, hair loss, immunosuppression
Radiation	Yes	Adjuvant radiotherapy after breast-conserving surgery	-	Skin reaction, fatigue, rare long-term complications
Anti-HER2-Therapy	No	No anti-HER2 therapy due to HER2 negative status	-	-
Endocrine therapy	Yes	Aromatase inhibitor (e.g. Anastrozole) for 5-10 years	Tamoxifen, GnRH-Agonists	Joint pain, osteoporosis, hot flushes

The recommended treatment option for the 60-year-old postmenopausal patient with invasive lobular carcinoma in the right breast and DCIS in the right breast includes either a mastectomy or breast-conserving therapy with adjuvant radiotherapy. Chemotherapy with gemcitabine plus docetaxel is recommended to reduce the risk of recurrence. Endocrine therapy with an aromatase inhibitor such as anastrozole for 5-10 years is advised to treat the hormone-sensitive tumor. Risks and alternatives have been considered. Anti-HER2 therapy is not required due to the HER2-negative status.

The recommended treatment for the 60-year-old postmenopausal patient with invasive lobular carcinoma in the right breast and DCIS in the right breast includes either a mastectomy or breast-conserving therapy with adjuvant radiotherapy. Chemotherapy with gemcitabine plus docetaxel is advised to reduce the risk of recurrence. Endocrine therapy with an aromatase inhibitor such as anastrozole for 5-10 years is recommended to address the hormone-sensitive tumor. Risks and alternatives have been considered. Anti-HER2 therapy is not required due to the HER2-negative status.

Fall 30:

Anamnese:
60-jährig, postmenopausal
Vorerkrankungen:
Diabetes mellitus (Metformin)
Arterielle Hypertonie
Asthma bronchiale
Coxarthorse mit chron. Schmerzen

Aktuelle Diagnose:
Vor 1 Monat ED eines fortgeschrittenen Mammakarzinoms rechts mit Exulzeration in der Submammärfalte
Tumorstadium: cT4b, multizentrischer Befund, cN1, M1 (Lungenmetastase rechter Oberlappen)
Herd 1: 14 mm im axillären Ausläufer
Herd 2: oben außen von 36 x 28 mm
Herd 3: in der Submammerfalte 6 Uhr von 36 x 28 mm, N1 (mindestens 3 Lymphknoten, max. 23 mm)
ER 0%; PR 0%; HER2-negativ; Ki67 75 %, PD-L1 TPS 0% und IC 1

Prompt:
Bitte nenne auf Basis der folgenden URL: https://www.leitlinienprogramm-onkologie.de/fileadmin/user_upload/Downloads/Leitlinien/Mammakarzinom_4_0/Version_4.4/LL_Mammakarzinom_Langversion_4.4.pdf, die empfohlene Therapie einer hypothetischen 60-jährigen postmenopausalen Patientin mit einem fortgeschrittenen Mammakarzinom rechts mit Exulzeration in der Submammärfalte.
Nutze die aktuelle Tumorformel: cT4b, multizentrischer Befund, cN1, M1, ER 0%; PR 0%; HER2-negativ; Ki67 75 %, PD-L1 TPS 0% und IC 1
Es wurde bisher keine Therapie durchgeführt.
Als Vorerkrankungen sind ein Diabetes mellitus, Arterielle Hypertonie und ein Asthma bronchiale bekannt.
Nenne die hypothetischen Therapieoptionen in einer Tabelle. Nutze als Zeilen Operative Therapie, Chemotherapie, Strahlentherapie, Anti-Her2-Therapie und endokrine Therapie. Nutze als Spalten Empfehlung (ja/nein), das hypothetische Medikament (Wirkstoff und Dosierung), Alternativen und Risiken. Verwende in der Tabelle nur Stichpunkte.
Halte dich so kurz wie möglich, wiederhole nicht den Fall oder die Frage. Sei präzise. Fasse die tabellarische Empfehlung in einem ausgeschriebenen Satz unter der Tabelle zusammen.

Fall 30 – Empfehlung ChatGPT:

Therapieoptionen	Empfehlung	Medikament (Wirkstoff und Dosierung)	Alternativen	Risiken
Operative Therapie	Nein	Keine operative Therapie aufgrund des fortgeschrittenen Stadiums und Exulzeration	-	-
Chemotherapie	Ja	Anthrazyklin-basierte Chemotherapie (z.B. EC: Epirubicin + Cyclophosphamid) gefolgt von Taxanen (z.B. Paclitaxel)	-	Übelkeit, Haarausfall, Immunsuppression
Strahlentherapie	Ja	Palliative Strahlentherapie bei lokalen Beschwerden	-	Hautreizungen, Müdigkeit, selten Langzeiteffekte
Anti-HER2-Therapie	Nein	Keine Anti-HER2-Therapie aufgrund des HER2-negativen Status	-	-
Endokrine Therapie	Nein	Keine endokrine Therapie aufgrund des hormonrezeptor-negativen Status	-	-

Die empfohlene Therapieoption für die 60-jährige postmenopausale Patientin mit einem fortgeschrittenen Mammakarzinom rechts mit Exulzeration in der Submammärfalte umfasst eine Chemotherapie mit Anthrazyklinen (z.B. Epirubicin + Cyclophosphamid) gefolgt von Taxanen (z.B. Paclitaxel) zur Reduktion des Tumorwachstums. Eine palliative Strahlentherapie kann zur Linderung von lokalen Beschwerden eingesetzt werden. Aufgrund des HER2-negativen und hormonrezeptor-negativen Status sind eine Anti-HER2-Therapie und eine endokrine Therapie nicht empfohlen. Risiken und Alternativen wurden berücksichtigt.

Die empfohlene Therapie für die 60-jährige postmenopausale Patientin mit einem fortgeschrittenen Mammakarzinom rechts mit Exulzeration in der Submammärfalte umfasst eine Chemotherapie mit Anthrazyklinen (z.B. Epirubicin + Cyclophosphamid) gefolgt von Taxanen (z.B. Paclitaxel) zur Reduktion des Tumorwachstums. Eine palliative Strahlentherapie kann zur Linderung von lokalen Beschwerden eingesetzt werden. Aufgrund des HER2-negativen und hormonrezeptor-negativen Status sind eine Anti-HER2-Therapie und eine endokrine Therapie nicht empfohlen. Risiken und Alternativen wurden berücksichtigt. Eine operative Therapie ist aufgrund des fortgeschrittenen Stadiums und der Exulzeration nicht empfohlen.

Translated version:

Case 30

Medical History:
60 years old, postmenopausal
Medical conditions:
Diabetes mellitus (treated with Metformin)
Arterial hypertension
Bronchial asthma
Coxarthrosis with chronic pain

Current Diagnosis:
1 month ago, diagnosed with advanced breast cancer on the right side with ulceration in the submammary fold.
Tumor stage: cT4b, multicentric findings, cN1, M1 (lung metastasis in the right upper lobe)
Lesion 1: 14 mm in the axillary extension
Lesion 2: upper outer area, measuring 36 x 28 mm
Lesion 3: in the submammary fold at 6 o'clock, measuring 36 x 28 mm, N1 (at least 3 lymph nodes, max. 23 mm)
ER 0%; PR 0%; HER2-negative; Ki67 75%, PD-L1 TPS 0% and IC 1

Prompt:
Please, based on the following URL: https://www.leitlinienprogramm-onkologie.de/fileadmin/user_upload/Downloads/Leitlinien/Mammakarzinom_4_0/Version_4.4/LL_Mammakarzinom_Langversion_4.4.pdf, provide the recommended treatment for a hypothetical 60-year-old postmenopausal patient with advanced breast cancer on the right side with ulceration in the submammary fold. Use the current tumor formula: cT4b, multicentric findings, cN1, M1, ER 0%; PR 0%; HER2-negative; Ki67 75%, PD-L1 TPS 0%, and IC 1.
No treatment has been administered so far.
As pre-existing conditions, diabetes mellitus, arterial hypertension, and bronchial asthma are known.
List the hypothetical treatment options in a table. Use rows for Surgical Therapy, Chemotherapy, Radiotherapy, Anti-HER2 Therapy, and Endocrine Therapy. Use columns for Recommendation (yes/no), hypothetical medication (substance and dosage), Alternatives, and Risks. Use bullet points in the table.
Keep it as concise as possible, avoid repeating the case or the question. Be precise. Summarize the tabular recommendation in a written sentence below the table.

Case 30 – Recommendation ChatGPT

**Therapy options**	**Recommendation**	**Medication (Active agent and dosage)**	**Alternatives**	**Risks**
Surgery	No	No surgery due to the advanced tumor stage and the ulceration	-	-
Chemotherapy	Yes	Anthracycline-based chemotherapy (e.g. EC: Epirubicin + Cyclophosphamide) followed by taxanes (e.g. Paclitaxel)	-	Immunosuppression, nausea, hair loss

Radiation	Yes	Palliative radiotherapy for localized issues	-	Skin reaction, fatigue, rare long-term complications
Anti-HER2-Therapy	No	No anti-HER2 therapy due to HER2 negative status	-	-
Endocrine therapy	No	No endocrine therapy due to hormone negative status	-	-

The recommended treatment option for the 60-year-old postmenopausal patient with advanced breast cancer on the right side with ulceration in the submammary fold includes chemotherapy with anthracyclines (e.g., Epirubicin + Cyclophosphamide) followed by taxanes (e.g., Paclitaxel) to reduce tumor growth. Palliative radiotherapy may be used to alleviate local symptoms. Due to the HER2-negative and hormone receptor-negative status, anti-HER2 therapy and endocrine therapy are not recommended. Risks and alternatives have been considered.

The recommended treatment for the 60-year-old postmenopausal patient with advanced breast cancer on the right side with ulceration in the submammary fold includes chemotherapy with anthracyclines (e.g., Epirubicin + Cyclophosphamide) followed by taxanes (e.g., Paclitaxel) to reduce tumor growth. Palliative radiotherapy may be used to alleviate local symptoms. Due to the HER2-negative and hormone receptor-negative status, anti-HER2 therapy and endocrine therapy are not recommended. Risks and alternatives have been considered. Surgical intervention is not recommended due to the advanced stage and ulceration.
