# Supplementary material for: Quality of ChatGPT-Generated Therapy Recommendations for Breast Cancer Treatment in Gynecology
Source: Curr Oncol. 2024 Jul 1;31(7):3845–54. doi: 10.3390/curroncol31070284 (PMC11275284; doi:10.3390/curroncol31070284)
Supplement: Supplementary file 1 [file curroncol-31-00284-s001.zip › File S2_Evaluation form.pdf]

Bitte evaluieren Sie die Empfehlung durch ChatGPT für den zugehörigen Fall.

Fall X

Therapiesäule	Gänzlich korrekt	Korrekt mit Einschränkungen	Bessere Alternative verfügbar	Anteilig falsch	Gänzlich falsch
Operative Therapie	☐	☐	☐	☐	☐
Chemotherapie (Substanz + Dosierung)	☐	☐	☐	☐	☐
Endokrine Therapie (Substanz + Therapiedauer)	☐	☐	☐	☐	☐
Strahlentherapie	☐	☐	☐	☐	☐
Antikörper-Therapie	☐	☐	☐	☐	☐
Alternative Therapie	☐	☐	☐	☐	☐
Gesamtempfehlung	☐	☐	☐	☐	☐

Translated version

Please evaluate the therapy recommendation generated by ChatGPT for the corresponding case.

Case X

Therapy option	Correct without limitations	Correct with minor limitations	Better alternative available	Partially wrong	Completely wrong
Surgical Therapy	☐	☐	☐	☐	☐
Chemotherapy (Medication + dosage)	☐	☐	☐	☐	☐
Endocrine therapy (Medication + therapy interval)	☐	☐	☐	☐	☐
Radiotherapy	☐	☐	☐	☐	☐
Antibody therapy	☐	☐	☐	☐	☐
Therapy alternatives	☐	☐	☐	☐	☐
Overall recommendation	☐	☐	☐	☐	☐
